# Supplementary material for: Development of Molecular Markers for Predicting Radish (Raphanus sativus) Flesh Color Based on Polymorphisms in the RsTT8 Gene
Source: Plants (Basel). 2021 Jul 6;10(7):1386. doi: 10.3390/plants10071386 (PMC8309288; doi:10.3390/plants10071386)
Supplement: Supplementary file 1 [file plants-10-01386-s001.zip › Figure S1.pdf]

***RsTT8* genomic DNA**

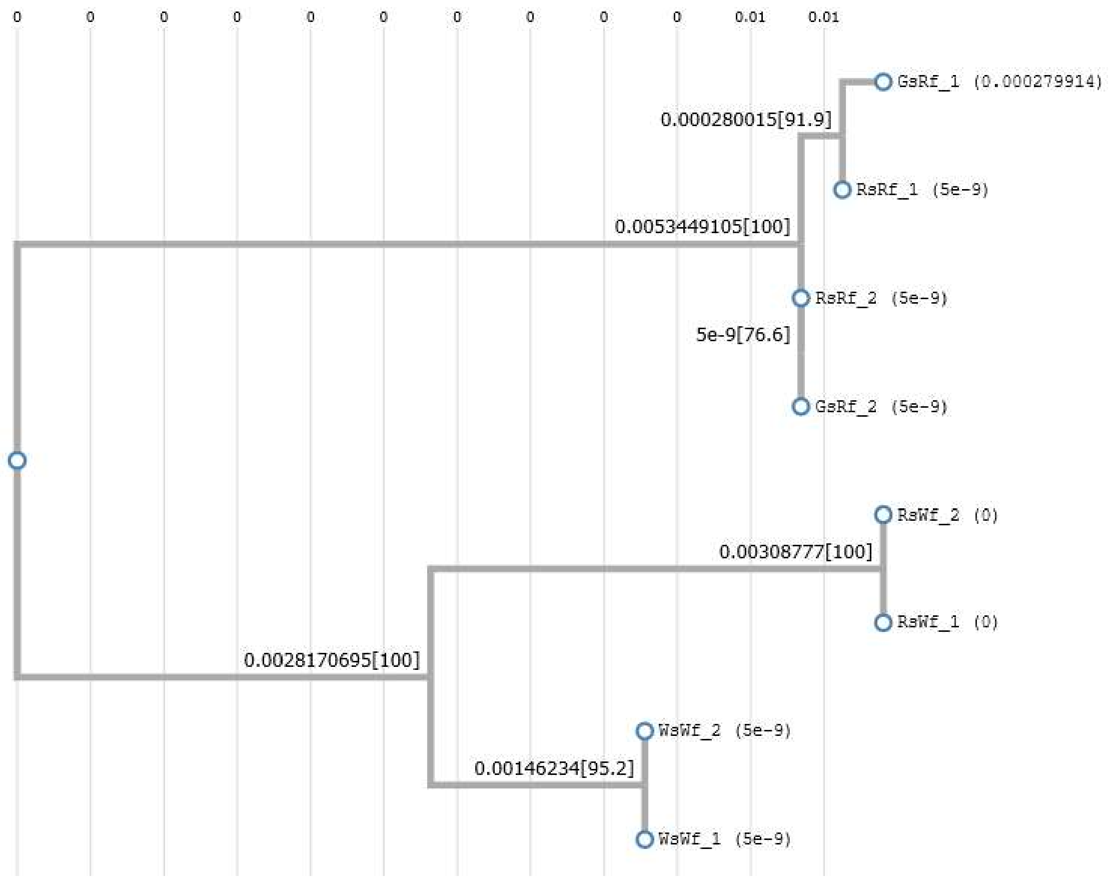

Region	Between Rf and Wf			WsWf specific			RsWf specific		
	SNP	InDel	SSR	SNP	InDel	SSR	SNP	InDel	SSR
Promoter	16	2	1	2	4	1	2	-	-
Exon	2	-	-	1	-	-	3	1	-
Intron	10	1	2	3	3	2	4	-	1
Total	28	3	3	6	7	3	9	1	1

Nucleotide sequences

>RsWf_1
CACAGCTAGCCGCATCAATATGTTAGCAACTGAAGAAACATTGATTAGCGGACAATCGGGAAGGAAAATCAAGATCGAGCAACAAGCGTTTGCACTCAAGAATTTTATTTTATTTTTTTT
GTTCGGGCACCATGGCCATAAGGATTAAATGATCATCGTCCCACACATGGACAGAATTTGGACGCACCCAGTCAATCCAAGTGGCCGTTAGTATAAAACATAATCCTGAAGCACAAGCCA
AATAACTTTATTTATTGGTCGACAAAACCGCCAAGAAACGTTTAATGTTGCTTTTGTCTAAACCAGAATACATCTCACATACCACATACCCAACTGTATAAACTATATGTGTACGTAACT
GTATCTATGTGTCTATCAAATTTTTATATGTTTCTGGGGTCAGCTATGTCTAACTTTATTGAGTTTTCCAAGACATTTCAAGGGAAACAGATGGTGGCTACTTGGCTAGCAATTCACATC
AATTATTAAAAACTTGAATTGTGAAACATCTCATTCTCCTCCTTTATTACACCAAACTACTTTCTTTTCTCTACTTACCGGTCAGGTCAACAGTTCTACCATTCCCCTTCTTCTTTTTAC
TCTTTTCGTTTAAATAAAACTAAAGATGCTACTCTTTTATTATGGTTGATAAACACTGGAATATTGCAAATTTCCAAATAAGAGAAAAGAAAGAGAGATGTAAAACCGAAATCATAGTAA
CACAAGTCAAAGCAACGTCTGTTTTCTCTTGTTTGTCAGTTAGCTATTTTCTCTACTAAAATATGTTATGCTTTTTTCCCAATAATATATTATAACATATTAAGAAATATGCATTCGGCA
TTTTTGGTTCTCCAATCCGTGGGGGACACGATTACAAGACCATTTCATCTTTTTATTTCATCTGTATTCTACTCTCTCATCCGACGTCTGGTTTACCAACCAGTCAGAACGCATAAAAGT
CAACTTTTACTAAATCTTGAGTTTTTGGTCATGCATTTATACACATACATAGAATTAATCTAGATTACTTTACTTATGTCAGCATGTTACGAAAACACTAAAATGGTATATATATGCATA
CATATTGAATTAACCGTGGTCCCAAACCAATTTAAATCAGCATAAAGAATATCTTCCTCTTTCATATCAAAACCCCTTCAGAGTTGCAAGATCTTACTCTCTTCAGAGTTGCGAGAGAGA
GCTAATATATGTTTTCATCTCGGGGAAAAAATGGATGAATCAAGTATTATACCGGTATGGAAAGTGATCGGAGCTGAGGGAAAAGAGATTCAAGGGCTACTTAAGGCGGTGGTGCAATCTG
TGGGGTGGACTTATAGTCTCTTCTGGCAACTTTGTCCTCAACGAAGGTTCTCTTTTCATTTCATCCATCTCTCACAATATATAAAGCAATATATTAATTTATCCTTATTAATTATAACAA
GTAGAAGTGACTAAATGTTAAATCGATTAGGAAATTGGTGTGGAGTAGTGGATTCTACAACGGTGCAATAAAGACTAGAAAGACAACTCAGCCGGCGGAAATAACGGCTGAAGAGGCTGC
GTTGGAGAGAAGCCAACAGCTCATGGAGCTTTACCAGACGCTTTTTGCCGGAGAATCATCGATGGAAGCGAGGGCTTCCACAGCACTGTCGCCTGAGGATTTGACGGACACTGAATGGTT
TTATGTGCTGTGTCTCACTTACTCTTTTGAACCTCCTTCTGGGTACAACAACTCTTTCTCTCTCTCTCTCTCTCTCTCTCTCCAAAGTTTTTTCTTTCTTTCTATAAAGACTACTCAGAG
TTTCTTAATTTGTCCTTTTCATCTCCTCTTAGTGAAGACAAAAATAGTATTGTGTGTTAAATGCGAATCGAAAATACTATGGAGCATTAAAGACAAACTGAGGAGTTTAAGTTACTGAAA
GAAGAAATTTATTGAAGTTTGTGAAAACGTACACTTCATTTTGGTGAACATAATTGGACCGTTGAGATTCGTATTGGTTTGTTTATTGATTATCTAAAGTAGAACTGTAGAAGCATATAA
AAAATGCATAACAAAGTGTGTTAGTTATCGTTATAATTAATGTTTTTTTCTCTATGGAGGAGAAAAAAAGATCAAAATATAAATGTAGAAGTATTAATTTGTAGGATGCCAGGAAAGGCG
TATGCGAGGAGGAAGCAAGTATGGATGAGTGGTGTAAATGAGGTTGACAGTAAAATCTTCTCTAGGGCTATTTCTGCAAAGGTTTATTTCCTTTTATTCATTCACCACTACACTGTGTAT
CTACTTCTACTTATTTAGATATACGAAATTTTATATTATCTCATTTCAAACTAATTAATTTTATCTTCTTAATGCTCTTTCTACACTAGAGTGCCAAAATTCAGGTAAATGTTGCCGTTA
TTATTTTATTTTCGTAAAAATGAAAGGTATCAGTTAACAGAGTTATTATTTTACATATTTGAGAATTGTTTTGTGATGAAAAAACAAATAAAAACAGACAGTGGTTTGCATTCCCGTGCT
TGATGGCGTTTTGGAAATAGGCACAACGAACAAGGTAATAAAAGCCTATTTATGTCGGTACCAACAAGTGTAAACGAATACATTAGCTCGTTCTATGTTATATAGATAAATAAAGAAAAA
TAATGTACAGAACATCAACCATTCGCAGTCATATGATTAAAATATAGTTTTTCGATACATTATATACACAACCAAATGATGTAAAAACCTAATCAAAAATATGCATAAGAGGGAGAACAA
AGCCGGAGATGGATGCGTGATATGTTGTGTTTTATGTGAAGGTCAAAGAAAATGAAGAGTTTGTTGAACACATAAAGAGTTTCTTCCAAAACCACCCGAAGTCAAACACGAAGCCTGCTC
TTTTTGAACACTCCATCAACGAAGATCATGAAGAAGACGAAGAAGAAGTAGAAGAAATGACAATGTCAGAGGAGATAAGACTTGGTTCTCCTGATGACGATGACGTCTCCAATCAAAATC
TACTCTCTGATTTCCATATAGAAGCACCCAATAGTTTAGGTATGCCGTACACCTTTCTTATTAGATTAAAATAGTTAACAAGATCATTTTAACTAATTTTCTAATAAATTTTTTTTTGAA
ATGTTTCACATGTCTGGTAATTTTAACATATATCATTGTATATATAGATACACAAATGGACATGATGAATCTAATGGAGGAAGGCGGAAATTATTCTCAGACAGTATCAACACTTCTCAT
GTCACAACTCCCCAATCTTCTTTCAGATTCAGTTTCCACATCTTCTTACGTTCAATCATCGTTTGTCTCGTGGAGGGTTGAGAATGTCAAAGAGCATCAGCAATATCAACGAGAGGAGAA
AGCGTCGTCGTCATCCTCGTCGCAATGGATGCTCAAACACATGATCTTGAGAGTTCCTTTACTCCATGAAAACACTAAAAACAAGAGGGTGCCGCGGGAAGAGCTCAACCATGTGGTGGC
CGAGCGACGCAGAAGAGAGAAGCTTAACGAGAGATTCATAACGTTGAGATCATTGGTTCCATTTGTGACCAAGATGGATAAAGTCTCGATCCTTGGAGACACCATTGATTACGTAAACCA
TCTTTGTAAGAGGATCCATGAGCTGGAATCTACTCATCACGAGCCAAACCAAAAGCGGATGCGTATCGGTAAGGGAAGAACGTGGGAAGAGGTGGAGGTTTCCATTATAGAGAGCGATGT
TTTGTTAGAGATGAGATGCGAGTACCGAGATGGTTTATTGCTCAACATTCTTCAGGTACTTAAGGAGCTGGGTATAGAGACCACTGCAGTTCACACCGCCGTGAACGACCATGATTTTGA
GGCAGAGATAAGGGCGAAAGTGAGAGGGAAGAAACCAACCATTGCTGAGGTTAAAATAGCCATCCATCAAATCATATCTCAAAATAAACTCTAG

>RsWf_2
CACAGCTAGCCGCATCAATATGTTAGCAACTGAAGAAACATTGATTAGCGGACAATCGGGAAGGAAAATCAAGATCGAGCAACAAGCGTTTGCACTCAAGAATTTTATTTTATTTTTTTT
GTTCGGGCACCATGGCCATAAGGATTAAATGATCATCGTCCCACACATGGACAGAATTTGGACGCACCCAGTCAATCCAAGTGGCCGTTAGTATAAAACATAATCCTGAAGCACAAGCCA
AATAACTTTATTTATTGGTCGACAAAACCGCCAAGAAACGTTTAATGTTGCTTTTGTCTAAACCAGAATACATCTCACATACCACATACCCAACTGTATAAACTATATGTGTACGTAACT
GTATCTATGTGTCTATCAAATTTTTATATGTTTCTGGGGTCAGCTATGTCTAACTTTATTGAGTTTTCCAAGACATTTCAAGGGAAACAGATGGTGGCTACTTGGCTAGCAATTCACATC
AATTATTAAAAACTTGAATTGTGAAACATCTCATTCTCCTCCTTTATTACACCAAACTACTTTCTTTTCTCTACTTACCGGTCAGGTCAACAGTTCTACCATTCCCCTTCTTCTTTTTAC
TCTTTTCGTTTAAATAAAACTAAAGATGCTACTCTTTTATTATGGTTGATAAACACTGGAATATTGCAAATTTCCAAATAAGAGAAAAGAAAGAGAGATGTAAAACCGAAATCATAGTAA
CACAAGTCAAAGCAACGTCTGTTTTCTCTTGTTTGTCAGTTAGCTATTTTCTCTACTAAAATATGTTATGCTTTTTTCCCAATAATATATTATAACATATTAAGAAATATGCATTCGGCA
TTTTTGGTTCTCCAATCCGTGGGGGACACGATTACAAGACCATTTCATCTTTTTATTTCATCTGTATTCTACTCTCTCATCCGACGTCTGGTTTACCAACCAGTCAGAACGCATAAAAGT
CAACTTTTACTAAATCTTGAGTTTTTGGTCATGCATTTATACACATACATAGAATTAATCTAGATTACTTTACTTATGTCAGCATGTTACGAAAACACTAAAATGGTATATATATGCATA
CATATTGAATTAACCGTGGTCCCAAACCAATTTAAATCAGCATAAAGAATATCTTCCTCTTTCATATCAAAACCCCTTCAGAGTTGCAAGATCTTACTCTCTTCAGAGTTGCGAGAGAGA
GCTAATATATGTTTTCATCTCGGGGAAAAAATGGATGAATCAAGTATTATACCGGTATGGAAAGTGATCGGAGCTGAGGGAAAAGAGATTCAAGGGCTACTTAAGGCGGTGGTGCAATCTG
TGGGGTGGACTTATAGTCTCTTCTGGCAACTTTGTCCTCAACGAAGGTTCTCTTTTCATTTCATCCATCTCTCACAATATATAAAGCAATATATTAATTTATCCTTATTAATTATAACAA
GTAGAAGTGACTAAATGTTAAATCGATTAGGAAATTGGTGTGGAGTAGTGGATTCTACAACGGTGCAATAAAGACTAGAAAGACAACTCAGCCGGCGGAAATAACGGCTGAAGAGGCTGC
GTTGGAGAGAAGCCAACAGCTCATGGAGCTTTACCAGACGCTTTTTGCCGGAGAATCATCGATGGAAGCGAGGGCTTCCACAGCACTGTCGCCTGAGGATTTGACGGACACTGAATGGTT
TTATGTGCTGTGTCTCACTTACTCTTTTGAACCTCCTTCTGGGTACAACAACTCTTTCTCTCTCTCTCTCTCTCTCTCTCTCCAAAGTTTTTTCTTTCTTTCTATAAAGACTACTCAGAG
TTTCTTAATTTGTCCTTTTCATCTCCTCTTAGTGAAGACAAAAATAGTATTGTGTGTTAAATGCGAATCGAAAATACTATGGAGCATTAAAGACAAACTGAGGAGTTTAAGTTACTGAAA
GAAGAAATTTATTGAAGTTTGTGAAAACGTACACTTCATTTTGGTGAACATAATTGGACCGTTGAGATTCGTATTGGTTTGTTTATTGATTATCTAAAGTAGAACTGTAGAAGCATATAA
AAAATGCATAACAAAGTGTGTTAGTTATCGTTATAATTAATGTTTTTTTCTCTATGGAGGAGAAAAAAAGATCAAAATATAAATGTAGAAGTATTAATTTGTAGGATGCCAGGAAAGGCG
TATGCGAGGAGGAAGCAAGTATGGATGAGTGGTGTAAATGAGGTTGACAGTAAAATCTTCTCTAGGGCTATTTCTGCAAAGGTTTATTTCCTTTTATTCATTCACCACTACACTGTGTAT
CTACTTCTACTTATTTAGATATACGAAATTTTATATTATCTCATTTCAAACTAATTAATTTTATCTTCTTAATGCTCTTTCTACACTAGAGTGCCAAAATTCAGGTAAATGTTGCCGTTA
TTATTTTATTTTCGTAAAAATGAAAGGTATCAGTTAACAGAGTTATTATTTTACATATTTGAGAATTGTTTTGTGATGAAAAAACAAATAAAAACAGACAGTGGTTTGCATTCCCGTGCT
TGATGGCGTTTTGGAAATAGGCACAACGAACAAGGTAATAAAAGCCTATTTATGTCGGTACCAACAAGTGTAAACGAATACATTAGCTCGTTCTATGTTATATAGATAAATAAAGAAAAA
TAATGTACAGAACATCAACCATTCGCAGTCATATGATTAAAATATAGTTTTTCGATACATTATATACACAACCAAATGATGTAAAAACCTAATCAAAAATATGCATAAGAGGGAGAACAA
AGCCGGAGATGGATGCGTGATATGTTGTGTTTTATGTGAAGGTCAAAGAAAATGAAGAGTTTGTTGAACACATAAAGAGTTTCTTCCAAAACCACCCGAAGTCAAACACGAAGCCTGCTC
TTTTTGAACACTCCATCAACGAAGATCATGAAGAAGACGAAGAAGAAGTAGAAGAAATGACAATGTCAGAGGAGATAAGACTTGGTTCTCCTGATGACGATGACGTCTCCAATCAAAATC
TACTCTCTGATTTCCATATAGAAGCACCCAATAGTTTAGGTATGCCGTACACCTTTCTTATTAGATTAAAATAGTTAACAAGATCATTTTAACTAATTTTCTAATAAATTTTTTTTTGAA
ATGTTTCACATGTCTGGTAATTTTAACATATATCATTGTATATATAGATACACAAATGGACATGATGAATCTAATGGAGGAAGGCGGAAATTATTCTCAGACAGTATCAACACTTCTCAT
GTCACAACTCCCCAATCTTCTTTCAGATTCAGTTTCCACATCTTCTTACGTTCAATCATCGTTTGTCTCGTGGAGGGTTGAGAATGTCAAAGAGCATCAGCAATATCAACGAGAGGAGAA
AGCGTCGTCGTCATCCTCGTCGCAATGGATGCTCAAACACATGATCTTGAGAGTTCCTTTACTCCATGAAAACACTAAAAACAAGAGGGTGCCGCGGGAAGAGCTCAACCATGTGGTGGC
CGAGCGACGCAGAAGAGAGAAGCTTAACGAGAGATTCATAACGTTGAGATCATTGGTTCCATTTGTGACCAAGATGGATAAAGTCTCGATCCTTGGAGACACCATTGATTACGTAAACCA
TCTTTGTAAGAGGATCCATGAGCTGGAATCTACTCATCACGAGCCAAACCAAAAGCGGATGCGTATCGGTAAGGGAAGAACGTGGGAAGAGGTGGAGGTTTCCATTATAGAGAGCGATGT
TTTGTTAGAGATGAGATGCGAGTACCGAGATGGTTTATTGCTCAACATTCTTCAGGTACTTAAGGAGCTGGGTATAGAGACCACTGCAGTTCACACCGCCGTGAACGACCATGATTTTGA
GGCAGAGATAAGGGCGAAAGTGAGAGGGAAGAAACCAACCATTGCTGAGGTTAAAATAGCCATCCATCAAATCATATCTCAAAATAAACTCTAG

>GsRf_1
CACAGCTAGCCGCATCAATATGTTAGCAACTGAAGAAACATTGATTAGCGGACAATCGGGAAGGAAAATCAAGATCGAGCAACAAGCGTTTGCACTCAAGAATTTTATTTTATTTATTTT
GTTCGGGCACCATGGCCATAAGGATTAAATGATCATCGTCCCACACATGGACAGAATTTGGACGCACCCAGTCAATCCAAGTGGCCGTTAGTATAAAACATAATCCTGAAGCACAAGCCA
AATAACTTTATTTATTGGTCGACAAAAAAGGCCAAGAACGTTTAATGTTGCTTTTGTCTAAACCAGGATACATCTCACATACCACATACCCAACTGTATAAACTATGTGTACGTAACTGT
ATCTATGTGTCTATCAAAATTTTATATGTTTCTCGGGTCAGCTATATCTAACTTTATTGAGTTTTCCAAGACATTACAAGGGAAACAGATGGTGGCTACTTGGCTAGCAATTCACATCAA
TTATTAAAAACTTGAATTGTGAAACATCTCATTCTCCTCCTTTATTACACCAAACTACTTTCTTTTCTCTACTTACCGGTCAGGTCAACAGTTCTACCATTCCCCTTCTTCTTTTTACTC
TTTTCGTTTAAATAAAACTAAAGATGCTACTCTTTTATTATGGTTGATAAACACTGGAATATTGCAAATTTCCAAATAAGAGAAAAGAAAGAGAGATATAAAACCGAAATCATAGTAACA
CAAGTCAAAGCAACGTCTGTTTTCTCTTGGTTGTCAGTTAGCTATTTTCTCTACTGAAATATGTTATGCTTTTTTCCCAATAATATATTATAACATATTAAAAAATATGCATTCGGCATT
TTTGGTTCTCCAATCAGTGGGGGACACGATTACAAGACAATTACATCTTTTTATTTCATCTGTATTCTACTCTCTCATCCAACGTCTGGTTTACCAACCAGTCAGAACGCATAAAAGTCA
ACTTTTACTAAATCTTGAGCTTTTTGGTCATGCATTTATACACATACATAGAATTAATCTAGATTACTTTACTTATGTCAGCATGTTACGAAAACACTAAAATGGTATATATATACATAC
ATATTGAATTAACCGTGGTCCCAAACCAATTTAAATCAGCATAAAGAATATCTTCCTCTTTCATATCAAAACCCCTTCAGAGTTGCAAGATCTTACTCTCAGAGAGCTAATACATGTTTT

CATCTCGGGGAAAAATGGATGAATCAAGTATTATACCGGTATGGAAAGTGATCGGAGCTGAGGAAAAAGAGATTCAAGGGCTACTTAAGGCGGTGGTGCAATCTGTGGGGTGGACTTATA
GTCTCTTCTGGCAACTTTGTCCTCAACGAAGGTTCTCTTTTCATTTCATCCATCTCTCACAATATATAAAGCAATATATTAATTTATCCTTATTAATTATAACAAGTAGAAGTGACTAAT
GTTAAATCGATTAGGAAATTGGTGTGGAGTAGTGGATTCTACAACGGTGCAATAAAGACTAGAAAGACAACTCAGCCGGCGGAAATAACGGCTGAAGAGGCTGCGTTGGAGAGAAGCCAA
CAGCTCATGGAGCTTTACCAGACGCTTTTTGCCGGAGAATCATCGATGGAAGCGAGGGCTTGCACAGCACTGTCGCCTGAGGATTTGACGGACACTGAATGGTTTTATGTGCTGTGTCTC
ACTTACTCTTTTGAACCTCCTTCTGGGTACAACAACTCTTTCTCTCTCTCTCTCTCTCTCTCTCTCTCTCTCTCTCTCTCTCTCTCTCTCTCTCTCTCTCTCTCTCTCTCTCTCTCTCCAAAGT
TTTTTCTTTCTTTCTATAAAGACTACTCAGAGTTTCTTAATTTGTCCTTTTCATCTCCTCTTAGTGAAGACAAAAATAGTATTGTGTGTTAAATGCGAATCGAAAATACTATGGAGCATT
AAAGACAAACTGAGGAGTTTAAGTTACTGAAAGAAGAAATTTATTGAAGTTTGTGAAAACGTACACTTCATTTTGGTGAACATAATTGGACCGTTGAGATTCGTATTGGTTTGTTTATTG
ATTATCTAAAGTAGAACTGTAGAAGCATATAAAAAATGCATAACAAAGTGTGTTAGTTATCGTTATAATTAATGTTTTTTTCTCTATGGAGGAGAAAAAAAAATCAAAATATAAATGTAG
AAGTATTAATTTGTAGGATGCCAGGAAAGGCGTATGCGAGGAGGAAGCAAGTATGGATGAGTGGTGTAAATGAGGTTGACAGTAAAATCTTCTCTAGGGCTATTTCTGCAAAGGTTTATT
TCCTTTTATTCATTCACCACTACACTGTGTATCTACTTCTACTTATTTAGATATACGAAATTTTATATTATCTCATTTCAAACTAATTAATTTTATCTTCTTAATGCTCTTTCTACACTA
GAGTGCCAAAATTCAGGTAAATGTTGCCGTTATTATTTTATTTTCGTAGAAATGAAAGGTATCAGTTAACAGAGTTATTATTTTACATATTTGAGAATTGTTTTGTGATGAAAAAACAAA
TAAAAACAGACAGTGGTTTGCATTCCCGTGCTTGATGGCGTTTTGGAAATAGGCACAACGAACAAGGTAATAAAAGTCTATTTATGTCGGTACCAACAAGTGTAAACGAATACATTAGCT
CGTTCTATGTTACATAGATAAATAAAGAAAAATAATGTACAGAACATCATCAACCATTCGCAGTCATATGATTAAAATATAGTTTTTCGATACATTATATACACAACCAAATGATGTAAA
AACCTAATCAAAAATATGCATAAGAGGGAGAACCAAGCCGGAGATGGATGCGTGATATGTTGTGTTTTATGTGAAGGTCAAAGAAAATGAAGAGTTTGTTGAACACATGAAGAGTTTCTT
CCAAAACCACCCGAAGTCAAACACGAAGCCTGCTCTTTTTGAACACTCCATCAACGAAGAGCATGAAGAAGACGAAGAAGTAGAAGAAATGACAATGTCAGAGGAGATAAGACTTGGTTC
TCCTGATGACGATGACGTCTCCAATCAAAATCTACTCTCTGATTTCCATATAGAAGCACCCAGTAGTTTAGGTATGCCGTGCACATTTCTTATTAGATTAAATAGTTAACAAGATCATTT
TAATTAATTTTCTAATAATAAAATTTTTGAAATGTTTCACATGTCTAGTAATTTTAACATATATCATTGTATATATAGATACACAAATGGACATGATGAATCTAATGGAGGAAGGCGGAA
ATTATTCTCAGACAGTATCAACACTTCTCATGTCACAACTCCCCAATCTTCTTTCAGATTCAGTTTCCACATCTTCTTACGTTCAATCATCGTTTGTCTCGTGGAGGGTTGAGAATGTCA
AAGAGCATCAGCAATATCAACGAGAGGAGAAAGCGTCGTCGTCATCCTCGTCGCAATGGATGCTCAAACACATGATCTTGAGAGTTCCTTTACTCCATGAAAACACTAAAAACAAGAGGG
TGCCGCGGGAAGAGCTCAACCATGTGGTGGCCGAGCGACGCAGAAGAGAGAAGCTTAACGAGAGATTCATAACGTTGAGATCATTGGTTCCATTTGTGACCAAGATGGATAAAGTCTCGA
TCCTTGGAGACACCATTGATTACGTAAACCATCTTTGTAAGAGGATCCATGAGCTGGAATCTACTCATCACGAGCCAAACCAAAAGCGGATGCGTATCGGTAAGGGAAGAACGTGGGAAG
AGGTGGAGGTTTCCATTATAGAGAGCGATGTTTTGTTAGAGATGAGATGCGAGTACCGAGATGGTTTATTGCTCAACATTCTTCAGGTACTTAAGGAGCTGGGTATAGAGACCACTGCAG
TTCACACCGCCGTGAACGACCATGATTTTGAGGCAGAGATAAGGGCGAAAGTGAGAGGGAAGAAACCAACCATTGCTGAGGTTAAAATAGCCATCCATCAAATCATATCTCAAAATAAAC
TCTAG

>GsRf_2
CACAGCTAGCCGCATCAATATGTTAGCAACTGAAGAAACATTGATTAGCGGACAATCGGGAAGGAAAATCAAGATCGAGCAACAAGCGTTTGCACTCAAGAATTTTATTTTATTTATTTT
GTTCGGGCACCATGGCCATAAGGATTAAATGATCATCGTCCCACACATGGACAGAATTTGGACGCACCCAGTCAATCCAAGTGGCCGTTAGTATAAAACATAATCCTGAAGCACAAGCCA
AATAACTTTATTTATTGGTCGACAAAAAAGGCCAAGAACGTTTAATGTTGCTTTTGTCTAAACCAGGATACATCTCACATACCACATACCCAACTGTATAAACTATGTGTACGTAACTGT
ATCTATGTGTCTATCAAAATTTTATATGTTTCTCGGGTCAGCTATATCTAACTTTATTGAGTTTTCCAAGACATTACAAGGGAAACAGATGGTGGCTACTTGGCTAGCAATTCACATCAA
TTATTAAAAACTTGAATTGTGAAACATCTCATTCTCCTCCTTTATTACACCAAACTACTTTCTTTTCTCTACTTACCGGTCAGGTCAACAGTTCTACCATTCCCCTTCTTCTTTTTACTC
TTTTCGTTTAAATAAAACTAAAGATGCTACTCTTTTATTATGGTTGATAAACACTGGAATATTGCAAATTTCCAAATAAGAGAAAAGAAAGAGAGATATAAAACCGAAATCATAGTAACA
CAAGTCAAAGCAACGTCTGTTTTCTCTTGTTTGTCAGTTAGCTATTTTCTCTACTGAAATATGTTATGCTTTTTTCCCAATAATATATTATAACATATTAAGAAATATGCATTCGGCATT
TTTGGTTCTCCAATCAGTGGGGGACACGATTACAAGACAATTACATCTTTTTATTTCATCTGTATTCTACTCTCTCATCCAACGTCTGGTTTACCAACCAGTCAGAACGCATAAAAGTCA
ACTTTTACTAAATCTTGAGCTTTTTGGTCATGCATTTATACACATACATAGAATTAATCTAGATTACTTTACTTATGTCAGCATGTTACGAAAACACTAAAATGGTATATATATACATAC
ATATTGAATTAACCGTGGTCCCAAACCAATTTAAATCAGCATAAAGAATATCTTCCTCTTTCATATCAAAACCCCTTCAGAGTTGCAAGATCTTACTCTCAGAGAGCTAATACATGTTTT
CATCTCGGGGAAAAATGGATGAATCAAGTATTATACCGGTATGGAAAGTGATCGGAGCTGAGGAAAAAGAGATTCAAGGGCTACTTAAGGCGGTGGTGCAATCTGTGGGGTGGACTTATA
GTCTCTTCTGGCAACTTTGTCCTCAACGAAGGTTCTCTTTTCATTTCATCCATCTCTCACAATATATAAAGCAATATATTAATTTATCCTTATTAATTATAACAAGTAGAAGTGACTAAT
GTTAAATCGATTAGGAAATTGGTGTGGAGTAGTGGATTCTACAACGGTGCAATAAAGACTAGAAAGACAACTCAGCCGGCGGAAATAACGGCTGAAGAGGCTGCGTTGGAGAGAAGCCAA
CAGCTCATGGAGCTTTACCAGACGCTTTTTGCCGGAGAATCATCGATGGAAGCGAGGGCTTGCACAGCACTGTCGCCTGAGGATTTGACGGACACTGAATGGTTTTATGTGCTGTGTCTC
ACTTACTCTTTTGAACCTCCTTCTGGGTACAACAACTCTTTCTCTCTCTCTCTCTCTCTCTCTCTCTCTCTCTCTCTCTCTCTCTCTCTCTCTCTCTCTCTCTCTCTCTCCAAAGTTTTT
TCTTTCTTTCTATAAAGACTACTCAGAGTTTCTTAATTTGTCCTTTTCATCTCCTCTTAGTGAAGACAAAAATAGTATTGTGTGTTAAATGCGAATCGAAAATACTATGGAGCATTAAAG
ACAAACTGAGGAGTTTAAGTTACTGAAAGAAGAAATTTATTGAAGTTTGTGAAAACGTACACTTCATTTTGGTGAACATAATTGGACCGTTGAGATTCGTATTGGTTTGTTTATTGATTA
TCTAAAGTAGAACTGTAGAAGCATATAAAAAATGCATAACAAAGTGTGTTAGTTATCGTTATAATTAATGTTTTTTTCTCTATGGAGGAGAAAAAAAAATCAAAATATAAATGTAGAAGT
ATTAATTTGTAGGATGCCAGGAAAGGCGTATGCGAGGAGGAAGCAAGTATGGATGAGTGGTGTAAATGAGGTTGACAGTAAAATCTTCTCTAGGGCTATTTCTGCAAAGGTTTATTTCCT
TTTATTCATTCACCACTACACTGTGTATCTACTTCTACTTATTTAGATATACGAAATTTTATATTATCTCATTTCAAACTAATTAATTTTATCTTCTTAATGCTCTTTCTACACTAGAGT
GCCAAAATTCAGGTAAATGTTGCCGTTATTATTTTATTTTCGTAGAAATGAAAGGTATCAGTTAACAGAGTTATTATTTTACATATTTGAGAATTGTTTTGTGATGAAAAAACAAATAAA
AACAGACAGTGGTTTGCATTCCCGTGCTTGATGGCGTTTTGGAAATAGGCACAACGAACAAGGTAATAAAAGTCTATTTATGTCGGTACCAACAAGTGTAAACGAATACATTAGCTCGTT
CTATGTTACATAGATAAATAAAGAAAAATAATGTACAGAACATCATCAACCATTCGCAGTCATATGATTAAAATATAGTTTTTCGATACATTATATACACAACCAAATGATGTAAAAACC
TAATCAAAAATATGCATAAGAGGGAGAACCAAGCCGGAGATGGATGCGTGATATGTTGTGTTTTATGTGAAGGTCAAAGAAAATGAAGAGTTTGTTGAACACATGAAGAGTTTCTTCCAA
AACCACCCGAAGTCAAACACGAAGCCTGCTCTTTTTGAACACTCCATCAACGAAGAGCATGAAGAAGACGAAGAAGTAGAAGAAATGACAATGTCAGAGGAGATAAGACTTGGTTCTCCT
GATGACGATGACGTCTCCAATCAAAATCTACTCTCTGATTTCCATATAGAAGCACCCAGTAGTTTAGGTATGCCGTGCACATTTCTTATTAGATTAAATAGTTAACAAGATCATTTTAAT
TAATTTTCTAATAATAAAATTTTTGAAATGTTTCACATGTCTAGTAATTTTAACATATATCATTGTATATATAGATACACAAATGGACATGATGAATCTAATGGAGGAAGGCGGAAATTA
TTCTCAGACAGTATCAACACTTCTCATGTCACAACTCCCCAATCTTCTTTCAGATTCAGTTTCCACATCTTCTTACGTTCAATCATCGTTTGTCTCGTGGAGGGTTGAGAATGTCAAAGA
GCATCAGCAATATCAACGAGAGGAGAAAGCGTCGTCGTCATCCTCGTCGCAATGGATGCTCAAACACATGATCTTGAGAGTTCCTTTACTCCATGAAAACACTAAAAACAAGAGGGTGCC
GCGGGAAGAGCTCAACCATGTGGTGGCCGAGCGACGCAGAAGAGAGAAGCTTAACGAGAGATTCATAACGTTGAGATCATTGGTTCCATTTGTGACCAAGATGGATAAAGTCTCGATCCT
TGGAGACACCATTGATTACGTAAACCATCTTTGTAAGAGGATCCATGAGCTGGAATCTACTCATCACGAGCCAAACCAAAAGCGGATGCGTATCGGTAAGGGAAGAACGTGGGAAGAGGT
GGAGGTTTCCATTATAGAGAGCGATGTTTTGTTAGAGATGAGATGCGAGTACCGAGATGGTTTATTGCTCAACATTCTTCAGGTACTTAAGGAGCTGGGTATAGAGACCACTGCAGTTCA
CACCGCCGTGAACGACCATGATTTTGAGGCAGAGATAAGGGCGAAAGTGAGAGGGAAGAAACCAACCATTGCTGAGGTTAAAATAGCCATCCATCAAATCATATCTCAAAATAAACTCTA
G

>RsRf_1
CACAGCTAGCCGCATCAATATGTTAGCAACTGAAGAAACATTGATTAGCGGACAATCGGGAAGGAAAATCAAGATCGAGCAACAAGCGTTTGCACTCAAGAATTTTATTTTATTTATTTT
GTTCGGGCACCATGGCCATAAGGATTAAATGATCATCGTCCCACACATGGACAGAATTTGGACGCACCCAGTCAATCCAAGTGGCCGTTAGTATAAAACATAATCCTGAAGCACAAGCCA
AATAACTTTATTTATTGGTCGACAAAAAAGGCCAAGAACGTTTAATGTTGCTTTTGTCTAAACCAGGATACATCTCACATACCACATACCCAACTGTATAAACTATGTGTACGTAACTGT
ATCTATGTGTCTATCAAAATTTTATATGTTTCTCGGGTCAGCTATATCTAACTTTATTGAGTTTTCCAAGACATTACAAGGGAAACAGATGGTGGCTACTTGGCTAGCAATTCACATCAA
TTATTAAAAACTTGAATTGTGAAACATCTCATTCTCCTCCTTTATTACACCAAACTACTTTCTTTTCTCTACTTACCGGTCAGGTCAACAGTTCTACCATTCCCCTTCTTCTTTTTACTC
TTTTCGTTTAAATAAAACTAAAGATGCTACTCTTTTATTATGGTTGATAAACACTGGAATATTGCAAATTTCCAAATAAGAGAAAAGAAAGAGAGATATAAAACCGAAATCATAGTAACA
CAAGTCAAAGCAACGTCTGTTTTCTCTTGGTTGTCAGTTAGCTATTTTCTCTACTGAAATATGTTATGCTTTTTTCCCAATAATATATTATAACATATTAAGAAATATGCATTCGGCATT
TTTGGTTCTCCAATCAGTGGGGGACACGATTACAAGACAATTACATCTTTTTATTTCATCTGTATTCTACTCTCTCATCCAACGTCTGGTTTACCAACCAGTCAGAACGCATAAAAGTCA
ACTTTTACTAAATCTTGAGCTTTTTGGTCATGCATTTATACACATACATAGAATTAATCTAGATTACTTTACTTATGTCAGCATGTTACGAAAACACTAAAATGGTATATATATACATAC
ATATTGAATTAACCGTGGTCCCAAACCAATTTAAATCAGCATAAAGAATATCTTCCTCTTTCATATCAAAACCCCTTCAGAGTTGCAAGATCTTACTCTCAGAGAGCTAATACATGTTTT
CATCTCGGGGAAAAATGGATGAATCAAGTATTATACCGGTATGGAAAGTGATCGGAGCTGAGGAAAAAGAGATTCAAGGGCTACTTAAGGCGGTGGTGCAATCTGTGGGGTGGACTTATA
GTCTCTTCTGGCAACTTTGTCCTCAACGAAGGTTCTCTTTTCATTTCATCCATCTCTCACAATATATAAAGCAATATATTAATTTATCCTTATTAATTATAACAAGTAGAAGTGACTAAT
GTTAAATCGATTAGGAAATTGGTGTGGAGTAGTGGATTCTACAACGGTGCAATAAAGACTAGAAAGACAACTCAGCCGGCGGAAATAACGGCTGAAGAGGCTGCGTTGGAGAGAAGCCAA
CAGCTCATGGAGCTTTACCAGACGCTTTTTGCCGGAGAATCATCGATGGAAGCGAGGGCTTGCACAGCACTGTCGCCTGAGGATTTGACGGACACTGAATGGTTTTATGTGCTGTGTCTC
ACTTACTCTTTTGAACCTCCTTCTGGGTACAACAACTCTTTCTCTCTCTCTCTCTCTCTCTCTCTCTCTCTCTCTCTCTCTCTCTCTCTCTCTCTCTCTCTCTCTCTCTCCAAAGTTTTT
TCTTTCTTTCTATAAAGACTACTCAGAGTTTCTTAATTTGTCCTTTTCATCTCCTCTTAGTGAAGACAAAAATAGTATTGTGTGTTAAATGCGAATCGAAAATACTATGGAGCATTAAAG
ACAAACTGAGGAGTTTAAGTTACTGAAAGAAGAAATTTATTGAAGTTTGTGAAAACGTACACTTCATTTTGGTGAACATAATTGGACCGTTGAGATTCGTATTGGTTTGTTTATTGATTA
TCTAAAGTAGAACTGTAGAAGCATATAAAAAATGCATAACAAAGTGTGTTAGTTATCGTTATAATTAATGTTTTTTTCTCTATGGAGGAGAAAAAAAAATCAAAATATAAATGTAGAAGT
ATTAATTTGTAGGATGCCAGGAAAGGCGTATGCGAGGAGGAAGCAAGTATGGATGAGTGGTGTAAATGAGGTTGACAGTAAAATCTTCTCTAGGGCTATTTCTGCAAAGGTTTATTTCCT
TTTATTCATTCACCACTACACTGTGTATCTACTTCTACTTATTTAGATATACGAAATTTTATATTATCTCATTTCAAACTAATTAATTTTATCTTCTTAATGCTCTTTCTACACTAGAGT
GCCAAAATTCAGGTAAATGTTGCCGTTATTATTTTATTTTCGTAGAAATGAAAGGTATCAGTTAACAGAGTTATTATTTTACATATTTGAGAATTGTTTTGTGATGAAAAAACAAATAAA
AACAGACAGTGGTTTGCATTCCCGTGCTTGATGGCGTTTTGGAAATAGGCACAACGAACAAGGTAATAAAAGTCTATTTATGTCGGTACCAACAAGTGTAAACGAATACATTAGCTCGTT

CTATGTTACATAGATAAATAAAGAAAAATAATGTACAGAACATCATCAACCATTCGCAGTCATATGATTAAAATATAGTTTTTCGATACATTATATACACAACCAAATGATGTAAAAACC
TAATCAAAAATATGCATAAGAGGGAGAACCAAGCCGGAGATGGATGCGTGATATGTTGTGTTTTATGTGAAGGTCAAAGAAAATGAAGAGTTTGTTGAACACATGAAGAGTTTCTTCCAA
AACCACCCGAAGTCAAACACGAAGCCTGCTCTTTTTGAACACTCCATCAACGAAGAGCATGAAGAAGACGAAGAAGTAGAAGAAATGACAATGTCAGAGGAGATAAGACTTGGTTCTCCT
GATGACGATGACGTCTCCAATCAAAATCTACTCTCTGATTTCCATATAGAAGCACCCAGTAGTTTAGGTATGCCGTGCACATTTCTTATTAGATTAAATAGTTAACAAGATCATTTTAAT
TAATTTTCTAATAATAAAATTTTTGAAATGTTTCACATGTCTAGTAATTTTAACATATATCATTGTATATATAGATACACAAATGGACATGATGAATCTAATGGAGGAAGGCGGAAATTA
TTCTCAGACAGTATCAACACTTCTCATGTCACAACTCCCCAATCTTCTTTCAGATTCAGTTTCCACATCTTCTTACGTTCAATCATCGTTTGTCTCGTGGAGGGTTGAGAATGTCAAAGA
GCATCAGCAATATCAACGAGAGGAGAAAGCGTCGTCGTCATCCTCGTCGCAATGGATGCTCAAACACATGATCTTGAGAGTTCCTTTACTCCATGAAAACACTAAAAACAAGAGGGTGCC
GCGGGAAGAGCTCAACCATGTGGTGGCCGAGCGACGCAGAAGAGAGAAGCTTAACGAGAGATTCATAACGTTGAGATCATTGGTTCCATTTGTGACCAAGATGGATAAAGTCTCGATCCT
TGGAGACACCATTGATTACGTAAACCATCTTTGTAAGAGGATCCATGAGCTGGAATCTACTCATCACGAGCCAAACCAAAAGCGGATGCGTATCGGTAAGGGAAGAACGTGGGAAGAGGT
GGAGGTTTCCATTATAGAGAGCGATGTTTTGTTAGAGATGAGATGCGAGTACCGAGATGGTTTATTGCTCAACATTCTTCAGGTACTTAAGGAGCTGGGTATAGAGACCACTGCAGTTCA
CACCGCCGTGAACGACCATGATTTTGAGGCAGAGATAAGGGCGAAAGTGAGAGGGAAGAAACCAACCATTGCTGAGGTTAAAATAGCCATCCATCAAATCATATCTCAAAATAAACTCTA
G

>RsRf_2
CACAGCTAGCCGCATCAATATGTTAGCAACTGAAGAAACATTGATTAGCGGACAATCGGGAAGGAAAATCAAGATCGAGCAACAAGCGTTTGCACTCAAGAATTTTATTTTATTTATTTT
GTTCGGGCACCATGGCCATAAGGATTAAATGATCATCGTCCCACACATGGACAGAATTTGGACGCACCCAGTCAATCCAAGTGGCCGTTAGTATAAAACATAATCCTGAAGCACAAGCCA
AATAACTTTATTTATTGGTCGACAAAAAAGGCCAAGAACGTTTAATGTTGCTTTTGTCTAAACCAGGATACATCTCACATACCACATACCCAACTGTATAAACTATGTGTACGTAACTGT
ATCTATGTGTCTATCAAAATTTTATATGTTTCTCGGGTCAGCTATATCTAACTTTATTGAGTTTTCCAAGACATTACAAGGGAAACAGATGGTGGCTACTTGGCTAGCAATTCACATCAA
TTATTAAAAACTTGAATTGTGAAACATCTCATTCTCCTCCTTTATTACACCAAACTACTTTCTTTTCTCTACTTACCGGTCAGGTCAACAGTTCTACCATTCCCCTTCTTCTTTTTACTC
TTTTCGTTTAAATAAAACTAAAGATGCTACTCTTTTATTATGGTTGATAAACACTGGAATATTGCAAATTTCCAAATAAGAGAAAAGAAAGAGAGATATAAAACCGAAATCATAGTAACA
CAAGTCAAAGCAACGTCTGTTTTCTCTTGTTTGTCAGTTAGCTATTTTCTCTACTGAAATATGTTATGCTTTTTTCCCAATAATATATTATAACATATTAAGAAATATGCATTCGGCATT
TTTGGTTCTCCAATCAGTGGGGGACACGATTACAAGACAATTACATCTTTTTATTTCATCTGTATTCTACTCTCTCATCCAACGTCTGGTTTACCAACCAGTCAGAACGCATAAAAGTCA
ACTTTTACTAAATCTTGAGCTTTTTGGTCATGCATTTATACACATACATAGAATTAATCTAGATTACTTTACTTATGTCAGCATGTTACGAAAACACTAAAATGGTATATATATACATAC
ATATTGAATTAACCGTGGTCCCAAACCAATTTAAATCAGCATAAAGAATATCTTCCTCTTTCATATCAAAACCCCTTCAGAGTTGCAAGATCTTACTCTCAGAGAGCTAATACATGTTTT
CATCTCGGGGAAAAAATGGATGAATCAAGTATTATACCGGTATGGAAAGTGATCGGAGCTGAGGAAAAAGAGATTCAAGGGCTACTTAAGGCGGTGGTGCAATCTGTGGGGTGGACTTATA
GTCTCTTCTGGCAACTTTGTCCTCAACGAAGGTTCTCTTTTCATTTCATCCATCTCTCACAATATATAAAGCAATATATTAATTTATCCTTATTAATTATAACAAGTAGAAGTGACTAAT
GTTAAATCGATTAGGAAATTGGTGTGGAGTAGTGGATTCTACAACGGTGCAATAAAGACTAGAAAGACAACTCAGCCGGCGGAAATAACGGCTGAAGAGGCTGCGTTGGAGAGAAGCCAA
CAGCTCATGGAGCTTTACCAGACGCTTTTTGCCGGAGAATCATCGATGGAAGCGAGGGCTTGCACAGCACTGTCGCCTGAGGATTTGACGGACACTGAATGGTTTTATGTGCTGTGTCTC
ACTTACTCTTTTGAACCTCCTTCTGGGTACAACAACTCTTTCTCTCTCTCTCTCTCTCTCTCTCTCTCTCTCTCTCTCTCTCTCTCTCTCTCTCTCTCTCTCTCTCTCTCTCTCTCCAAA
GTTTTTTCTTTCTTTCTATAAAGACTACTCAGAGTTTCTTAATTTGTCCTTTTCATCTCCTCTTAGTGAAGACAAAAATAGTATTGTGTGTTAAATGCGAATCGAAAATACTATGGAGCA
TTAAAGACAAACTGAGGAGTTTAAGTTACTGAAAGAAGAAATTTATTGAAGTTTGTGAAAACGTACACTTCATTTTGGTGAACATAATTGGACCGTTGAGATTCGTATTGGTTTGTTTAT
TGATTATCTAAAGTAGAACTGTAGAAGCATATAAAAAATGCATAACAAAGTGTGTTAGTTATCGTTATAATTAATGTTTTTTTCTCTATGGAGGAGAAAAAAAAATCAAAATATAAATGT
AGAAGTATTAATTTGTAGGATGCCAGGAAAGGCGTATGCGAGGAGGAAGCAAGTATGGATGAGTGGTGTAAATGAGGTTGACAGTAAAATCTTCTCTAGGGCTATTTCTGCAAAGGTTTA
TTTCCTTTTATTCATTCACCACTACACTGTGTATCTACTTCTACTTATTTAGATATACGAAATTTTATATTATCTCATTTCAAACTAATTAATTTTATCTTCTTAATGCTCTTTCTACAC
TAGAGTGCCAAAATTCAGGTAAATGTTGCCGTTATTATTTTATTTTCGTAGAAATGAAAGGTATCAGTTAACAGAGTTATTATTTTACATATTTGAGAATTGTTTTGTGATGAAAAAACA
AATAAAAACAGACAGTGGTTTGCATTCCCGTGCTTGATGGCGTTTTGGAAATAGGCACAACGAACAAGGTAATAAAAGTCTATTTATGTCGGTACCAACAAGTGTAAACGAATACATTAG
CTCGTTCTATGTTACATAGATAAATAAAGAAAAATAATGTACAGAACATCATCAACCATTCGCAGTCATATGATTAAAATATAGTTTTTCGATACATTATATACACAACCAAATGATGTA
AAAACCTAATCAAAAATATGCATAAGAGGGAGAACCAAGCCGGAGATGGATGCGTGATATGTTGTGTTTTATGTGAAGGTCAAAGAAAATGAAGAGTTTGTTGAACACATGAAGAGTTTC
TTCCAAAACCACCCGAAGTCAAACACGAAGCCTGCTCTTTTTGAACACTCCATCAACGAAGAGCATGAAGAAGACGAAGAAGTAGAAGAAATGACAATGTCAGAGGAGATAAGACTTGGT
TCTCCTGATGACGATGACGTCTCCAATCAAAATCTACTCTCTGATTTCCATATAGAAGCACCCAGTAGTTTAGGTATGCCGTGCACATTTCTTATTAGATTAAATAGTTAACAAGATCAT
TTTAATTAATTTTCTAATAATAAAATTTTTGAAATGTTTCACATGTCTAGTAATTTTAACATATATCATTGTATATATAGATACACAAATGGACATGATGAATCTAATGGAGGAAGGCGG
AAATTATTCTCAGACAGTATCAACACTTCTCATGTCACAACTCCCCAATCTTCTTTCAGATTCAGTTTCCACATCTTCTTACGTTCAATCATCGTTTGTCTCGTGGAGGGTTGAGAATGT
CAAAGAGCATCAGCAATATCAACGAGAGGAGAAAGCGTCGTCGTCATCCTCGTCGCAATGGATGCTCAAACACATGATCTTGAGAGTTCCTTTACTCCATGAAAACACTAAAAACAAGAG
GGTGCCGCGGGAAGAGCTCAACCATGTGGTGGCCGAGCGACGCAGAAGAGAGAAGCTTAACGAGAGATTCATAACGTTGAGATCATTGGTTCCATTTGTGACCAAGATGGATAAAGTCTC
GATCCTTGGAGACACCATTGATTACGTAAACCATCTTTGTAAGAGGATCCATGAGCTGGAATCTACTCATCACGAGCCAAACCAAAAGCGGATGCGTATCGGTAAGGGAAGAACGTGGGA
AGAGGTGGAGGTTTCCATTATAGAGAGCGATGTTTTGTTAGAGATGAGATGCGAGTACCGAGATGGTTTATTGCTCAACATTCTTCAGGTACTTAAGGAGCTGGGTATAGAGACCACTGC
AGTTCACACCGCCGTGAACGACCATGATTTTGAGGCAGAGATAAGGGCGAAAGTGAGAGGGAAGAAACCAACCATTGCTGAGGTTAAAATAGCCATCCATCAAATCATATCTCAAAATAA
ACTCTAG

>WsWf_1
CACAGCTAGCCGCATCAATATGTTAGCAACTGAAGAAACATTGATTAGCGGACAATCGGGAAGGAAAATCAAGATCGAGCAACAAGCGTTTGCACTCAAGAATTTTTTTTTTTTTTGTTC
GGGCACCATGGCCATAAGGATTAAATGATCATCGTCCCACACATGGACAGAATTTGGACGCACCCAGTCAATCCAAGTGGCCGTTAGTATAAAACATAATCCTGAAGCACAAGCCAAATA
ACTTTATTTATTGGTCGACAAAAAGGCCAAGAAACGTTTAATGTTGCTTTTGTCTAAACCAGGATACATCTCACATACCACATACCCAACTGTATAAACTATATGTGTACGTAACTGTAT
CTATGTGTCTATCAAATTTTTATATGTTTCTGGGGTCAGCTATGTCTAACTTTATTGAGTTTTCCAAGACATTACAAGGGAAACAGATGGTGGCTACTTGGCTAGCAATTCACATCAATT
ACTATTAAAAACTTGAATTGTGAAACATCTCATTCTCCTCCTTTATTACACCAAACTACTTTCTTTTCTCTACTTACCGGTCAGGTCAACAGTTCTACCATTCCCCTTCTTCTTTTTACT
CTTTTCGTTTAAATAAAACTAAAGATGCTACTCTTTTATTATGGTTGATAAACACTGGAATATTGCAAATTTCCAAATAAGAGAAAAGAAAGAGAGATGTAAAACCGAAATCATAGTAAC
ACAAGTCAAAGCAACGTCTGTTTTCTCTTGTTTGTCAGTTAGCTATTTTCTCTACTAAAATATGTTATGCTTTTTTCCCAATAATATTATAACATATTAATGCATTCGGCATTTTTGGTT
CTCCAATCCGTGGGGGACACGATTACAAGACCATTTCATCTTTTTATTTCATCTGTATTCTACTCTCTCATCCGACGTCTGGTTTACCAACCAGTCAGAACGCATAAAAGCCAACTTTTA
CTAAATCTTGAGTTTTTGGTCATGCATTTATACACATACATAGAATTAATCTAGATTACTTTACTTATGTCAGCATGTTACGAAAACACTAAAATGGTATATATATGCATACATATTGAA
TTAACGTGGTCCCAAACCAATTTAAATCAGCATAAAGAATATCTTCCTCTTTCATATCAAAACCCCTTCAGAGTTGCAAGATCTTACTCTCTTCAGAGTTGCGAGAGAGAGCTAATATAT
GTTTTCATCTCGGGGAAAAAATGGATGAATCAAGTATTATACCGGTATGGAAAGTGATCGGAGCTGAGGAAAAAGAGATTCAAGGGCTACTTAAGGCGGTGGTGCAATCTGTGGGGTGGAC
TTATAGTCTCTTCTGGCAACTTTGTCCTCAACGAAGGTTCTCTTTTCATTTCATCCATCTCTCACAATATATAAAGCAATATATTAATTTATCCTTATTAATTATAACCTCAATTATAAC
AAGTAGAAGTGACTAAATGTTAAATCGATTAGGAAATTGGTGTGGAGTAGTGGATTCTACAACGGTGCAATAAAGACTAGAAAGACAACTCAGCCGGCGGAAATAACGGCTGAAGAGGCT
GCGTTGGAGAGAAGCCAACAGCTCATGGAGCTTTACCAGACGCTTTTTGCCGGAGAATCATCGATGGAAGCGAGGGCTTGCACAGCACTGTCGCCTGAGGATTTGACGGACACTGAATGG
TTTTATGTGCTGTGTCTCACTTACTCTTTTGAACCTCCTTCTGGGTACAACAACTCTTTCTCTCTCTCTCTCTCTCTCTCTCTCTCTCTCTCTCTCTCTCTCTCTCTCTCTCTCTCTCTC
TCTCTCTCTCTCTCTCTCTCTCTCTCTCTCTCTCTCTCTCTCTCTCTCTCTCTCTCTCTCTCTCTCTCTCTCTCCAAAGTTTTTTCTTTCTTTCTATAAAGGCTACTCAGAGTTTCTT
AATTTGTCCTTTTCATCTCCTCTTAGTGAAGACAAAAATAGTATTGTGTGTTAAATGCGAATCGAAAATACTATGGAGCATTAAAGACAAACTGAGGAGTTTAAGTTACTGAAAGAAGAA
ATTTATTGAAGTTTGTGAAAACGTACACTTCATTTTGGTGAACATAATTGGACCGTTGAGATTCGTATTGGTTTGTTTATTGATTATCTAAAGTAGAACTGTAGAAGCATATAAAAAATG
CATAACAAAGTGTGTTAGTTATCGTTATAATTAATGTTTTTTTCTCTATGGAGGAGAAAAAAAAATCAAAATATAAATGTAGAAGTATTAATTTGTAGGATGCCAGGAAAGGCGTATGCG
AGGAGGAAGCAAGTATGGATGAGTGGTGTAAATGAGGTTGACAGTAAAATCTTCTCTAGGGCTATTTCTGCAAAGGTTTATTTCCTTTTATTCATTCACCACTACACGGTGTATCTACTT
CTACTTATTTAGATATACGAAATTTTATATTATCTCATTTCAAACTAATTAATTTTATCTTCTTAATGCTCTTTCTACACTAGAGTGCCAAAATTCAGGTAAATGTTGCCGTTATTATTT
TATTTTCGTAGAAATGAAAGGTATCAGTTAACAGAGTTATTATTTTACATATTTGAGAATTGTTTTGTGATGAAAAAACAAATAAAAACAGACAGTGGTTTGCATTCCCGTGCTTGATGG
CGTTTTGGAAATAGGCACAACGAACAAGGTAATAAAAGTCTATTATGTCGGTACCAACAAGTGTAAACGAATACATTAGCTCGTTCTATTACATAGATAAATAAAGAAAAATAATGTACA
GAACATCAACCATTCGCAGTCATATGATTAAAATATAGTTTTTCGATACATTATATACACAACCAAATGATGTAAAAACCTAATCAAAAATATGCATAAGAGGGAGAACAAAGCCGGAGA
TGGATGCGTGATATGTTGTGTTTTATGTGAAGGTCAAAGAAAATGAAGAGTTTGTTGAACACATAAAGAGTTTCTTCCAAAACCACCCGAAGTCAAACACGAAGCCTGCTCTTTTTGAAC
ACTCCATCAACGAAGAGCATGAAGAAGACGAAGAAGTAGAAGAAATGACAATGTCAGAGGAGATAAGACTTGGTTCTCCTGATGACGATGACGTCTCCAATCAAAATCTACTCTCTGATT
TCCATATAGAAGCACCCAATAGTTTAGGTATGCCGTACACCTTTCTTATTAGATTAAAATAGTTAACAAGATCATTTTAACTAATTTTCTAATAAATTTTTTTTTGAAATGTTTCACATG
TCTGGTAATTTTACATATATCATTGTATATATAGATACACAAATGGACATGATGAATCTAATGGAGGAAGGCGGAAGTTATTCTCAGACAGTATCAACACTTCTCATGTCACAACTCCCC
AATCTTCTTTCAGATTCAGTTTCCACATCTTCTTACGTTCAATCATCGTTTGTCTCGTGGAGGGTTGAGAATGTCAAAGAGCATCAGCAATATCAACGAGAGGAGAAAGCGTCGTCGTCA
TCCTCGTCGCAATGGATGCTCAAACACATGATCTTGAGAGTTCCTTTACTCCATGAAAACACTAAAAACAAGAGGGTGCCGCGGGAAGAGCTCAACCATGTGGTGGCCGAGCGACGCAGA
AGAGAGAAGCTTAACGAGAGATTCATAACGTTGAGATCATTGGTTCCATTTGTGACCAAGATGGATAAAGTCTCGATCCTTGGAGACACCATTGATTACGTAAACCATCTTTGTAAGAGG
ATCCATGAGCTGGAATCTACTCATCACGAGCCAAACCAAAAGCGGATGCGTATCGGTAAGGGAAGAACGTGGGAAGAGGTGGAGGTTTCCATTATAGAGAGCGATGTTTTGTTAGAGATG
AGATGCGAGTACCGAGATGGTTTATTGCTCAACATTCTTCAGGTACTTAAGGAGCTGGGTATAGAGACCACTGCAGTTCACACCGCCGTGAACGACCATGATTTTGAGGCAGAGATAAGG
GCGAAAGTGAGAGGGAAGAAACCAACCATTGCTGAGGTTAAAATAGCCATCCATCAAATCATATCTCAAAATAAACTCTAG

>WsWf_2
CACAGCTAGCCGCATCAATATGTTAGCAACTGAAGAAACATTGATTAGCGGACAATCGGGAAGGAAAATCAAGATCGAGCAACAAGCGTTTGCACTCAAGAATTTTTTTTTTTTTTGTTC
GGGCACCATGGCCATAAGGATTAAATGATCATCGTCCCACACATGGACAGAATTTGGACGCACCCAGTCAATCCAAGTGGCCGTTAGTATAAAACATAATCCTGAAGCACAAGCCAAATA
ACTTTATTTATTGGTCGACAAAAAGGCCAAGAAACGTTTAATGTTGCTTTTGTCTAAACCAGGATACATCTCACATACCACATACCCAACTGTATAAACTATATGTGTACGTAACTGTAT
CTATGTGTCTATCAAATTTTTATATGTTTCTGGGGTCAGCTATGTCTAACTTTATTGAGTTTTCCAAGACATTACAAGGGAAACAGATGGTGGCTACTTGGCTAGCAATTCACATCAATT
ACTATTAAAAACTTGAATTGTGAAACATCTCATTCTCCTCCTTTATTACACCAAACTACTTTCTTTTCTCTACTTACCGGTCAGGTCAACAGTTCTACCATTCCCCTTCTTCTTTTTACT
CTTTTCGTTTAAATAAAACTAAAGATGCTACTCTTTTATTATGGTTGATAAACACTGGAATATTGCAAATTTCCAAATAAGAGAAAAGAAAGAGAGATGTAAAACCGAAATCATAGTAAC
ACAAGTCAAAGCAACGTCTGTTTTCTCTTGTTTGTCAGTTAGCTATTTTCTCTACTAAAATATGTTATGCTTTTTTCCCAATAATATTATAACATATTAATGCATTCGGCATTTTTGGTT
CTCCAATCCGTGGGGGACACGATTACAAGACCATTTCATCTTTTTATTTCATCTGTATTCTACTCTCTCATCCGACGTCTGGTTTACCAACCAGTCAGAACGCATAAAAGCCAACTTTTA
CTAAATCTTGAGTTTTTGGTCATGCATTTATACACATACATAGAATTAATCTAGATTACTTTACTTATGTCAGCATGTTACGAAAACACTAAAATGGTATATATATGCATACATATTGAA
TTAACGTGGTCCCAAACCAATTTAAATCAGCATAAAGAATATCTTCCTCTTTCATATCAAAACCCCTTCAGAGTTGCAAGATCTTACTCTCTTCAGAGTTGCGAGAGAGAGCTAATATAT
GTTTTCATCTCGGGGAAAAATGGATGAATCAAGTATTATACCGGTATGGAAAGTGATCGGAGCTGAGGAAAAAGAGATTCAAGGGCTACTTAAGGCGGTGGTGCAATCTGTGGGGTGGAC
TTATAGTCTCTTCTGGCAACTTTGTCCTCAACGAAGGTTCTCTTTTCATTTCATCCATCTCTCACAATATATAAAGCAATATATTAATTTATCCTTATTAATTATAACCTCAATTATAAC
AAGTAGAAGTGACTAAATGTTAAATCGATTAGGAAATTGGTGTGGAGTAGTGGATTCTACAACGGTGCAATAAAGACTAGAAAGACAACTCAGCCGGCGGAAATAACGGCTGAAGAGGCT
GCGTTGGAGAGAAGCCAACAGCTCATGGAGCTTTACCAGACGCTTTTTGCCGGAGAATCATCGATGGAAGCGAGGGCTTGCACAGCACTGTCGCCTGAGGATTTGACGGACACTGAATGG
TTTTATGTGCTGTGTCTCACTTACTCTTTTGAACCTCCTTCTGGGTACAACAACTCTTTCTCTCTCTCTCTCTCTCTCTCTCTCTCTCTCTCTCTCTCTCTCTCTCTCTCTCTCTCTCTC
TCTCTCTCTCTCTCTCTCTCTCTCTCTCTCTCTCTCTCTCTCTCTCTCTCTCTCTCTCTCTCTCTCTCTCCAAAGTTTTTTCTTTCTTTCTATAAAGGCTACTCAGAGTTTCTTAA
TTTGTCCTTTTCATCTCCTCTTAGTGAAGACAAAAATAGTATTGTGTGTTAAATGCGAATCGAAAATACTATGGAGCATTAAAGACAAACTGAGGAGTTTAAGTTACTGAAAGAAGAAAT
TTATTGAAGTTTGTGAAAACGTACACTTCATTTTGGTGAACATAATTGGACCGTTGAGATTCGTATTGGTTTGTTTATTGATTATCTAAAGTAGAACTGTAGAAGCATATAAAAAATGCA
TAACAAAGTGTGTTAGTTATCGTTATAATTAATGTTTTTTTCTCTATGGAGGAGAAAAAAAAATCAAAATATAAATGTAGAAGTATTAATTTGTAGGATGCCAGGAAAGGCGTATGCGAG
GAGGAAGCAAGTATGGATGAGTGGTGTAAATGAGGTTGACAGTAAAATCTTCTCTAGGGCTATTTCTGCAAAGGTTTATTTCCTTTTATTCATTCACCACTACACGGTGTATCTACTTCT
ACTTATTTAGATATACGAAATTTTATATTATCTCATTTCAAACTAATTAATTTTATCTTCTTAATGCTCTTTCTACACTAGAGTGCCAAAATTCAGGTAAATGTTGCCGTTATTATTTTA
TTTTCGTAGAAATGAAAGGTATCAGTTAACAGAGTTATTATTTTACATATTTGAGAATTGTTTTGTGATGAAAAAACAAATAAAAACAGACAGTGGTTTGCATTCCCGTGCTTGATGGCG
TTTTGGAAATAGGCACAACGAACAAGGTAATAAAAGTCTATTATGTCGGTACCAACAAGTGTAAACGAATACATTAGCTCGTTCTATTACATAGATAAATAAAGAAAAATAATGTACAGA
ACATCAACCATTCGCAGTCATATGATTAAAATATAGTTTTTCGATACATTATATACACAACCAAATGATGTAAAAACCTAATCAAAAATATGCATAAGAGGGAGAACAAAGCCGGAGATG
GATGCGTGATATGTTGTGTTTTATGTGAAGGTCAAAGAAAATGAAGAGTTTGTTGAACACATAAAGAGTTTCTTCCAAAACCACCCGAAGTCAAACACGAAGCCTGCTCTTTTTGAACAC
TCCATCAACGAAGAGCATGAAGAAGACGAAGAAGTAGAAGAAATGACAATGTCAGAGGAGATAAGACTTGGTTCTCCTGATGACGATGACGTCTCCAATCAAAATCTACTCTCTGATTTC
CATATAGAAGCACCCAATAGTTTAGGTATGCCGTACACCTTTCTTATTAGATTAAAATAGTTAACAAGATCATTTTAACTAATTTTCTAATAAATTTTTTTTTGAAATGTTTCACATGTC
TGGTAATTTTACATATATCATTGTATATATAGATACACAAATGGACATGATGAATCTAATGGAGGAAGGCGGAAGTTATTCTCAGACAGTATCAACACTTCTCATGTCACAACTCCCCAA
TCTTCTTTCAGATTCAGTTTCCACATCTTCTTACGTTCAATCATCGTTTGTCTCGTGGAGGGTTGAGAATGTCAAAGAGCATCAGCAATATCAACGAGAGGAGAAAGCGTCGTCGTCATC
CTCGTCGCAATGGATGCTCAAACACATGATCTTGAGAGTTCCTTTACTCCATGAAAACACTAAAAACAAGAGGGTGCCGCGGGAAGAGCTCAACCATGTGGTGGCCGAGCGACGCAGAAG
AGAGAAGCTTAACGAGAGATTCATAACGTTGAGATCATTGGTTCCATTTGTGACCAAGATGGATAAAGTCTCGATCCTTGGAGACACCATTGATTACGTAAACCATCTTTGTAAGAGGAT
CCATGAGCTGGAATCTACTCATCACGAGCCAAACCAAAAGCGGATGCGTATCGGTAAGGGAAGAACGTGGGAAGAGGTGGAGGTTTCCATTATAGAGAGCGATGTTTTGTTAGAGATGAG
ATGCGAGTACCGAGATGGTTTATTGCTCAACATTCTTCAGGTACTTAAGGAGCTGGGTATAGAGACCACTGCAGTTCACACCGCCGTGAACGACCATGATTTTGAGGCAGAGATAAGGGC
GAAAGTGAGAGGGAAGAAACCAACCATTGCTGAGGTTAAAATAGCCATCCATCAAATCATATCTCAAAATAAACTCTAG

Sequence comparison

CLUSTAL 2.1 Multiple Sequence Alignments

```
Sequence type explicitly set to DNA
Sequence format is Pearson
Sequence 1: RsWf_1      3934 bp
Sequence 2: RsWf_2      3934 bp
Sequence 3: GsRf_1      3965 bp
Sequence 4: GsRf_2      3961 bp
Sequence 5: RsRf_1      3961 bp
Sequence 6: RsRf_2      3967 bp
Sequence 7: WsWf_1      4041 bp
Sequence 8: WsWf_2      4039 bp
Start of Pairwise alignments
```

```
                                                                                           -1233
                                                                                  GsRf_1   CACAGCTAGCCGCATCAATATGTTAGCAACTGA
                                                                                  RsRf_2   CACAGCTAGCCGCATCAATATGTTAGCAACTGA
                                                                                  RsRf_1   CACAGCTAGCCGCATCAATATGTTAGCAACTGA
                                                                                  GsRf_2   CACAGCTAGCCGCATCAATATGTTAGCAACTGA
                                                                                  RsWf_1   CACAGCTAGCCGCATCAATATGTTAGCAACTGA
                                                                                  RsWf_2   CACAGCTAGCCGCATCAATATGTTAGCAACTGA
                                                                                  WsWf_1   CACAGCTAGCCGCATCAATATGTTAGCAACTGA
                                                                                  WsWf_2   CACAGCTAGCCGCATCAATATGTTAGCAACTGA
                                                                                           *********************************

         -1200
GsRf_1   AGAAACATTGATTAGCGGACAATCGGGAAGGAAAATCAAGATCGAGCAACAAGCGTTTGCACTCAAGAATTTTATTTTATTTATTTTGTTCGGGCACCATGGCCATAAGGATTAAATGAT
RsRf_2   AGAAACATTGATTAGCGGACAATCGGGAAGGAAAATCAAGATCGAGCAACAAGCGTTTGCACTCAAGAATTTTATTTTATTTATTTTGTTCGGGCACCATGGCCATAAGGATTAAATGAT
RsRf_1   AGAAACATTGATTAGCGGACAATCGGGAAGGAAAATCAAGATCGAGCAACAAGCGTTTGCACTCAAGAATTTTATTTTATTTATTTTGTTCGGGCACCATGGCCATAAGGATTAAATGAT
GsRf_2   AGAAACATTGATTAGCGGACAATCGGGAAGGAAAATCAAGATCGAGCAACAAGCGTTTGCACTCAAGAATTTTATTTTATTTATTTTGTTCGGGCACCATGGCCATAAGGATTAAATGAT
RsWf_1   AGAAACATTGATTAGCGGACAATCGGGAAGGAAAATCAAGATCGAGCAACAAGCGTTTGCACTCAAGAATTTTATTTTATTTTTTTTGTTCGGGCACCATGGCCATAAGGATTAAATGAT
RsWf_2   AGAAACATTGATTAGCGGACAATCGGGAAGGAAAATCAAGATCGAGCAACAAGCGTTTGCACTCAAGAATTTTATTTTATTTTTTTTGTTCGGGCACCATGGCCATAAGGATTAAATGAT
WsWf_1   AGAAACATTGATTAGCGGACAATCGGGAAGGAAAATCAAGATCGAGCAACAAGCGTTTGCACTCAAGAATTTTTTTT----TTTTTTGTTCGGGCACCATGGCCATAAGGATTAAATGAT
WsWf_2   AGAAACATTGATTAGCGGACAATCGGGAAGGAAAATCAAGATCGAGCAACAAGCGTTTGCACTCAAGAATTTTTTTT----TTTTTTGTTCGGGCACCATGGCCATAAGGATTAAATGAT
         ************************************************************************* ***    * *************************************

         -1080                                                                                      Rf-PF: 5′-TTGGTCGACAAAAAAGGCCAAGA
GsRf_1   CATCGTCCCACACATGGACAGAATTTGGACGCACCCAGTCAATCCAAGTGGCCGTTAGTATAAAACATAATCCTGAAGCACAAGCCAAATAACTTTATTTATTGGTCGACAAAAAAGGCC
RsRf_2   CATCGTCCCACACATGGACAGAATTTGGACGCACCCAGTCAATCCAAGTGGCCGTTAGTATAAAACATAATCCTGAAGCACAAGCCAAATAACTTTATTTATTGGTCGACAAAAAAGGCC
RsRf_1   CATCGTCCCACACATGGACAGAATTTGGACGCACCCAGTCAATCCAAGTGGCCGTTAGTATAAAACATAATCCTGAAGCACAAGCCAAATAACTTTATTTATTGGTCGACAAAAAAGGCC
GsRf_2   CATCGTCCCACACATGGACAGAATTTGGACGCACCCAGTCAATCCAAGTGGCCGTTAGTATAAAACATAATCCTGAAGCACAAGCCAAATAACTTTATTTATTGGTCGACAAAAAAGGCC
RsWf_1   CATCGTCCCACACATGGACAGAATTTGGACGCACCCAGTCAATCCAAGTGGCCGTTAGTATAAAACATAATCCTGAAGCACAAGCCAAATAACTTTATTTATTGGTCGACAAAACCGCCA
RsWf_2   CATCGTCCCACACATGGACAGAATTTGGACGCACCCAGTCAATCCAAGTGGCCGTTAGTATAAAACATAATCCTGAAGCACAAGCCAAATAACTTTATTTATTGGTCGACAAAACCGCCA
WsWf_1   CATCGTCCCACACATGGACAGAATTTGGACGCACCCAGTCAATCCAAGTGGCCGTTAGTATAAAACATAATCCTGAAGCACAAGCCAAATAACTTTATTTATTGGTCGACAAAAAGGCCA
WsWf_2   CATCGTCCCACACATGGACAGAATTTGGACGCACCCAGTCAATCCAAGTGGCCGTTAGTATAAAACATAATCCTGAAGCACAAGCCAAATAACTTTATTTATTGGTCGACAAAAAGGCCA
         ******************************************************************************************************************* * *
                                                                                               Wf-PF: 5′-GGTCGACAAAACCGCCAAGAA

         -960
GsRf_1   AAGAACGTTTAATGTTGCTTTTGTCTAAACCAGGATACATCTCACATACCACATACCCAACTGTATAAACTAT--GTGTACGTAACTGTATCTATGTGTCTATCAAAATTTTATATGTTT
RsRf_2   AAGAACGTTTAATGTTGCTTTTGTCTAAACCAGGATACATCTCACATACCACATACCCAACTGTATAAACTAT--GTGTACGTAACTGTATCTATGTGTCTATCAAAATTTTATATGTTT
RsRf_1   AAGAACGTTTAATGTTGCTTTTGTCTAAACCAGGATACATCTCACATACCACATACCCAACTGTATAAACTAT--GTGTACGTAACTGTATCTATGTGTCTATCAAAATTTTATATGTTT
GsRf_2   AAGAACGTTTAATGTTGCTTTTGTCTAAACCAGGATACATCTCACATACCACATACCCAACTGTATAAACTAT--GTGTACGTAACTGTATCTATGTGTCTATCAAAATTTTATATGTTT
RsWf_1   AGAAACGTTTAATGTTGCTTTTGTCTAAACCAGAATACATCTCACATACCACATACCCAACTGTATAAACTATATGTGTACGTAACTGTATCTATGTGTCTATCAAATTTTTATATGTTT
RsWf_2   AGAAACGTTTAATGTTGCTTTTGTCTAAACCAGAATACATCTCACATACCACATACCCAACTGTATAAACTATATGTGTACGTAACTGTATCTATGTGTCTATCAAATTTTTATATGTTT
WsWf_1   AGAAACGTTTAATGTTGCTTTTGTCTAAACCAGGATACATCTCACATACCACATACCCAACTGTATAAACTATATGTGTACGTAACTGTATCTATGTGTCTATCAAATTTTTATATGTTT
WsWf_2   AGAAACGTTTAATGTTGCTTTTGTCTAAACCAGGATACATCTCACATACCACATACCCAACTGTATAAACTATATGTGTACGTAACTGTATCTATGTGTCTATCAAATTTTTATATGTTT
         *  ****************************** ***************************************  ******************************** ************

Rf-PR: 5′-CCCAGTTAGATATAGCTGACCCGA
         -840
GsRf_1   CTCGGGTCAGCTATATCTAACTTTATTGAGTTTTCCAAGACATTACAAGGGAAACAGATGGTGGCTACTTGGCTAGCAATTCACATCAATTA---TTAAAAACTTGAATTGTGAAACATC
RsRf_2   CTCGGGTCAGCTATATCTAACTTTATTGAGTTTTCCAAGACATTACAAGGGAAACAGATGGTGGCTACTTGGCTAGCAATTCACATCAATTA---TTAAAAACTTGAATTGTGAAACATC
RsRf_1   CTCGGGTCAGCTATATCTAACTTTATTGAGTTTTCCAAGACATTACAAGGGAAACAGATGGTGGCTACTTGGCTAGCAATTCACATCAATTA---TTAAAAACTTGAATTGTGAAACATC
GsRf_2   CTCGGGTCAGCTATATCTAACTTTATTGAGTTTTCCAAGACATTACAAGGGAAACAGATGGTGGCTACTTGGCTAGCAATTCACATCAATTA---TTAAAAACTTGAATTGTGAAACATC
RsWf_1   CTGGGGTCAGCTATGTCTAACTTTATTGAGTTTTCCAAGACATTTCAAGGGAAACAGATGGTGGCTACTTGGCTAGCAATTCACATCAATTA---TTAAAAACTTGAATTGTGAAACATC
RsWf_2   CTGGGGTCAGCTATGTCTAACTTTATTGAGTTTTCCAAGACATTTCAAGGGAAACAGATGGTGGCTACTTGGCTAGCAATTCACATCAATTA---TTAAAAACTTGAATTGTGAAACATC
WsWf_1   CTGGGGTCAGCTATGTCTAACTTTATTGAGTTTTCCAAGACATTACAAGGGAAACAGATGGTGGCTACTTGGCTAGCAATTCACATCAATTACTATTAAAAACTTGAATTGTGAAACATC
WsWf_2   CTGGGGTCAGCTATGTCTAACTTTATTGAGTTTTCCAAGACATTACAAGGGAAACAGATGGTGGCTACTTGGCTAGCAATTCACATCAATTACTATTAAAAACTTGAATTGTGAAACATC
         ** *********** ***************************** *********************************************   *************************
 Wf-PR: 5′-CCAGTTAGACATAGCTGACCCCA

         -720                Weak MRE
GsRf_1   TCATTCTCCTCCTTTATTACACCAAACTACTTTCTTTTCTCTACTTACCGGTCAGGTCAACAGTTCTACCATTCCCCTTCTTCTTTTTACTCTTTTCGTTTAAATAAAACTAAAGATGCT
RsRf_2   TCATTCTCCTCCTTTATTACACCAAACTACTTTCTTTTCTCTACTTACCGGTCAGGTCAACAGTTCTACCATTCCCCTTCTTCTTTTTACTCTTTTCGTTTAAATAAAACTAAAGATGCT
RsRf_1   TCATTCTCCTCCTTTATTACACCAAACTACTTTCTTTTCTCTACTTACCGGTCAGGTCAACAGTTCTACCATTCCCCTTCTTCTTTTTACTCTTTTCGTTTAAATAAAACTAAAGATGCT
GsRf_2   TCATTCTCCTCCTTTATTACACCAAACTACTTTCTTTTCTCTACTTACCGGTCAGGTCAACAGTTCTACCATTCCCCTTCTTCTTTTTACTCTTTTCGTTTAAATAAAACTAAAGATGCT
RsWf_1   TCATTCTCCTCCTTTATTACACCAAACTACTTTCTTTTCTCTACTTACCGGTCAGGTCAACAGTTCTACCATTCCCCTTCTTCTTTTTACTCTTTTCGTTTAAATAAAACTAAAGATGCT
RsWf_2   TCATTCTCCTCCTTTATTACACCAAACTACTTTCTTTTCTCTACTTACCGGTCAGGTCAACAGTTCTACCATTCCCCTTCTTCTTTTTACTCTTTTCGTTTAAATAAAACTAAAGATGCT
WsWf_1   TCATTCTCCTCCTTTATTACACCAAACTACTTTCTTTTCTCTACTTACCGGTCAGGTCAACAGTTCTACCATTCCCCTTCTTCTTTTTACTCTTTTCGTTTAAATAAAACTAAAGATGCT
WsWf_2   TCATTCTCCTCCTTTATTACACCAAACTACTTTCTTTTCTCTACTTACCGGTCAGGTCAACAGTTCTACCATTCCCCTTCTTCTTTTTACTCTTTTCGTTTAAATAAAACTAAAGATGCT
         ************************************************************************************************************************

         -600                                                                                      BRE
GsRf_1   ACTCTTTTATTATGGTTGATAAACACTGGAATATTGCAAATTTCCAAATAAGAGAAAAGAAAGAGAGATATAAAACCGAATCATAGTAACACAAGTCAAAGCAACGTCTGTTTTCTCTT
RsRf_2   ACTCTTTTATTATGGTTGATAAACACTGGAATATTGCAAATTTCCAAATAAGAGAAAAGAAAGAGAGATATAAAACCGAATCATAGTAACACAAGTCAAAGCAACGTCTGTTTTCTCTT
RsRf_1   ACTCTTTTATTATGGTTGATAAACACTGGAATATTGCAAATTTCCAAATAAGAGAAAAGAAAGAGAGATATAAAACCGAATCATAGTAACACAAGTCAAAGCAACGTCTGTTTTCTCTT
GsRf_2   ACTCTTTTATTATGGTTGATAAACACTGGAATATTGCAAATTTCCAAATAAGAGAAAAGAAAGAGAGATATAAAACCGAATCATAGTAACACAAGTCAAAGCAACGTCTGTTTTCTCTT
```

```
RsWf_1     ACTCTTTTATTATGGTTGATAAACACTGGAATATTGCAAATTTCCAAATAAGAGAAAAGAAAGAGAGATGTAAAACCGAAATCATAGTAACACAAGTCAAAGCAACGTCTGTTTTCTCTT
RsWf_2     ACTCTTTTATTATGGTTGATAAACACTGGAATATTGCAAATTTCCAAATAAGAGAAAAGAAAGAGAGATGTAAAACCGAAATCATAGTAACACAAGTCAAAGCAACGTCTGTTTTCTCTT
WsWf_1     ACTCTTTTATTATGGTTGATAAACACTGGAATATTGCAAATTTCCAAATAAGAGAAAAGAAAGAGAGATGTAAAACCGAAATCATAGTAACACAAGTCAAAGCAACGTCTGTTTTCTCTT
WsWf_2     ACTCTTTTATTATGGTTGATAAACACTGGAATATTGCAAATTTCCAAATAAGAGAAAAGAAAGAGAGATGTAAAACCGAAATCATAGTAACACAAGTCAAAGCAACGTCTGTTTTCTCTT
           ********************************************************************* **************************************************

           -480   MYB-core                                                                                                   BRE
GsRf_1     GGTTGTCAGTTAGCTATTTCTCTACTGAAATATGTTATGCTTTTTTCCCAATAATATATTATAACATATTAAAAAATATGCATTCGGCATTTTTGGTTCTCCAATCAGTGGGGGACACG
RsRf_2     GTTTGTCAGTTAGCTATTTCTCTACTGAAATATGTTATGCTTTTTTCCCAATAATATATTATAACATATTAAGAAATATGCATTCGGCATTTTTGGTTCTCCAATCAGTGGGGGACACG
RsRf_1     GGTTGTCAGTTAGCTATTTCTCTACTGAAATATGTTATGCTTTTTTCCCAATAATATATTATAACATATTAAGAAATATGCATTCGGCATTTTTGGTTCTCCAATCAGTGGGGGACACG
GsRf_2     GTTTGTCAGTTAGCTATTTCTCTACTGAAATATGTTATGCTTTTTTCCCAATAATATATTATAACATATTAAGAAATATGCATTCGGCATTTTTGGTTCTCCAATCAGTGGGGGACACG
RsWf_1     GTTTGTCAGTTAGCTATTTCTCTACTAAAATATGTTATGCTTTTTTCCCAATAATATATTATAACATATTAAGAAATATGCATTCGGCATTTTTGGTTCTCCAATCCGTGGGGGACACG
RsWf_2     GTTTGTCAGTTAGCTATTTCTCTACTAAAATATGTTATGCTTTTTTCCCAATAATATATTATAACATATTAAGAAATATGCATTCGGCATTTTTGGTTCTCCAATCCGTGGGGGACACG
WsWf_1     GTTTGTCAGTTAGCTATTTCTCTACTAAAATATGTTATGCTTTTTTCCCAATAATA--TTATAACATATTAA------TGCATTCGGCATTTTTGGTTCTCCAATCCGTGGGGGACACG
WsWf_2     GTTTGTCAGTTAGCTATTTCTCTACTAAAATATGTTATGCTTTTTTCCCAATAATA--TTATAACATATTAA------TGCATTCGGCATTTTTGGTTCTCCAATCCGTGGGGGACACG
           * ************************ *****************************  **************      **************************** ************

           -360                                                                   MRE (ACII MYB)
GsRf_1     ATTACAAGACAATTACATCTTTTTATTTCATCTGTATTCTACTCTCTCATCCAACGTCTGGTTTACCAACCAGTCAGAACGCATAAAAGTCAACTTTTACTAAATCTTGAGCTTTTTGGT
RsRf_2     ATTACAAGACAATTACATCTTTTTATTTCATCTGTATTCTACTCTCTCATCCAACGTCTGGTTTACCAACCAGTCAGAACGCATAAAAGTCAACTTTTACTAAATCTTGAGCTTTTTGGT
RsRf_1     ATTACAAGACAATTACATCTTTTTATTTCATCTGTATTCTACTCTCTCATCCAACGTCTGGTTTACCAACCAGTCAGAACGCATAAAAGTCAACTTTTACTAAATCTTGAGCTTTTTGGT
GsRf_2     ATTACAAGACAATTACATCTTTTTATTTCATCTGTATTCTACTCTCTCATCCAACGTCTGGTTTACCAACCAGTCAGAACGCATAAAAGTCAACTTTTACTAAATCTTGAGCTTTTTGGT
RsWf_1     ATTACAAGACCATTTCATCTTTTTATTTCATCTGTATTCTACTCTCTCATCCGACGTCTGGTTTACCAACCAGTCAGAACGCATAAAAGTCAACTTTTACTAAATCTTGAG-TTTTTGGT
RsWf_2     ATTACAAGACCATTTCATCTTTTTATTTCATCTGTATTCTACTCTCTCATCCGACGTCTGGTTTACCAACCAGTCAGAACGCATAAAAGTCAACTTTTACTAAATCTTGAG-TTTTTGGT
WsWf_1     ATTACAAGACCATTTCATCTTTTTATTTCATCTGTATTCTACTCTCTCATCCGACGTCTGGTTTACCAACCAGTCAGAACGCATAAAAGCCAACTTTTACTAAATCTTGAG-TTTTTGGT
WsWf_2     ATTACAAGACCATTTCATCTTTTTATTTCATCTGTATTCTACTCTCTCATCCGACGTCTGGTTTACCAACCAGTCAGAACGCATAAAAGCCAACTTTTACTAAATCTTGAG-TTTTTGGT
           ********** *** ************************************* ************************************ ********************* ********

           -240
GsRf_1     CATGCATTTATACACATACATAGAATTAATCTAGATTACTTTACTTATGTCAGCATGTTACGAAAACACTAAAATGGTATATATATACATACATATTGAATTAACCGTGGTCCCAAACCA
RsRf_2     CATGCATTTATACACATACATAGAATTAATCTAGATTACTTTACTTATGTCAGCATGTTACGAAAACACTAAAATGGTATATATATACATACATATTGAATTAACCGTGGTCCCAAACCA
RsRf_1     CATGCATTTATACACATACATAGAATTAATCTAGATTACTTTACTTATGTCAGCATGTTACGAAAACACTAAAATGGTATATATATACATACATATTGAATTAACCGTGGTCCCAAACCA
GsRf_2     CATGCATTTATACACATACATAGAATTAATCTAGATTACTTTACTTATGTCAGCATGTTACGAAAACACTAAAATGGTATATATATACATACATATTGAATTAACCGTGGTCCCAAACCA
RsWf_1     CATGCATTTATACACATACATAGAATTAATCTAGATTACTTTACTTATGTCAGCATGTTACGAAAACACTAAAATGGTATATATATGCATACATATTGAATTAACCGTGGTCCCAAACCA
RsWf_2     CATGCATTTATACACATACATAGAATTAATCTAGATTACTTTACTTATGTCAGCATGTTACGAAAACACTAAAATGGTATATATATGCATACATATTGAATTAACCGTGGTCCCAAACCA
WsWf_1     CATGCATTTATACACATACATAGAATTAATCTAGATTACTTTACTTATGTCAGCATGTTACGAAAACACTAAAATGGTATATATATGCATACATATTGAATTAAC-GTGGTCCCAAACCA
WsWf_2     CATGCATTTATACACATACATAGAATTAATCTAGATTACTTTACTTATGTCAGCATGTTACGAAAACACTAAAATGGTATATATATGCATACATATTGAATTAAC-GTGGTCCCAAACCA
           ************************************************************************************** ****************** **************

           -120                 MRE                 Radish-F                                -33
GsRf_1     ATTTAAATCAGCATAAAGAATATCTTCCTCTTTCATATCAAAACCCCTTCAGAGTTGCAAGATCTTACTCTC--------------AGAGAGCTAATACATGTTTTCATCTCGGGGAAAA
RsRf_2     ATTTAAATCAGCATAAAGAATATCTTCCTCTTTCATATCAAAACCCCTTCAGAGTTGCAAGATCTTACTCTC--------------AGAGAGCTAATACATGTTTTCATCTCGGGGAAAA
RsRf_1     ATTTAAATCAGCATAAAGAATATCTTCCTCTTTCATATCAAAACCCCTTCAGAGTTGCAAGATCTTACTCTC--------------AGAGAGCTAATACATGTTTTCATCTCGGGGAAAA
GsRf_2     ATTTAAATCAGCATAAAGAATATCTTCCTCTTTCATATCAAAACCCCTTCAGAGTTGCAAGATCTTACTCTC--------------AGAGAGCTAATACATGTTTTCATCTCGGGGAAAA
RsWf_1     ATTTAAATCAGCATAAAGAATATCTTCCTCTTTCATATCAAAACCCCTTCAGAGTTGCAAGATCTTACTCTCTTCAGAGTTGCGAGAGAGAGCTAATATATGTTTTCATCTCGGGGAAAA
RsWf_2     ATTTAAATCAGCATAAAGAATATCTTCCTCTTTCATATCAAAACCCCTTCAGAGTTGCAAGATCTTACTCTCTTCAGAGTTGCGAGAGAGAGCTAATATATGTTTTCATCTCGGGGAAAA
WsWf_1     ATTTAAATCAGCATAAAGAATATCTTCCTCTTTCATATCAAAACCCCTTCAGAGTTGCAAGATCTTACTCTCTTCAGAGTTGCGAGAGAGAGCTAATATATGTTTTCATCTCGGGGAAAA
WsWf_2     ATTTAAATCAGCATAAAGAATATCTTCCTCTTTCATATCAAAACCCCTTCAGAGTTGCAAGATCTTACTCTCTTCAGAGTTGCGAGAGAGAGCTAATATATGTTTTCATCTCGGGGAAAA
           ************************************************************************              ************ *********************
                                                                              Wf-F

           0  Exon 1                                                                         Radish-R
GsRf_1     ATGGATGAATCAAGTATTATACCGGTATGGAAAGTGATCGGAGCTGAGGAAAAAGAGATTCAAGGGCTACTTAAGGCGGTGGTGCAATCTGTGGGGTGGACTTATAGTCTCTTCTGGCAA  120
RsRf_2     ATGGATGAATCAAGTATTATACCGGTATGGAAAGTGATCGGAGCTGAGGAAAAAGAGATTCAAGGGCTACTTAAGGCGGTGGTGCAATCTGTGGGGTGGACTTATAGTCTCTTCTGGCAA
RsRf_1     ATGGATGAATCAAGTATTATACCGGTATGGAAAGTGATCGGAGCTGAGGAAAAAGAGATTCAAGGGCTACTTAAGGCGGTGGTGCAATCTGTGGGGTGGACTTATAGTCTCTTCTGGCAA
GsRf_2     ATGGATGAATCAAGTATTATACCGGTATGGAAAGTGATCGGAGCTGAGGAAAAAGAGATTCAAGGGCTACTTAAGGCGGTGGTGCAATCTGTGGGGTGGACTTATAGTCTCTTCTGGCAA
RsWf_1     ATGGATGAATCAAGTATTATACCGGTATGGAAAGTGATCGGAGCTGAGGGAAAAGAGATTCAAGGGCTACTTAAGGCGGTGGTGCAATCTGTGGGGTGGACTTATAGTCTCTTCTGGCAA
RsWf_2     ATGGATGAATCAAGTATTATACCGGTATGGAAAGTGATCGGAGCTGAGGGAAAAGAGATTCAAGGGCTACTTAAGGCGGTGGTGCAATCTGTGGGGTGGACTTATAGTCTCTTCTGGCAA
WsWf_1     ATGGATGAATCAAGTATTATACCGGTATGGAAAGTGATCGGAGCTGAGGAAAAAGAGATTCAAGGGCTACTTAAGGCGGTGGTGCAATCTGTGGGGTGGACTTATAGTCTCTTCTGGCAA
WsWf_2     ATGGATGAATCAAGTATTATACCGGTATGGAAAGTGATCGGAGCTGAGGAAAAAGAGATTCAAGGGCTACTTAAGGCGGTGGTGCAATCTGTGGGGTGGACTTATAGTCTCTTCTGGCAA
           ************************************************* **********************************************************************

GsRf_1     CTTTGTCCTCAACGAAGGTTCTCTTTTCATTTCATCCATCTCTCACAATATATAAAGCAATATATTAATTTATCCTTATTAATTATAAC------------AAGTAGAAGTGACTAA-TG  240
RsRf_2     CTTTGTCCTCAACGAAGGTTCTCTTTTCATTTCATCCATCTCTCACAATATATAAAGCAATATATTAATTTATCCTTATTAATTATAAC------------AAGTAGAAGTGACTAA-TG
RsRf_1     CTTTGTCCTCAACGAAGGTTCTCTTTTCATTTCATCCATCTCTCACAATATATAAAGCAATATATTAATTTATCCTTATTAATTATAAC------------AAGTAGAAGTGACTAA-TG
GsRf_2     CTTTGTCCTCAACGAAGGTTCTCTTTTCATTTCATCCATCTCTCACAATATATAAAGCAATATATTAATTTATCCTTATTAATTATAAC------------AAGTAGAAGTGACTAA-TG
RsWf_1     CTTTGTCCTCAACGAAGGTTCTCTTTTCATTTCATCCATCTCTCACAATATATAAAGCAATATATTAATTTATCCTTATTAATTATAAC------------AAGTAGAAGTGACTAAATG
RsWf_2     CTTTGTCCTCAACGAAGGTTCTCTTTTCATTTCATCCATCTCTCACAATATATAAAGCAATATATTAATTTATCCTTATTAATTATAAC------------AAGTAGAAGTGACTAAATG
WsWf_1     CTTTGTCCTCAACGAAGGTTCTCTTTTCATTTCATCCATCTCTCACAATATATAAAGCAATATATTAATTTATCCTTATTAATTATAACCTCAATTATAACAAGTAGAAGTGACTAAATG
WsWf_2     CTTTGTCCTCAACGAAGGTTCTCTTTTCATTTCATCCATCTCTCACAATATATAAAGCAATATATTAATTTATCCTTATTAATTATAACCTCAATTATAACAAGTAGAAGTGACTAAATG
           *****************************************************************************************            **************** **

                       Exon 2
GsRf_1     TTAAATCGATTAGGAAATTGGTGTGGAGTAGTGGATTCTACAACGGTGCAATAAAGACTAGAAAGACAACTCAGCCGGCGGAAATAACGGCTGAAGAGGCTGCGTTGGAGAGAAGCCAAC  360
RsRf_2     TTAAATCGATTAGGAAATTGGTGTGGAGTAGTGGATTCTACAACGGTGCAATAAAGACTAGAAAGACAACTCAGCCGGCGGAAATAACGGCTGAAGAGGCTGCGTTGGAGAGAAGCCAAC
RsRf_1     TTAAATCGATTAGGAAATTGGTGTGGAGTAGTGGATTCTACAACGGTGCAATAAAGACTAGAAAGACAACTCAGCCGGCGGAAATAACGGCTGAAGAGGCTGCGTTGGAGAGAAGCCAAC
GsRf_2     TTAAATCGATTAGGAAATTGGTGTGGAGTAGTGGATTCTACAACGGTGCAATAAAGACTAGAAAGACAACTCAGCCGGCGGAAATAACGGCTGAAGAGGCTGCGTTGGAGAGAAGCCAAC
RsWf_1     TTAAATCGATTAGGAAATTGGTGTGGAGTAGTGGATTCTACAACGGTGCAATAAAGACTAGAAAGACAACTCAGCCGGCGGAAATAACGGCTGAAGAGGCTGCGTTGGAGAGAAGCCAAC
RsWf_2     TTAAATCGATTAGGAAATTGGTGTGGAGTAGTGGATTCTACAACGGTGCAATAAAGACTAGAAAGACAACTCAGCCGGCGGAAATAACGGCTGAAGAGGCTGCGTTGGAGAGAAGCCAAC
WsWf_1     TTAAATCGATTAGGAAATTGGTGTGGAGTAGTGGATTCTACAACGGTGCAATAAAGACTAGAAAGACAACTCAGCCGGCGGAAATAACGGCTGAAGAGGCTGCGTTGGAGAGAAGCCAAC
WsWf_2     TTAAATCGATTAGGAAATTGGTGTGGAGTAGTGGATTCTACAACGGTGCAATAAAGACTAGAAAGACAACTCAGCCGGCGGAAATAACGGCTGAAGAGGCTGCGTTGGAGAGAAGCCAAC
           ************************************************************************************************************************

                                                                                     SSR-F
GsRf_1     AGCTCATGGAGCTTTACCAGACGCTTTTTGCCGGAGAATCATCGATGGAAGCGAGGGCTTGCACAGCACTGTCGCCTGAGGATTTGACGGACACTGAATGGTTTTATGTGCTGTGTCTCA  480
RsRf_2     AGCTCATGGAGCTTTACCAGACGCTTTTTGCCGGAGAATCATCGATGGAAGCGAGGGCTTGCACAGCACTGTCGCCTGAGGATTTGACGGACACTGAATGGTTTTATGTGCTGTGTCTCA
RsRf_1     AGCTCATGGAGCTTTACCAGACGCTTTTTGCCGGAGAATCATCGATGGAAGCGAGGGCTTGCACAGCACTGTCGCCTGAGGATTTGACGGACACTGAATGGTTTTATGTGCTGTGTCTCA
GsRf_2     AGCTCATGGAGCTTTACCAGACGCTTTTTGCCGGAGAATCATCGATGGAAGCGAGGGCTTGCACAGCACTGTCGCCTGAGGATTTGACGGACACTGAATGGTTTTATGTGCTGTGTCTCA
RsWf_1     AGCTCATGGAGCTTTACCAGACGCTTTTTGCCGGAGAATCATCGATGGAAGCGAGGGCTTCCACAGCACTGTCGCCTGAGGATTTGACGGACACTGAATGGTTTTATGTGCTGTGTCTCA
RsWf_2     AGCTCATGGAGCTTTACCAGACGCTTTTTGCCGGAGAATCATCGATGGAAGCGAGGGCTTCCACAGCACTGTCGCCTGAGGATTTGACGGACACTGAATGGTTTTATGTGCTGTGTCTCA
WsWf_1     AGCTCATGGAGCTTTACCAGACGCTTTTTGCCGGAGAATCATCGATGGAAGCGAGGGCTTGCACAGCACTGTCGCCTGAGGATTTGACGGACACTGAATGGTTTTATGTGCTGTGTCTCA
WsWf_2     AGCTCATGGAGCTTTACCAGACGCTTTTTGCCGGAGAATCATCGATGGAAGCGAGGGCTTGCACAGCACTGTCGCCTGAGGATTTGACGGACACTGAATGGTTTTATGTGCTGTGTCTCA
```

```
           ************************************************************ ***********************************************************


GsRf_1     CTTACTCTTTGAACCTCCTTCTGGGTACAACAACTCTTTCTCTCTCTCTCTCTCTCTCTCTCTCTCTCTCTCTCTCTCTCTCTCTCTCTCTCTCTCTCTCTCTCTCTC-------  600
RsRf_2     CTTACTCTTTGAACCTCCTTCTGGGTACAACAACTCTTTCTCTCTCTCTCTCTCTCTCTCTCTCTCTCTCTCTCTCTCTCTCTCTCTCTCTCTCTCTCTCTCTCTCTCTC-----
RsRf_1     CTTACTCTTTGAACCTCCTTCTGGGTACAACAACTCTTTCTCTCTCTCTCTCTCTCTCTCTCTCTCTCTCTCTCTCTCTCTCTCTCTCTCTCTCTCTCTCTC-----------
GsRf_2     CTTACTCTTTGAACCTCCTTCTGGGTACAACAACTCTTTCTCTCTCTCTCTCTCTCTCTCTCTCTCTCTCTCTCTCTCTCTCTCTCTCTCTCTCTCTCTCTC-----------
RsWf_1     CTTACTCTTTGAACCTCCTTCTGGGTACAACAACTCTTTCTCTCTCTCTCTCTCTCTCTCTCTC-------------------------------------------------------
RsWf_2     CTTACTCTTTGAACCTCCTTCTGGGTACAACAACTCTTTCTCTCTCTCTCTCTCTCTCTCTCTC-------------------------------------------------------
WsWf_1     CTTACTCTTTGAACCTCCTTCTGGGTACAACAACTCTTTCTCTCTCTCTCTCTCTCTCTCTCTCTCTCTCTCTCTCTCTCTCTCTCTCTCTCTCTCTCTCTCTCTCTCTCTCTCT
WsWf_2     CTTACTCTTTGAACCTCCTTCTGGGTACAACAACTCTTTCTCTCTCTCTCTCTCTCTCTCTCTCTCTCTCTCTCTCTCTCTCTCTCTCTCTCTCTCTCTCTCTCTCTCTCTCTCT
           *****************************************************************


GsRf_1     ---------------------------------------------------------CAAAGTTTTTTCTTTCTTTCTATAAAGACTACTCAGAGTTTCTTAATTTGTCCTTTTCATCTC  720
RsRf_2     ---------------------------------------------------------CAAAGTTTTTTCTTTCTTTCTATAAAGACTACTCAGAGTTTCTTAATTTGTCCTTTTCATCTC
RsRf_1     ---------------------------------------------------------CAAAGTTTTTTCTTTCTTTCTATAAAGACTACTCAGAGTTTCTTAATTTGTCCTTTTCATCTC
GsRf_2     ---------------------------------------------------------CAAAGTTTTTTCTTTCTTTCTATAAAGACTACTCAGAGTTTCTTAATTTGTCCTTTTCATCTC
RsWf_1     ---------------------------------------------------------CAAAGTTTTTTCTTTCTTTCTATAAAGACTACTCAGAGTTTCTTAATTTGTCCTTTTCATCTC
RsWf_2     ---------------------------------------------------------CAAAGTTTTTTCTTTCTTTCTATAAAGACTACTCAGAGTTTCTTAATTTGTCCTTTTCATCTC
WsWf_1     CTCTCTCTCTCTCTCTCTCTCTCTCTCTCTCTCTCTCTCTCTCTCTCTCTCTCTCTCCAAAGTTTTTTCTTTCTTTCTATAAAGGCTACTCAGAGTTTCTTAATTTGTCCTTTTCATCTC
WsWf_2     CTCTCTCTCTCTCTCTCTCTCTCTCTCTCTCTCTCTCTCTCTCTCTCTCTCTCT--CCAAAGTTTTTTCTTTCTTTCTATAAAGGCTACTCAGAGTTTCTTAATTTGTCCTTTTCATCTC
                                                                    *************************** ***********************************

                                                                                  SSR-R
GsRf_1     CTCTTAGTGAAGACAAAAATAGTATTGTGTGTTAAATGCGAATCGAAAATACTATGGAGCATTAAAGACAAACTGAGGAGTTTAAGTTACTGAAAGAAGAAATTTATTGAAGTTTGTGAA  840
RsRf_2     CTCTTAGTGAAGACAAAAATAGTATTGTGTGTTAAATGCGAATCGAAAATACTATGGAGCATTAAAGACAAACTGAGGAGTTTAAGTTACTGAAAGAAGAAATTTATTGAAGTTTGTGAA
RsRf_1     CTCTTAGTGAAGACAAAAATAGTATTGTGTGTTAAATGCGAATCGAAAATACTATGGAGCATTAAAGACAAACTGAGGAGTTTAAGTTACTGAAAGAAGAAATTTATTGAAGTTTGTGAA
GsRf_2     CTCTTAGTGAAGACAAAAATAGTATTGTGTGTTAAATGCGAATCGAAAATACTATGGAGCATTAAAGACAAACTGAGGAGTTTAAGTTACTGAAAGAAGAAATTTATTGAAGTTTGTGAA
RsWf_1     CTCTTAGTGAAGACAAAAATAGTATTGTGTGTTAAATGCGAATCGAAAATACTATGGAGCATTAAAGACAAACTGAGGAGTTTAAGTTACTGAAAGAAGAAATTTATTGAAGTTTGTGAA
RsWf_2     CTCTTAGTGAAGACAAAAATAGTATTGTGTGTTAAATGCGAATCGAAAATACTATGGAGCATTAAAGACAAACTGAGGAGTTTAAGTTACTGAAAGAAGAAATTTATTGAAGTTTGTGAA
WsWf_1     CTCTTAGTGAAGACAAAAATAGTATTGTGTGTTAAATGCGAATCGAAAATACTATGGAGCATTAAAGACAAACTGAGGAGTTTAAGTTACTGAAAGAAGAAATTTATTGAAGTTTGTGAA
WsWf_2     CTCTTAGTGAAGACAAAAATAGTATTGTGTGTTAAATGCGAATCGAAAATACTATGGAGCATTAAAGACAAACTGAGGAGTTTAAGTTACTGAAAGAAGAAATTTATTGAAGTTTGTGAA
           ************************************************************************************************************************


GsRf_1     AACGTACACTTCATTTTGGTGAACATAATTGGACCGTTGAGATTCGTATTGGTTTGTTTATTGATTATCTAAAGTAGAACTGTAGAAGCATATAAAAAATGCATAACAAAGTGTGTTAGT  960
RsRf_2     AACGTACACTTCATTTTGGTGAACATAATTGGACCGTTGAGATTCGTATTGGTTTGTTTATTGATTATCTAAAGTAGAACTGTAGAAGCATATAAAAAATGCATAACAAAGTGTGTTAGT
RsRf_1     AACGTACACTTCATTTTGGTGAACATAATTGGACCGTTGAGATTCGTATTGGTTTGTTTATTGATTATCTAAAGTAGAACTGTAGAAGCATATAAAAAATGCATAACAAAGTGTGTTAGT
GsRf_2     AACGTACACTTCATTTTGGTGAACATAATTGGACCGTTGAGATTCGTATTGGTTTGTTTATTGATTATCTAAAGTAGAACTGTAGAAGCATATAAAAAATGCATAACAAAGTGTGTTAGT
RsWf_1     AACGTACACTTCATTTTGGTGAACATAATTGGACCGTTGAGATTCGTATTGGTTTGTTTATTGATTATCTAAAGTAGAACTGTAGAAGCATATAAAAAATGCATAACAAAGTGTGTTAGT
RsWf_2     AACGTACACTTCATTTTGGTGAACATAATTGGACCGTTGAGATTCGTATTGGTTTGTTTATTGATTATCTAAAGTAGAACTGTAGAAGCATATAAAAAATGCATAACAAAGTGTGTTAGT
WsWf_1     AACGTACACTTCATTTTGGTGAACATAATTGGACCGTTGAGATTCGTATTGGTTTGTTTATTGATTATCTAAAGTAGAACTGTAGAAGCATATAAAAAATGCATAACAAAGTGTGTTAGT
WsWf_2     AACGTACACTTCATTTTGGTGAACATAATTGGACCGTTGAGATTCGTATTGGTTTGTTTATTGATTATCTAAAGTAGAACTGTAGAAGCATATAAAAAATGCATAACAAAGTGTGTTAGT
           ************************************************************************************************************************

                                                                                                    Exon 3
GsRf_1     TATCGTTATAATTAATGTTTTTTTCTCTATGGAGGAGAAAAAAAAATCAAAATATAAATGTAGAAGTATTAATTTGTAGGATGCCAGGAAAGGCGTATGCGAGGAGGAAGCAAGTATGGA  1080
RsRf_2     TATCGTTATAATTAATGTTTTTTTCTCTATGGAGGAGAAAAAAAAATCAAAATATAAATGTAGAAGTATTAATTTGTAGGATGCCAGGAAAGGCGTATGCGAGGAGGAAGCAAGTATGGA
RsRf_1     TATCGTTATAATTAATGTTTTTTTCTCTATGGAGGAGAAAAAAAAATCAAAATATAAATGTAGAAGTATTAATTTGTAGGATGCCAGGAAAGGCGTATGCGAGGAGGAAGCAAGTATGGA
GsRf_2     TATCGTTATAATTAATGTTTTTTTCTCTATGGAGGAGAAAAAAAAATCAAAATATAAATGTAGAAGTATTAATTTGTAGGATGCCAGGAAAGGCGTATGCGAGGAGGAAGCAAGTATGGA
RsWf_1     TATCGTTATAATTAATGTTTTTTTCTCTATGGAGGAGAAAAAAAGATCAAAATATAAATGTAGAAGTATTAATTTGTAGGATGCCAGGAAAGGCGTATGCGAGGAGGAAGCAAGTATGGA
RsWf_2     TATCGTTATAATTAATGTTTTTTTCTCTATGGAGGAGAAAAAAAGATCAAAATATAAATGTAGAAGTATTAATTTGTAGGATGCCAGGAAAGGCGTATGCGAGGAGGAAGCAAGTATGGA
WsWf_1     TATCGTTATAATTAATGTTTTTTTCTCTATGGAGGAGAAAAAAAAATCAAAATATAAATGTAGAAGTATTAATTTGTAGGATGCCAGGAAAGGCGTATGCGAGGAGGAAGCAAGTATGGA
WsWf_2     TATCGTTATAATTAATGTTTTTTTCTCTATGGAGGAGAAAAAAAAATCAAAATATAAATGTAGAAGTATTAATTTGTAGGATGCCAGGAAAGGCGTATGCGAGGAGGAAGCAAGTATGGA
           ******************************************** ***************************************************************************


GsRf_1     TGAGTGGTGTAAATGAGGTTGACAGTAAAATCTTCTCTAGGGCTATTTCTGCAAAGGTTTATTTCCTTTTATTCATTCACCACTACACTGTGTATCTACTTCTACTTATTTAGATATACG  1200
RsRf_2     TGAGTGGTGTAAATGAGGTTGACAGTAAAATCTTCTCTAGGGCTATTTCTGCAAAGGTTTATTTCCTTTTATTCATTCACCACTACACTGTGTATCTACTTCTACTTATTTAGATATACG
RsRf_1     TGAGTGGTGTAAATGAGGTTGACAGTAAAATCTTCTCTAGGGCTATTTCTGCAAAGGTTTATTTCCTTTTATTCATTCACCACTACACTGTGTATCTACTTCTACTTATTTAGATATACG
GsRf_2     TGAGTGGTGTAAATGAGGTTGACAGTAAAATCTTCTCTAGGGCTATTTCTGCAAAGGTTTATTTCCTTTTATTCATTCACCACTACACTGTGTATCTACTTCTACTTATTTAGATATACG
RsWf_1     TGAGTGGTGTAAATGAGGTTGACAGTAAAATCTTCTCTAGGGCTATTTCTGCAAAGGTTTATTTCCTTTTATTCATTCACCACTACACTGTGTATCTACTTCTACTTATTTAGATATACG
RsWf_2     TGAGTGGTGTAAATGAGGTTGACAGTAAAATCTTCTCTAGGGCTATTTCTGCAAAGGTTTATTTCCTTTTATTCATTCACCACTACACTGTGTATCTACTTCTACTTATTTAGATATACG
WsWf_1     TGAGTGGTGTAAATGAGGTTGACAGTAAAATCTTCTCTAGGGCTATTTCTGCAAAGGTTTATTTCCTTTTATTCATTCACCACTACACGGTGTATCTACTTCTACTTATTTAGATATACG
WsWf_2     TGAGTGGTGTAAATGAGGTTGACAGTAAAATCTTCTCTAGGGCTATTTCTGCAAAGGTTTATTTCCTTTTATTCATTCACCACTACACGGTGTATCTACTTCTACTTATTTAGATATACG
           **************************************************************************************** *******************************

                                                                                         Exon 4
GsRf_1     AAATTTTATATTATCTCATTTCAAACTAATTAATTTTATCTTCTTAATGCTCTTTCTACACTAGAGTGCCAAAATTCAGGTAAATGTTGCCGTTATTATTTTATTTTCGTAGAAATGAAA  1320
RsRf_2     AAATTTTATATTATCTCATTTCAAACTAATTAATTTTATCTTCTTAATGCTCTTTCTACACTAGAGTGCCAAAATTCAGGTAAATGTTGCCGTTATTATTTTATTTTCGTAGAAATGAAA
RsRf_1     AAATTTTATATTATCTCATTTCAAACTAATTAATTTTATCTTCTTAATGCTCTTTCTACACTAGAGTGCCAAAATTCAGGTAAATGTTGCCGTTATTATTTTATTTTCGTAGAAATGAAA
GsRf_2     AAATTTTATATTATCTCATTTCAAACTAATTAATTTTATCTTCTTAATGCTCTTTCTACACTAGAGTGCCAAAATTCAGGTAAATGTTGCCGTTATTATTTTATTTTCGTAGAAATGAAA
RsWf_1     AAATTTTATATTATCTCATTTCAAACTAATTAATTTTATCTTCTTAATGCTCTTTCTACACTAGAGTGCCAAAATTCAGGTAAATGTTGCCGTTATTATTTTATTTTCGTAAAAATGAAA
RsWf_2     AAATTTTATATTATCTCATTTCAAACTAATTAATTTTATCTTCTTAATGCTCTTTCTACACTAGAGTGCCAAAATTCAGGTAAATGTTGCCGTTATTATTTTATTTTCGTAAAAATGAAA
WsWf_1     AAATTTTATATTATCTCATTTCAAACTAATTAATTTTATCTTCTTAATGCTCTTTCTACACTAGAGTGCCAAAATTCAGGTAAATGTTGCCGTTATTATTTTATTTTCGTAGAAATGAAA
WsWf_2     AAATTTTATATTATCTCATTTCAAACTAATTAATTTTATCTTCTTAATGCTCTTTCTACACTAGAGTGCCAAAATTCAGGTAAATGTTGCCGTTATTATTTTATTTTCGTAGAAATGAAA
           *************************************************************************************************************** ********

                                                                                                 Exon 5
GsRf_1     GGTATCAGTTAACAGAGTTATTATTTTACATATTTGAGAATTGTTTTGTGATGAAAAAACAAATAAAAACAGACAGTGGTTTGCATTCCCGTGCTTGATGGCGTTTTGGAAATAGGCACA  1440
RsRf_2     GGTATCAGTTAACAGAGTTATTATTTTACATATTTGAGAATTGTTTTGTGATGAAAAAACAAATAAAAACAGACAGTGGTTTGCATTCCCGTGCTTGATGGCGTTTTGGAAATAGGCACA
RsRf_1     GGTATCAGTTAACAGAGTTATTATTTTACATATTTGAGAATTGTTTTGTGATGAAAAAACAAATAAAAACAGACAGTGGTTTGCATTCCCGTGCTTGATGGCGTTTTGGAAATAGGCACA
GsRf_2     GGTATCAGTTAACAGAGTTATTATTTTACATATTTGAGAATTGTTTTGTGATGAAAAAACAAATAAAAACAGACAGTGGTTTGCATTCCCGTGCTTGATGGCGTTTTGGAAATAGGCACA
RsWf_1     GGTATCAGTTAACAGAGTTATTATTTTACATATTTGAGAATTGTTTTGTGATGAAAAAACAAATAAAAACAGACAGTGGTTTGCATTCCCGTGCTTGATGGCGTTTTGGAAATAGGCACA
RsWf_2     GGTATCAGTTAACAGAGTTATTATTTTACATATTTGAGAATTGTTTTGTGATGAAAAAACAAATAAAAACAGACAGTGGTTTGCATTCCCGTGCTTGATGGCGTTTTGGAAATAGGCACA
WsWf_1     GGTATCAGTTAACAGAGTTATTATTTTACATATTTGAGAATTGTTTTGTGATGAAAAAACAAATAAAAACAGACAGTGGTTTGCATTCCCGTGCTTGATGGCGTTTTGGAAATAGGCACA
WsWf_2     GGTATCAGTTAACAGAGTTATTATTTTACATATTTGAGAATTGTTTTGTGATGAAAAAACAAATAAAAACAGACAGTGGTTTGCATTCCCGTGCTTGATGGCGTTTTGGAAATAGGCACA
```

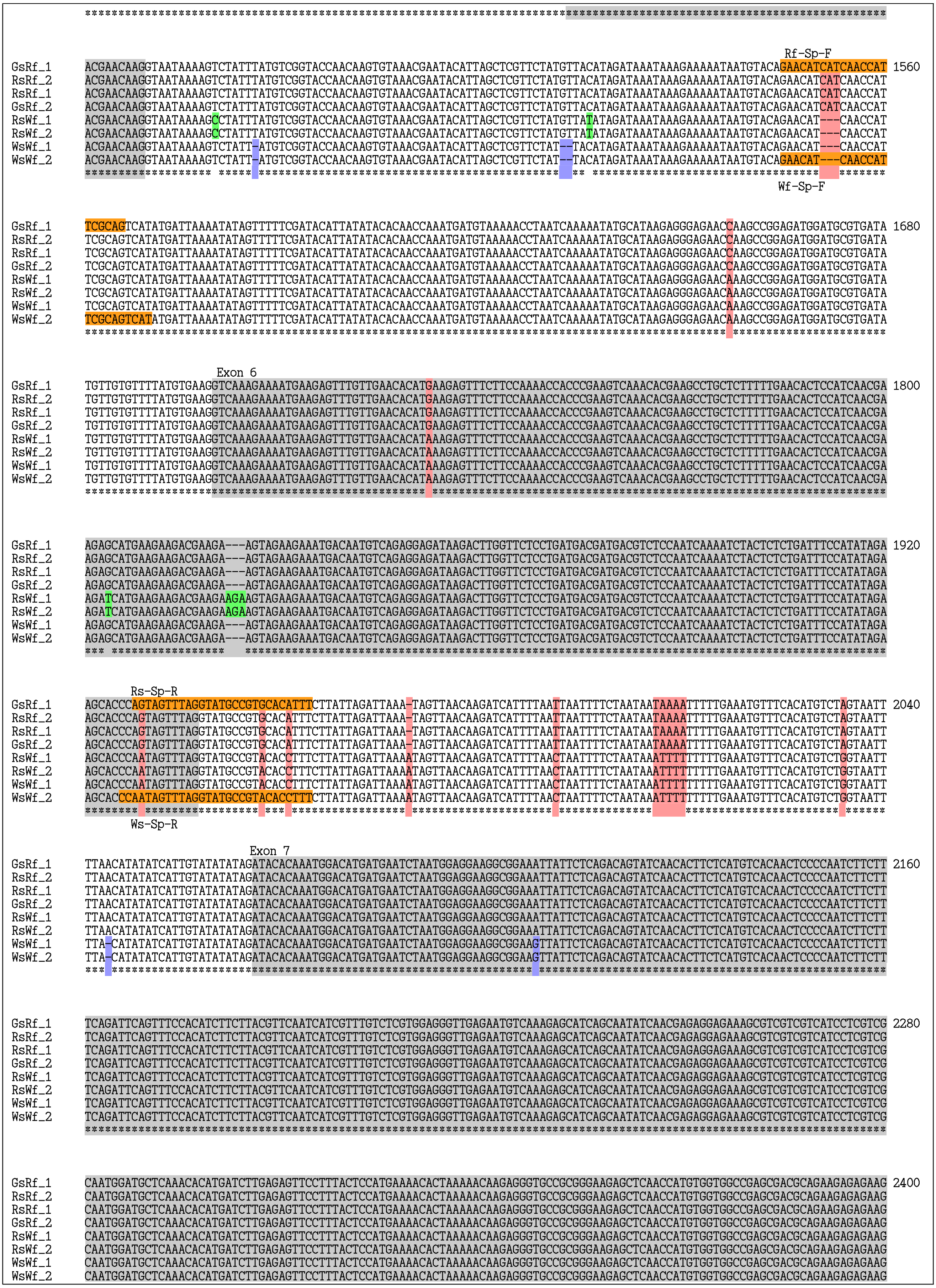
Rf-Sp-F
Wf-Sp-F
Exon 6
Rs-Sp-R
Ws-Sp-R
Exon 7
GsRf_1
RsRf_2
RsRf_1
GsRf_2
RsWf_1
RsWf_2
WsWf_1
WsWf_2
1560
1680
1800
1920
2040
2160
2280
2400

```
            ************************************************************************************************************************

GsRf_1      CTTAACGAGAGATTCATAACGTTGAGATCATTGGTTCCATTTGTGACCAAGATGGATAAAGTCTCGATCCTTGGAGACACCATTGATTACGTAAACCATCTTTGTAAGAGGATCCATGAG 2520
RsRf_2      CTTAACGAGAGATTCATAACGTTGAGATCATTGGTTCCATTTGTGACCAAGATGGATAAAGTCTCGATCCTTGGAGACACCATTGATTACGTAAACCATCTTTGTAAGAGGATCCATGAG
RsRf_1      CTTAACGAGAGATTCATAACGTTGAGATCATTGGTTCCATTTGTGACCAAGATGGATAAAGTCTCGATCCTTGGAGACACCATTGATTACGTAAACCATCTTTGTAAGAGGATCCATGAG
GsRf_2      CTTAACGAGAGATTCATAACGTTGAGATCATTGGTTCCATTTGTGACCAAGATGGATAAAGTCTCGATCCTTGGAGACACCATTGATTACGTAAACCATCTTTGTAAGAGGATCCATGAG
RsWf_1      CTTAACGAGAGATTCATAACGTTGAGATCATTGGTTCCATTTGTGACCAAGATGGATAAAGTCTCGATCCTTGGAGACACCATTGATTACGTAAACCATCTTTGTAAGAGGATCCATGAG
RsWf_2      CTTAACGAGAGATTCATAACGTTGAGATCATTGGTTCCATTTGTGACCAAGATGGATAAAGTCTCGATCCTTGGAGACACCATTGATTACGTAAACCATCTTTGTAAGAGGATCCATGAG
WsWf_1      CTTAACGAGAGATTCATAACGTTGAGATCATTGGTTCCATTTGTGACCAAGATGGATAAAGTCTCGATCCTTGGAGACACCATTGATTACGTAAACCATCTTTGTAAGAGGATCCATGAG
WsWf_2      CTTAACGAGAGATTCATAACGTTGAGATCATTGGTTCCATTTGTGACCAAGATGGATAAAGTCTCGATCCTTGGAGACACCATTGATTACGTAAACCATCTTTGTAAGAGGATCCATGAG
            ************************************************************************************************************************

GsRf_1      CTGGAATCTACTCATCACGAGCCAAACCAAAAGCGGATGCGTATCGGTAAGGGAAGAACGTGGGAAGAGGTGGAGGTTTCCATTATAGAGAGCGATGTTTTGTTAGAGATGAGATGCGAG 2640
RsRf_2      CTGGAATCTACTCATCACGAGCCAAACCAAAAGCGGATGCGTATCGGTAAGGGAAGAACGTGGGAAGAGGTGGAGGTTTCCATTATAGAGAGCGATGTTTTGTTAGAGATGAGATGCGAG
RsRf_1      CTGGAATCTACTCATCACGAGCCAAACCAAAAGCGGATGCGTATCGGTAAGGGAAGAACGTGGGAAGAGGTGGAGGTTTCCATTATAGAGAGCGATGTTTTGTTAGAGATGAGATGCGAG
GsRf_2      CTGGAATCTACTCATCACGAGCCAAACCAAAAGCGGATGCGTATCGGTAAGGGAAGAACGTGGGAAGAGGTGGAGGTTTCCATTATAGAGAGCGATGTTTTGTTAGAGATGAGATGCGAG
RsWf_1      CTGGAATCTACTCATCACGAGCCAAACCAAAAGCGGATGCGTATCGGTAAGGGAAGAACGTGGGAAGAGGTGGAGGTTTCCATTATAGAGAGCGATGTTTTGTTAGAGATGAGATGCGAG
RsWf_2      CTGGAATCTACTCATCACGAGCCAAACCAAAAGCGGATGCGTATCGGTAAGGGAAGAACGTGGGAAGAGGTGGAGGTTTCCATTATAGAGAGCGATGTTTTGTTAGAGATGAGATGCGAG
WsWf_1      CTGGAATCTACTCATCACGAGCCAAACCAAAAGCGGATGCGTATCGGTAAGGGAAGAACGTGGGAAGAGGTGGAGGTTTCCATTATAGAGAGCGATGTTTTGTTAGAGATGAGATGCGAG
WsWf_2      CTGGAATCTACTCATCACGAGCCAAACCAAAAGCGGATGCGTATCGGTAAGGGAAGAACGTGGGAAGAGGTGGAGGTTTCCATTATAGAGAGCGATGTTTTGTTAGAGATGAGATGCGAG
            ************************************************************************************************************************

GsRf_1      TACCGAGATGGTTTATTGCTCAACATTCTTCAGGTACTTAAGGAGCTGGGTATAGAGACCACTGCAGTTCACACCGCCGTGAACGACCATGATTTTGAGGCAGAGATAAGGGCGAAAGTG 2760
RsRf_2      TACCGAGATGGTTTATTGCTCAACATTCTTCAGGTACTTAAGGAGCTGGGTATAGAGACCACTGCAGTTCACACCGCCGTGAACGACCATGATTTTGAGGCAGAGATAAGGGCGAAAGTG
RsRf_1      TACCGAGATGGTTTATTGCTCAACATTCTTCAGGTACTTAAGGAGCTGGGTATAGAGACCACTGCAGTTCACACCGCCGTGAACGACCATGATTTTGAGGCAGAGATAAGGGCGAAAGTG
GsRf_2      TACCGAGATGGTTTATTGCTCAACATTCTTCAGGTACTTAAGGAGCTGGGTATAGAGACCACTGCAGTTCACACCGCCGTGAACGACCATGATTTTGAGGCAGAGATAAGGGCGAAAGTG
RsWf_1      TACCGAGATGGTTTATTGCTCAACATTCTTCAGGTACTTAAGGAGCTGGGTATAGAGACCACTGCAGTTCACACCGCCGTGAACGACCATGATTTTGAGGCAGAGATAAGGGCGAAAGTG
RsWf_2      TACCGAGATGGTTTATTGCTCAACATTCTTCAGGTACTTAAGGAGCTGGGTATAGAGACCACTGCAGTTCACACCGCCGTGAACGACCATGATTTTGAGGCAGAGATAAGGGCGAAAGTG
WsWf_1      TACCGAGATGGTTTATTGCTCAACATTCTTCAGGTACTTAAGGAGCTGGGTATAGAGACCACTGCAGTTCACACCGCCGTGAACGACCATGATTTTGAGGCAGAGATAAGGGCGAAAGTG
WsWf_2      TACCGAGATGGTTTATTGCTCAACATTCTTCAGGTACTTAAGGAGCTGGGTATAGAGACCACTGCAGTTCACACCGCCGTGAACGACCATGATTTTGAGGCAGAGATAAGGGCGAAAGTG
            ************************************************************************************************************************

GsRf_1      AGAGGGAAGAAACCAACCATTGCTGAGGTTAAAATAGCCATCCATCAAATCATATCTCAAAATAAACTCTAG  3965
RsRf_2      AGAGGGAAGAAACCAACCATTGCTGAGGTTAAAATAGCCATCCATCAAATCATATCTCAAAATAAACTCTAG  3967
RsRf_1      AGAGGGAAGAAACCAACCATTGCTGAGGTTAAAATAGCCATCCATCAAATCATATCTCAAAATAAACTCTAG  3961
GsRf_2      AGAGGGAAGAAACCAACCATTGCTGAGGTTAAAATAGCCATCCATCAAATCATATCTCAAAATAAACTCTAG  3961
RsWf_1      AGAGGGAAGAAACCAACCATTGCTGAGGTTAAAATAGCCATCCATCAAATCATATCTCAAAATAAACTCTAG  3934
RsWf_2      AGAGGGAAGAAACCAACCATTGCTGAGGTTAAAATAGCCATCCATCAAATCATATCTCAAAATAAACTCTAG  3934
WsWf_1      AGAGGGAAGAAACCAACCATTGCTGAGGTTAAAATAGCCATCCATCAAATCATATCTCAAAATAAACTCTAG  4041
WsWf_2      AGAGGGAAGAAACCAACCATTGCTGAGGTTAAAATAGCCATCCATCAAATCATATCTCAAAATAAACTCTAG  4039
            ************************************************************************
```

Yellow: cis-acting sequence

Red: SNP or InDel between Rf and Wf

Blue: WsWf specific SNP or InDel

Green: RsWF specific SNP or InDel

Brown: primer sequences

Red letter: key nucleotide for domain

Numbers: given from ATG start codon

MRE: MYB-recognition motif, BRE: bHLH-recognition motif

Cis-elements on promoter were not different from each othe. Therefore, we should examine expression of transcription factors that might form different MBW complex to regulate the TT8 expression, such as MYB5, GL3, EGL3, TTG1, TTG2, TT1 and TT16.

>RsWf_1
ATGGATGAATCAAGTATTATACCGGTATGGAAAGTGATCGGAGCTGAGGGAAAAGAGATTCAAGGGCTACTTAAGGCGGTGGTGCAATCTGTGGGGTGGACTTATAGTCTCTTCTGGCAACTTT
GTCCTCAACGAAGGAAATTGGTGTGGAGTAGTGGATTCTACAACGGTGCAATAAAGACTAGAAAGACAACTCAGCCGGCGGAAATAACGGCTGAAGAGGCTGCGTTGGAGAGAAGCCAACAGCT
CATGGAGCTTTACCAGACGCTTTTTGCCGGAGAATCATCGATGGAAGCGAGGGCTTCCACAGCACTGTCGCCTGAGGATTTGACGGACACTGAATGGTTTTATGTGCTGTGTCTCACTTACTCT
TTTGAACCTCCTTCTGGGATGCCAGGAAAGGCGTATGCGAGGAGGAAGCAAGTATGGATGAGTGGTGTAAATGAGGTTGACAGTAAAATCTTCTCTAGGGCTATTTCTGCAAAGAGTGCCAAAA
TTCAGACAGTGGTTTGCATTCCCGTGCTTGATGGCGTTTTGGAAATAGGCACAACGAACAAGGTCAAAGAAAATGAAGAGTTTGTTGAACACATAAAGAGTTTCTTCCAAAACCACCCGAAGTC
AAACACGAAGCCTGCTCTTTTTGAACACTCCATCAACGAAGATCATGAAGAAGACGAAGAAGAAGTAGAAGAAATGACAATGTCAGAGGAGATAAGACTTGGTTCTCCTGATGACGATGACGTC
TCCAATCAAAATCTACTCTCTGATTTCCATATAGAAGCACCCAATAGTTTAGATACACAAATGGACATGATGAATCTAATGGAGGAAGGCGGAAATTATTCTCAGACAGTATCAACACTTCTCA
TGTCACAACTCCCCAATCTTCTTTCAGATTCAGTTTCCACATCTTCTTACGTTCAATCATCGTTTGTCTCGTGGAGGGTTGAGAATGTCAAAGAGCATCAGCAATATCAACGAGAGGAGAAAGC
GTCGTCGTCATCCTCGTCGCAATGGATGCTCAAACACATGATCTTGAGAGTTCCTTTACTCCATGAAAACACTAAAAACAAGAGGGTGCCGCGGGAAGAGCTCAACCATGTGGTGGCCGAGCGA
CGCAGAAGAGAGAAGCTTAACGAGAGATTCATAACGTTGAGATCATTGGTTCCATTTGTGACCAAGATGGATAAAGTCTCGATCCTTGGAGACACCATTGATTACGTAAACCATCTTTGTAAGA
GGATCCATGAGCTGGAATCTACTCATCACGAGCCAAACCAAAAGCGGATGCGTATCGGTAAGGGAAGAACGTGGGAAGAGGTGGAGGTTTCCATTATAGAGAGCGATGTTTTGTTAGAGATGAG
ATGCGAGTACCGAGATGGTTTATTGCTCAACATTCTTCAGGTACTTAAGGAGCTGGGTATAGAGACCACTGCAGTTCACACCGCCGTGAACGACCATGATTTTGAGGCAGAGATAAGGGCGAAA
GTGAGAGGGAAGAAACCAACCATTGCTGAGGTTAAAATAGCCATCCATCAAATCATATCTCAAAATAAACTCTAG

>RsWf_2
ATGGATGAATCAAGTATTATACCGGTATGGAAAGTGATCGGAGCTGAGGGAAAAGAGATTCAAGGGCTACTTAAGGCGGTGGTGCAATCTGTGGGGTGGACTTATAGTCTCTTCTGGCAACTTT
GTCCTCAACGAAGGAAATTGGTGTGGAGTAGTGGATTCTACAACGGTGCAATAAAGACTAGAAAGACAACTCAGCCGGCGGAAATAACGGCTGAAGAGGCTGCGTTGGAGAGAAGCCAACAGCT
CATGGAGCTTTACCAGACGCTTTTTGCCGGAGAATCATCGATGGAAGCGAGGGCTTCCACAGCACTGTCGCCTGAGGATTTGACGGACACTGAATGGTTTTATGTGCTGTGTCTCACTTACTCT
TTTGAACCTCCTTCTGGGATGCCAGGAAAGGCGTATGCGAGGAGGAAGCAAGTATGGATGAGTGGTGTAAATGAGGTTGACAGTAAAATCTTCTCTAGGGCTATTTCTGCAAAGAGTGCCAAAA
TTCAGACAGTGGTTTGCATTCCCGTGCTTGATGGCGTTTTGGAAATAGGCACAACGAACAAGGTCAAAGAAAATGAAGAGTTTGTTGAACACATAAAGAGTTTCTTCCAAAACCACCCGAAGTC
AAACACGAAGCCTGCTCTTTTTGAACACTCCATCAACGAAGATCATGAAGAAGACGAAGAAGAAGTAGAAGAAATGACAATGTCAGAGGAGATAAGACTTGGTTCTCCTGATGACGATGACGTC
TCCAATCAAAATCTACTCTCTGATTTCCATATAGAAGCACCCAATAGTTTAGATACACAAATGGACATGATGAATCTAATGGAGGAAGGCGGAAATTATTCTCAGACAGTATCAACACTTCTCA
TGTCACAACTCCCCAATCTTCTTTCAGATTCAGTTTCCACATCTTCTTACGTTCAATCATCGTTTGTCTCGTGGAGGGTTGAGAATGTCAAAGAGCATCAGCAATATCAACGAGAGGAGAAAGC
GTCGTCGTCATCCTCGTCGCAATGGATGCTCAAACACATGATCTTGAGAGTTCCTTTACTCCATGAAAACACTAAAAACAAGAGGGTGCCGCGGGAAGAGCTCAACCATGTGGTGGCCGAGCGA
CGCAGAAGAGAGAAGCTTAACGAGAGATTCATAACGTTGAGATCATTGGTTCCATTTGTGACCAAGATGGATAAAGTCTCGATCCTTGGAGACACCATTGATTACGTAAACCATCTTTGTAAGA
GGATCCATGAGCTGGAATCTACTCATCACGAGCCAAACCAAAAGCGGATGCGTATCGGTAAGGGAAGAACGTGGGAAGAGGTGGAGGTTTCCATTATAGAGAGCGATGTTTTGTTAGAGATGAG
ATGCGAGTACCGAGATGGTTTATTGCTCAACATTCTTCAGGTACTTAAGGAGCTGGGTATAGAGACCACTGCAGTTCACACCGCCGTGAACGACCATGATTTTGAGGCAGAGATAAGGGCGAAA
GTGAGAGGGAAGAAACCAACCATTGCTGAGGTTAAAATAGCCATCCATCAAATCATATCTCAAAATAAACTCTAG

>GsRf_1
ATGGATGAATCAAGTATTATACCGGTATGGAAAGTGATCGGAGCTGAGGAAAAAGAGATTCAAGGGCTACTTAAGGCGGTGGTGCAATCTGTGGGGTGGACTTATAGTCTCTTCTGGCAACTTT
GTCCTCAACGAAGGAAATTGGTGTGGAGTAGTGGATTCTACAACGGTGCAATAAAGACTAGAAAGACAACTCAGCCGGCGGAAATAACGGCTGAAGAGGCTGCGTTGGAGAGAAGCCAACAGCT
CATGGAGCTTTACCAGACGCTTTTTGCCGGAGAATCATCGATGGAAGCGAGGGCTTGCACAGCACTGTCGCCTGAGGATTTGACGGACACTGAATGGTTTTATGTGCTGTGTCTCACTTACTCT
TTTGAACCTCCTTCTGGGATGCCAGGAAAGGCGTATGCGAGGAGGAAGCAAGTATGGATGAGTGGTGTAAATGAGGTTGACAGTAAAATCTTCTCTAGGGCTATTTCTGCAAAGAGTGCCAAAA
TTCAGACAGTGGTTTGCATTCCCGTGCTTGATGGCGTTTTGGAAATAGGCACAACGAACAAGGTCAAAGAAAATGAAGAGTTTGTTGAACACATGAAGAGTTTCTTCCAAAACCACCCGAAGTC
AAACACGAAGCCTGCTCTTTTTGAACACTCCATCAACGAAGAGCATGAAGAAGACGAAGAAGTAGAAGAAATGACAATGTCAGAGGAGATAAGACTTGGTTCTCCTGATGACGATGACGTCTCC
AATCAAAATCTACTCTCTGATTTCCATATAGAAGCACCCAGTAGTTTAGATACACAAATGGACATGATGAATCTAATGGAGGAAGGCGGAAATTATTCTCAGACAGTATCAACACTTCTCATGT
CACAACTCCCCAATCTTCTTTCAGATTCAGTTTCCACATCTTCTTACGTTCAATCATCGTTTGTCTCGTGGAGGGTTGAGAATGTCAAAGAGCATCAGCAATATCAACGAGAGGAGAAAGCGTC
GTCGTCATCCTCGTCGCAATGGATGCTCAAACACATGATCTTGAGAGTTCCTTTACTCCATGAAAACACTAAAAACAAGAGGGTGCCGCGGGAAGAGCTCAACCATGTGGTGGCCGAGCGACGC
AGAAGAGAGAAGCTTAACGAGAGATTCATAACGTTGAGATCATTGGTTCCATTTGTGACCAAGATGGATAAAGTCTCGATCCTTGGAGACACCATTGATTACGTAAACCATCTTTGTAAGAGGA
TCCATGAGCTGGAATCTACTCATCACGAGCCAAACCAAAAGCGGATGCGTATCGGTAAGGGAAGAACGTGGGAAGAGGTGGAGGTTTCCATTATAGAGAGCGATGTTTTGTTAGAGATGAGATG
CGAGTACCGAGATGGTTTATTGCTCAACATTCTTCAGGTACTTAAGGAGCTGGGTATAGAGACCACTGCAGTTCACACCGCCGTGAACGACCATGATTTTGAGGCAGAGATAAGGGCGAAAGTG
AGAGGGAAGAAACCAACCATTGCTGAGGTTAAAATAGCCATCCATCAAATCATATCTCAAAATAAACTCTAG

>GsRf_2
ATGGATGAATCAAGTATTATACCGGTATGGAAAGTGATCGGAGCTGAGGAAAAAGAGATTCAAGGGCTACTTAAGGCGGTGGTGCAATCTGTGGGGTGGACTTATAGTCTCTTCTGGCAACTTT
GTCCTCAACGAAGGAAATTGGTGTGGAGTAGTGGATTCTACAACGGTGCAATAAAGACTAGAAAGACAACTCAGCCGGCGGAAATAACGGCTGAAGAGGCTGCGTTGGAGAGAAGCCAACAGCT
CATGGAGCTTTACCAGACGCTTTTTGCCGGAGAATCATCGATGGAAGCGAGGGCTTGCACAGCACTGTCGCCTGAGGATTTGACGGACACTGAATGGTTTTATGTGCTGTGTCTCACTTACTCT
TTTGAACCTCCTTCTGGGATGCCAGGAAAGGCGTATGCGAGGAGGAAGCAAGTATGGATGAGTGGTGTAAATGAGGTTGACAGTAAAATCTTCTCTAGGGCTATTTCTGCAAAGAGTGCCAAAA
TTCAGACAGTGGTTTGCATTCCCGTGCTTGATGGCGTTTTGGAAATAGGCACAACGAACAAGGTCAAAGAAAATGAAGAGTTTGTTGAACACATGAAGAGTTTCTTCCAAAACCACCCGAAGTC
AAACACGAAGCCTGCTCTTTTTGAACACTCCATCAACGAAGAGCATGAAGAAGACGAAGAAGTAGAAGAAATGACAATGTCAGAGGAGATAAGACTTGGTTCTCCTGATGACGATGACGTCTCC
AATCAAAATCTACTCTCTGATTTCCATATAGAAGCACCCAGTAGTTTAGATACACAAATGGACATGATGAATCTAATGGAGGAAGGCGGAAATTATTCTCAGACAGTATCAACACTTCTCATGT
CACAACTCCCCAATCTTCTTTCAGATTCAGTTTCCACATCTTCTTACGTTCAATCATCGTTTGTCTCGTGGAGGGTTGAGAATGTCAAAGAGCATCAGCAATATCAACGAGAGGAGAAAGCGTC
GTCGTCATCCTCGTCGCAATGGATGCTCAAACACATGATCTTGAGAGTTCCTTTACTCCATGAAAACACTAAAAACAAGAGGGTGCCGCGGGAAGAGCTCAACCATGTGGTGGCCGAGCGACGC
AGAAGAGAGAAGCTTAACGAGAGATTCATAACGTTGAGATCATTGGTTCCATTTGTGACCAAGATGGATAAAGTCTCGATCCTTGGAGACACCATTGATTACGTAAACCATCTTTGTAAGAGGA
TCCATGAGCTGGAATCTACTCATCACGAGCCAAACCAAAAGCGGATGCGTATCGGTAAGGGAAGAACGTGGGAAGAGGTGGAGGTTTCCATTATAGAGAGCGATGTTTTGTTAGAGATGAGATG
CGAGTACCGAGATGGTTTATTGCTCAACATTCTTCAGGTACTTAAGGAGCTGGGTATAGAGACCACTGCAGTTCACACCGCCGTGAACGACCATGATTTTGAGGCAGAGATAAGGGCGAAAGTG
AGAGGGAAGAAACCAACCATTGCTGAGGTTAAAATAGCCATCCATCAAATCATATCTCAAAATAAACTCTAG

>RsRf_1
ATGGATGAATCAAGTATTATACCGGTATGGAAAGTGATCGGAGCTGAGGAAAAAGAGATTCAAGGGCTACTTAAGGCGGTGGTGCAATCTGTGGGGTGGACTTATAGTCTCTTCTGGCAACTTT
GTCCTCAACGAAGGAAATTGGTGTGGAGTAGTGGATTCTACAACGGTGCAATAAAGACTAGAAAGACAACTCAGCCGGCGGAAATAACGGCTGAAGAGGCTGCGTTGGAGAGAAGCCAACAGCT
CATGGAGCTTTACCAGACGCTTTTTGCCGGAGAATCATCGATGGAAGCGAGGGCTTGCACAGCACTGTCGCCTGAGGATTTGACGGACACTGAATGGTTTTATGTGCTGTGTCTCACTTACTCT
TTTGAACCTCCTTCTGGGATGCCAGGAAAGGCGTATGCGAGGAGGAAGCAAGTATGGATGAGTGGTGTAAATGAGGTTGACAGTAAAATCTTCTCTAGGGCTATTTCTGCAAAGAGTGCCAAAA
TTCAGACAGTGGTTTGCATTCCCGTGCTTGATGGCGTTTTGGAAATAGGCACAACGAACAAGGTCAAAGAAAATGAAGAGTTTGTTGAACACATGAAGAGTTTCTTCCAAAACCACCCGAAGTC
AAACACGAAGCCTGCTCTTTTTGAACACTCCATCAACGAAGAGCATGAAGAAGACGAAGAAGTAGAAGAAATGACAATGTCAGAGGAGATAAGACTTGGTTCTCCTGATGACGATGACGTCTCC
AATCAAAATCTACTCTCTGATTTCCATATAGAAGCACCCAGTAGTTTAGATACACAAATGGACATGATGAATCTAATGGAGGAAGGCGGAAATTATTCTCAGACAGTATCAACACTTCTCATGT
CACAACTCCCCAATCTTCTTTCAGATTCAGTTTCCACATCTTCTTACGTTCAATCATCGTTTGTCTCGTGGAGGGTTGAGAATGTCAAAGAGCATCAGCAATATCAACGAGAGGAGAAAGCGTC
GTCGTCATCCTCGTCGCAATGGATGCTCAAACACATGATCTTGAGAGTTCCTTTACTCCATGAAAACACTAAAAACAAGAGGGTGCCGCGGGAAGAGCTCAACCATGTGGTGGCCGAGCGACGC
AGAAGAGAGAAGCTTAACGAGAGATTCATAACGTTGAGATCATTGGTTCCATTTGTGACCAAGATGGATAAAGTCTCGATCCTTGGAGACACCATTGATTACGTAAACCATCTTTGTAAGAGGA
TCCATGAGCTGGAATCTACTCATCACGAGCCAAACCAAAAGCGGATGCGTATCGGTAAGGGAAGAACGTGGGAAGAGGTGGAGGTTTCCATTATAGAGAGCGATGTTTTGTTAGAGATGAGATG
CGAGTACCGAGATGGTTTATTGCTCAACATTCTTCAGGTACTTAAGGAGCTGGGTATAGAGACCACTGCAGTTCACACCGCCGTGAACGACCATGATTTTGAGGCAGAGATAAGGGCGAAAGTG
AGAGGGAAGAAACCAACCATTGCTGAGGTTAAAATAGCCATCCATCAAATCATATCTCAAAATAAACTCTAG

>RsRf_2
ATGGATGAATCAAGTATTATACCGGTATGGAAAGTGATCGGAGCTGAGGAAAAAGAGATTCAAGGGCTACTTAAGGCGGTGGTGCAATCTGTGGGGTGGACTTATAGTCTCTTCTGGCAACTTT
GTCCTCAACGAAGGAAATTGGTGTGGAGTAGTGGATTCTACAACGGTGCAATAAAGACTAGAAAGACAACTCAGCCGGCGGAAATAACGGCTGAAGAGGCTGCGTTGGAGAGAAGCCAACAGCT
CATGGAGCTTTACCAGACGCTTTTTGCCGGAGAATCATCGATGGAAGCGAGGGCTTGCACAGCACTGTCGCCTGAGGATTTGACGGACACTGAATGGTTTTATGTGCTGTGTCTCACTTACTCT
TTTGAACCTCCTTCTGGGATGCCAGGAAAGGCGTATGCGAGGAGGAAGCAAGTATGGATGAGTGGTGTAAATGAGGTTGACAGTAAAATCTTCTCTAGGGCTATTTCTGCAAAGAGTGCCAAAA
TTCAGACAGTGGTTTGCATTCCCGTGCTTGATGGCGTTTTGGAAATAGGCACAACGAACAAGGTCAAAGAAAATGAAGAGTTTGTTGAACACATGAAGAGTTTCTTCCAAAACCACCCGAAGTC
AAACACGAAGCCTGCTCTTTTTGAACACTCCATCAACGAAGAGCATGAAGAAGACGAAGAAGTAGAAGAAATGACAATGTCAGAGGAGATAAGACTTGGTTCTCCTGATGACGATGACGTCTCC
AATCAAAATCTACTCTCTGATTTCCATATAGAAGCACCCAGTAGTTTAGATACACAAATGGACATGATGAATCTAATGGAGGAAGGCGGAAATTATTCTCAGACAGTATCAACACTTCTCATGT
CACAACTCCCCAATCTTCTTTCAGATTCAGTTTCCACATCTTCTTACGTTCAATCATCGTTTGTCTCGTGGAGGGTTGAGAATGTCAAAGAGCATCAGCAATATCAACGAGAGGAGAAAGCGTC
GTCGTCATCCTCGTCGCAATGGATGCTCAAACACATGATCTTGAGAGTTCCTTTACTCCATGAAAACACTAAAAACAAGAGGGTGCCGCGGGAAGAGCTCAACCATGTGGTGGCCGAGCGACGC

```
AGAAGAGAGAAGCTTAACGAGAGATTCATAACGTTGAGATCATTGGTTCCATTTGTGACCAAGATGGATAAAGTCTCGATCCTTGGAGACACCATTGATTACGTAAACCATCTTTGTAAGAGGA
TCCATGAGCTGGAATCTACTCATCACGAGCCAAACCAAAAGCGGATGCGTATCGGTAAGGGAAGAACGTGGGAAGAGGTGGAGGTTTCCATTATAGAGAGCGATGTTTTGTTAGAGATGAGATG
CGAGTACCGAGATGGTTTATTGCTCAACATTCTTCAGGTACTTAAGGAGCTGGGTATAGAGACCACTGCAGTTCACACCGCCGTGAACGACCATGATTTTGAGGCAGAGATAAGGGCGAAAGTG
AGAGGGAAGAAACCAACCATTGCTGAGGTTAAAATAGCCATCCATCAAATCATATCTCAAAATAAACTCTAG

>WsWf_1
ATGGATGAATCAAGTATTATACCGGTATGGAAAGTGATCGGAGCTGAGGAAAAAGAGATTCAAGGGCTACTTAAGGCGGTGGTGCAATCTGTGGGGTGGACTTATAGTCTCTTCTGGCAACTTT
GTCCTCAACGAAGGAAATTGGTGTGGAGTAGTGGATTCTACAACGGTGCAATAAAGACTAGAAAGACAACTCAGCCGGCGGAAATAACGGCTGAAGAGGCTGCGTTGGAGAGAAGCCAACAGCT
CATGGAGCTTTACCAGACGCTTTTTGCCGGAGAATCATCGATGGAAGCGAGGGCTTGCACAGCACTGTCGCCTGAGGATTTGACGGACACTGAATGGTTTTATGTGCTGTGTCTCACTTACTCT
TTTGAACCTCCTTCTGGGATGCCAGGAAAGGCGTATGCGAGGAGGAAGCAAGTATGGATGAGTGGTGTAAATGAGGTTGACAGTAAAATCTTCTCTAGGGCTATTTCTGCAAAGAGTGCCAAAA
TTCAGACAGTGGTTTGCATTCCCGTGCTTGATGGCGTTTTGGAAATAGGCACAACGAACAAGGTCAAAGAAAATGAAGAGTTTGTTGAACACATAAAGAGTTTCTTCCAAAACCACCCGAAGTC
AAACACGAAGCCTGCTCTTTTTGAACACTCCATCAACGAAGAGCATGAAGAAGACGAAGAAGTAGAAGAAATGACAATGTCAGAGGAGATAAGACTTGGTTCTCCTGATGACGATGACGTCTCC
AATCAAAATCTACTCTCTGATTTCCATATAGAAGCACCCAATAGTTTAGATACACAAATGGACATGATGAATCTAATGGAGGAAGGCGGAAGTTATTCTCAGACAGTATCAACACTTCTCATGT
CACAACTCCCCAATCTTCTTTCAGATTCAGTTTCCACATCTTCTTACGTTCAATCATCGTTTGTCTCGTGGAGGGTTGAGAATGTCAAAGAGCATCAGCAATATCAACGAGAGGAGAAAGCGTC
GTCGTCATCCTCGTCGCAATGGATGCTCAAACACATGATCTTGAGAGTTCCTTTACTCCATGAAAACACTAAAAACAAGAGGGTGCCGCGGGAAGAGCTCAACCATGTGGTGGCCGAGCGACGC
AGAAGAGAGAAGCTTAACGAGAGATTCATAACGTTGAGATCATTGGTTCCATTTGTGACCAAGATGGATAAAGTCTCGATCCTTGGAGACACCATTGATTACGTAAACCATCTTTGTAAGAGGA
TCCATGAGCTGGAATCTACTCATCACGAGCCAAACCAAAAGCGGATGCGTATCGGTAAGGGAAGAACGTGGGAAGAGGTGGAGGTTTCCATTATAGAGAGCGATGTTTTGTTAGAGATGAGATG
CGAGTACCGAGATGGTTTATTGCTCAACATTCTTCAGGTACTTAAGGAGCTGGGTATAGAGACCACTGCAGTTCACACCGCCGTGAACGACCATGATTTTGAGGCAGAGATAAGGGCGAAAGTG
AGAGGGAAGAAACCAACCATTGCTGAGGTTAAAATAGCCATCCATCAAATCATATCTCAAAATAAACTCTAG

>WsWf_2
ATGGATGAATCAAGTATTATACCGGTATGGAAAGTGATCGGAGCTGAGGAAAAAGAGATTCAAGGGCTACTTAAGGCGGTGGTGCAATCTGTGGGGTGGACTTATAGTCTCTTCTGGCAACTTT
GTCCTCAACGAAGGAAATTGGTGTGGAGTAGTGGATTCTACAACGGTGCAATAAAGACTAGAAAGACAACTCAGCCGGCGGAAATAACGGCTGAAGAGGCTGCGTTGGAGAGAAGCCAACAGCT
CATGGAGCTTTACCAGACGCTTTTTGCCGGAGAATCATCGATGGAAGCGAGGGCTTGCACAGCACTGTCGCCTGAGGATTTGACGGACACTGAATGGTTTTATGTGCTGTGTCTCACTTACTCT
TTTGAACCTCCTTCTGGGATGCCAGGAAAGGCGTATGCGAGGAGGAAGCAAGTATGGATGAGTGGTGTAAATGAGGTTGACAGTAAAATCTTCTCTAGGGCTATTTCTGCAAAGAGTGCCAAAA
TTCAGACAGTGGTTTGCATTCCCGTGCTTGATGGCGTTTTGGAAATAGGCACAACGAACAAGGTCAAAGAAAATGAAGAGTTTGTTGAACACATAAAGAGTTTCTTCCAAAACCACCCGAAGTC
AAACACGAAGCCTGCTCTTTTTGAACACTCCATCAACGAAGAGCATGAAGAAGACGAAGAAGTAGAAGAAATGACAATGTCAGAGGAGATAAGACTTGGTTCTCCTGATGACGATGACGTCTCC
AATCAAAATCTACTCTCTGATTTCCATATAGAAGCACCCAATAGTTTAGATACACAAATGGACATGATGAATCTAATGGAGGAAGGCGGAAGTTATTCTCAGACAGTATCAACACTTCTCATGT
CACAACTCCCCAATCTTCTTTCAGATTCAGTTTCCACATCTTCTTACGTTCAATCATCGTTTGTCTCGTGGAGGGTTGAGAATGTCAAAGAGCATCAGCAATATCAACGAGAGGAGAAAGCGTC
GTCGTCATCCTCGTCGCAATGGATGCTCAAACACATGATCTTGAGAGTTCCTTTACTCCATGAAAACACTAAAAACAAGAGGGTGCCGCGGGAAGAGCTCAACCATGTGGTGGCCGAGCGACGC
AGAAGAGAGAAGCTTAACGAGAGATTCATAACGTTGAGATCATTGGTTCCATTTGTGACCAAGATGGATAAAGTCTCGATCCTTGGAGACACCATTGATTACGTAAACCATCTTTGTAAGAGGA
TCCATGAGCTGGAATCTACTCATCACGAGCCAAACCAAAAGCGGATGCGTATCGGTAAGGGAAGAACGTGGGAAGAGGTGGAGGTTTCCATTATAGAGAGCGATGTTTTGTTAGAGATGAGATG
CGAGTACCGAGATGGTTTATTGCTCAACATTCTTCAGGTACTTAAGGAGCTGGGTATAGAGACCACTGCAGTTCACACCGCCGTGAACGACCATGATTTTGAGGCAGAGATAAGGGCGAAAGTG
AGAGGGAAGAAACCAACCATTGCTGAGGTTAAAATAGCCATCCATCAAATCATATCTCAAAATAAACTCTAG
```

```
RsRf_1          ATGGATGAATCAAGTATTATACCGGTATGGAAAGTGATCGGAGCTGAGGAAAAAGAGATT
RsRf_2          ATGGATGAATCAAGTATTATACCGGTATGGAAAGTGATCGGAGCTGAGGAAAAAGAGATT
GsRf_2          ATGGATGAATCAAGTATTATACCGGTATGGAAAGTGATCGGAGCTGAGGAAAAAGAGATT
GsRf_1          ATGGATGAATCAAGTATTATACCGGTATGGAAAGTGATCGGAGCTGAGGAAAAAGAGATT
WsWf_1          ATGGATGAATCAAGTATTATACCGGTATGGAAAGTGATCGGAGCTGAGGAAAAAGAGATT
WsWf_2          ATGGATGAATCAAGTATTATACCGGTATGGAAAGTGATCGGAGCTGAGGAAAAAGAGATT
RsWf_1          ATGGATGAATCAAGTATTATACCGGTATGGAAAGTGATCGGAGCTGAGGGAAAAGAGATT
RsWf_2          ATGGATGAATCAAGTATTATACCGGTATGGAAAGTGATCGGAGCTGAGGGAAAAGAGATT
                ************************************************* **********

RsRf_1          CAAGGGCTACTTAAGGCGGTGGTGCAATCTGTGGGGTGGACTTATAGTCTCTTCTGGCAA
RsRf_2          CAAGGGCTACTTAAGGCGGTGGTGCAATCTGTGGGGTGGACTTATAGTCTCTTCTGGCAA
GsRf_2          CAAGGGCTACTTAAGGCGGTGGTGCAATCTGTGGGGTGGACTTATAGTCTCTTCTGGCAA
GsRf_1          CAAGGGCTACTTAAGGCGGTGGTGCAATCTGTGGGGTGGACTTATAGTCTCTTCTGGCAA
WsWf_1          CAAGGGCTACTTAAGGCGGTGGTGCAATCTGTGGGGTGGACTTATAGTCTCTTCTGGCAA
WsWf_2          CAAGGGCTACTTAAGGCGGTGGTGCAATCTGTGGGGTGGACTTATAGTCTCTTCTGGCAA
RsWf_1          CAAGGGCTACTTAAGGCGGTGGTGCAATCTGTGGGGTGGACTTATAGTCTCTTCTGGCAA
RsWf_2          CAAGGGCTACTTAAGGCGGTGGTGCAATCTGTGGGGTGGACTTATAGTCTCTTCTGGCAA
                ************************************************************

RsRf_1          CTTTGTCCTCAACGAAGGAAATTGGTGTGGAGTAGTGGATTCTACAACGGTGCAATAAAG
RsRf_2          CTTTGTCCTCAACGAAGGAAATTGGTGTGGAGTAGTGGATTCTACAACGGTGCAATAAAG
GsRf_2          CTTTGTCCTCAACGAAGGAAATTGGTGTGGAGTAGTGGATTCTACAACGGTGCAATAAAG
GsRf_1          CTTTGTCCTCAACGAAGGAAATTGGTGTGGAGTAGTGGATTCTACAACGGTGCAATAAAG
WsWf_1          CTTTGTCCTCAACGAAGGAAATTGGTGTGGAGTAGTGGATTCTACAACGGTGCAATAAAG
WsWf_2          CTTTGTCCTCAACGAAGGAAATTGGTGTGGAGTAGTGGATTCTACAACGGTGCAATAAAG
RsWf_1          CTTTGTCCTCAACGAAGGAAATTGGTGTGGAGTAGTGGATTCTACAACGGTGCAATAAAG
RsWf_2          CTTTGTCCTCAACGAAGGAAATTGGTGTGGAGTAGTGGATTCTACAACGGTGCAATAAAG
                ************************************************************

RsRf_1          ACTAGAAAGACAACTCAGCCGGCGGAAATAACGGCTGAAGAGGCTGCGTTGGAGAGAAGC
RsRf_2          ACTAGAAAGACAACTCAGCCGGCGGAAATAACGGCTGAAGAGGCTGCGTTGGAGAGAAGC
GsRf_2          ACTAGAAAGACAACTCAGCCGGCGGAAATAACGGCTGAAGAGGCTGCGTTGGAGAGAAGC
GsRf_1          ACTAGAAAGACAACTCAGCCGGCGGAAATAACGGCTGAAGAGGCTGCGTTGGAGAGAAGC
WsWf_1          ACTAGAAAGACAACTCAGCCGGCGGAAATAACGGCTGAAGAGGCTGCGTTGGAGAGAAGC
WsWf_2          ACTAGAAAGACAACTCAGCCGGCGGAAATAACGGCTGAAGAGGCTGCGTTGGAGAGAAGC
RsWf_1          ACTAGAAAGACAACTCAGCCGGCGGAAATAACGGCTGAAGAGGCTGCGTTGGAGAGAAGC
RsWf_2          ACTAGAAAGACAACTCAGCCGGCGGAAATAACGGCTGAAGAGGCTGCGTTGGAGAGAAGC
                ************************************************************

RsRf_1          CAACAGCTCATGGAGCTTTACCAGACGCTTTTTGCCGGAGAATCATCGATGGAAGCGAGG
RsRf_2          CAACAGCTCATGGAGCTTTACCAGACGCTTTTTGCCGGAGAATCATCGATGGAAGCGAGG
GsRf_2          CAACAGCTCATGGAGCTTTACCAGACGCTTTTTGCCGGAGAATCATCGATGGAAGCGAGG
GsRf_1          CAACAGCTCATGGAGCTTTACCAGACGCTTTTTGCCGGAGAATCATCGATGGAAGCGAGG
WsWf_1          CAACAGCTCATGGAGCTTTACCAGACGCTTTTTGCCGGAGAATCATCGATGGAAGCGAGG
WsWf_2          CAACAGCTCATGGAGCTTTACCAGACGCTTTTTGCCGGAGAATCATCGATGGAAGCGAGG
RsWf_1          CAACAGCTCATGGAGCTTTACCAGACGCTTTTTGCCGGAGAATCATCGATGGAAGCGAGG
RsWf_2          CAACAGCTCATGGAGCTTTACCAGACGCTTTTTGCCGGAGAATCATCGATGGAAGCGAGG
                ************************************************************

RsRf_1          GCTTGCACAGCACTGTCGCCTGAGGATTTGACGGACACTGAATGGTTTTATGTGCTGTGT
RsRf_2          GCTTGCACAGCACTGTCGCCTGAGGATTTGACGGACACTGAATGGTTTTATGTGCTGTGT
GsRf_2          GCTTGCACAGCACTGTCGCCTGAGGATTTGACGGACACTGAATGGTTTTATGTGCTGTGT
GsRf_1          GCTTGCACAGCACTGTCGCCTGAGGATTTGACGGACACTGAATGGTTTTATGTGCTGTGT
WsWf_1          GCTTGCACAGCACTGTCGCCTGAGGATTTGACGGACACTGAATGGTTTTATGTGCTGTGT
WsWf_2          GCTTGCACAGCACTGTCGCCTGAGGATTTGACGGACACTGAATGGTTTTATGTGCTGTGT
```

```
RsWf_1          GCTTCCACAGCACTGTCGCCTGAGGATTTGACGGACACTGAATGGTTTTATGTGCTGTGT
RsWf_2          GCTTCCACAGCACTGTCGCCTGAGGATTTGACGGACACTGAATGGTTTTATGTGCTGTGT
                **** *******************************************************

RsRf_1          CTCACTTACTCTTTTGAACCTCCTTCTGGGATGCCAGGAAAGGCGTATGCGAGGAGGAAG
RsRf_2          CTCACTTACTCTTTTGAACCTCCTTCTGGGATGCCAGGAAAGGCGTATGCGAGGAGGAAG
GsRf_2          CTCACTTACTCTTTTGAACCTCCTTCTGGGATGCCAGGAAAGGCGTATGCGAGGAGGAAG
GsRf_1          CTCACTTACTCTTTTGAACCTCCTTCTGGGATGCCAGGAAAGGCGTATGCGAGGAGGAAG
WsWf_1          CTCACTTACTCTTTTGAACCTCCTTCTGGGATGCCAGGAAAGGCGTATGCGAGGAGGAAG
WsWf_2          CTCACTTACTCTTTTGAACCTCCTTCTGGGATGCCAGGAAAGGCGTATGCGAGGAGGAAG
RsWf_1          CTCACTTACTCTTTTGAACCTCCTTCTGGGATGCCAGGAAAGGCGTATGCGAGGAGGAAG
RsWf_2          CTCACTTACTCTTTTGAACCTCCTTCTGGGATGCCAGGAAAGGCGTATGCGAGGAGGAAG
                ************************************************************

RsRf_1          CAAGTATGGATGAGTGGTGTAAATGAGGTTGACAGTAAAATCTTCTCTAGGGCTATTTCT
RsRf_2          CAAGTATGGATGAGTGGTGTAAATGAGGTTGACAGTAAAATCTTCTCTAGGGCTATTTCT
GsRf_2          CAAGTATGGATGAGTGGTGTAAATGAGGTTGACAGTAAAATCTTCTCTAGGGCTATTTCT
GsRf_1          CAAGTATGGATGAGTGGTGTAAATGAGGTTGACAGTAAAATCTTCTCTAGGGCTATTTCT
WsWf_1          CAAGTATGGATGAGTGGTGTAAATGAGGTTGACAGTAAAATCTTCTCTAGGGCTATTTCT
WsWf_2          CAAGTATGGATGAGTGGTGTAAATGAGGTTGACAGTAAAATCTTCTCTAGGGCTATTTCT
RsWf_1          CAAGTATGGATGAGTGGTGTAAATGAGGTTGACAGTAAAATCTTCTCTAGGGCTATTTCT
RsWf_2          CAAGTATGGATGAGTGGTGTAAATGAGGTTGACAGTAAAATCTTCTCTAGGGCTATTTCT
                ************************************************************

RsRf_1          GCAAAGAGTGCCAAAATTCAGACAGTGGTTTGCATTCCCGTGCTTGATGGCGTTTTGGAA
RsRf_2          GCAAAGAGTGCCAAAATTCAGACAGTGGTTTGCATTCCCGTGCTTGATGGCGTTTTGGAA
GsRf_2          GCAAAGAGTGCCAAAATTCAGACAGTGGTTTGCATTCCCGTGCTTGATGGCGTTTTGGAA
GsRf_1          GCAAAGAGTGCCAAAATTCAGACAGTGGTTTGCATTCCCGTGCTTGATGGCGTTTTGGAA
WsWf_1          GCAAAGAGTGCCAAAATTCAGACAGTGGTTTGCATTCCCGTGCTTGATGGCGTTTTGGAA
WsWf_2          GCAAAGAGTGCCAAAATTCAGACAGTGGTTTGCATTCCCGTGCTTGATGGCGTTTTGGAA
RsWf_1          GCAAAGAGTGCCAAAATTCAGACAGTGGTTTGCATTCCCGTGCTTGATGGCGTTTTGGAA
RsWf_2          GCAAAGAGTGCCAAAATTCAGACAGTGGTTTGCATTCCCGTGCTTGATGGCGTTTTGGAA
                ************************************************************

RsRf_1          ATAGGCACAACGAACAAGGTCAAAGAAAATGAAGAGTTTGTTGAACACATGAAGAGTTTC
RsRf_2          ATAGGCACAACGAACAAGGTCAAAGAAAATGAAGAGTTTGTTGAACACATGAAGAGTTTC
GsRf_2          ATAGGCACAACGAACAAGGTCAAAGAAAATGAAGAGTTTGTTGAACACATGAAGAGTTTC
GsRf_1          ATAGGCACAACGAACAAGGTCAAAGAAAATGAAGAGTTTGTTGAACACATGAAGAGTTTC
WsWf_1          ATAGGCACAACGAACAAGGTCAAAGAAAATGAAGAGTTTGTTGAACACATAAAGAGTTTC
WsWf_2          ATAGGCACAACGAACAAGGTCAAAGAAAATGAAGAGTTTGTTGAACACATAAAGAGTTTC
RsWf_1          ATAGGCACAACGAACAAGGTCAAAGAAAATGAAGAGTTTGTTGAACACATAAAGAGTTTC
RsWf_2          ATAGGCACAACGAACAAGGTCAAAGAAAATGAAGAGTTTGTTGAACACATAAAGAGTTTC
                ************************************************** *********

RsRf_1          TTCCAAAACCACCCGAAGTCAAACACGAAGCCTGCTCTTTTTGAACACTCCATCAACGAA
RsRf_2          TTCCAAAACCACCCGAAGTCAAACACGAAGCCTGCTCTTTTTGAACACTCCATCAACGAA
GsRf_2          TTCCAAAACCACCCGAAGTCAAACACGAAGCCTGCTCTTTTTGAACACTCCATCAACGAA
GsRf_1          TTCCAAAACCACCCGAAGTCAAACACGAAGCCTGCTCTTTTTGAACACTCCATCAACGAA
WsWf_1          TTCCAAAACCACCCGAAGTCAAACACGAAGCCTGCTCTTTTTGAACACTCCATCAACGAA
WsWf_2          TTCCAAAACCACCCGAAGTCAAACACGAAGCCTGCTCTTTTTGAACACTCCATCAACGAA
RsWf_1          TTCCAAAACCACCCGAAGTCAAACACGAAGCCTGCTCTTTTTGAACACTCCATCAACGAA
RsWf_2          TTCCAAAACCACCCGAAGTCAAACACGAAGCCTGCTCTTTTTGAACACTCCATCAACGAA
                ************************************************************

RsRf_1          GAGCATGAAGAAGACGAAGAAG---TAGAAGAAATGACAATGTCAGAGGAGATAAGACTT
RsRf_2          GAGCATGAAGAAGACGAAGAAG---TAGAAGAAATGACAATGTCAGAGGAGATAAGACTT
GsRf_2          GAGCATGAAGAAGACGAAGAAG---TAGAAGAAATGACAATGTCAGAGGAGATAAGACTT
GsRf_1          GAGCATGAAGAAGACGAAGAAG---TAGAAGAAATGACAATGTCAGAGGAGATAAGACTT
WsWf_1          GAGCATGAAGAAGACGAAGAAG---TAGAAGAAATGACAATGTCAGAGGAGATAAGACTT
WsWf_2          GAGCATGAAGAAGACGAAGAAG---TAGAAGAAATGACAATGTCAGAGGAGATAAGACTT
RsWf_1          GATCATGAAGAAGACGAAGAAGAAGTAGAAGAAATGACAATGTCAGAGGAGATAAGACTT
RsWf_2          GATCATGAAGAAGACGAAGAAGAAGTAGAAGAAATGACAATGTCAGAGGAGATAAGACTT
                ** *******************   ***********************************

RsRf_1          GGTTCTCCTGATGACGATGACGTCTCCAATCAAAATCTACTCTCTGATTTCCATATAGAA
RsRf_2          GGTTCTCCTGATGACGATGACGTCTCCAATCAAAATCTACTCTCTGATTTCCATATAGAA
GsRf_2          GGTTCTCCTGATGACGATGACGTCTCCAATCAAAATCTACTCTCTGATTTCCATATAGAA
GsRf_1          GGTTCTCCTGATGACGATGACGTCTCCAATCAAAATCTACTCTCTGATTTCCATATAGAA
WsWf_1          GGTTCTCCTGATGACGATGACGTCTCCAATCAAAATCTACTCTCTGATTTCCATATAGAA
WsWf_2          GGTTCTCCTGATGACGATGACGTCTCCAATCAAAATCTACTCTCTGATTTCCATATAGAA
RsWf_1          GGTTCTCCTGATGACGATGACGTCTCCAATCAAAATCTACTCTCTGATTTCCATATAGAA
RsWf_2          GGTTCTCCTGATGACGATGACGTCTCCAATCAAAATCTACTCTCTGATTTCCATATAGAA
                ************************************************************

RsRf_1          GCACCCAGTAGTTTAGATACACAAATGGACATGATGAATCTAATGGAGGAAGGCGGAAAT
RsRf_2          GCACCCAGTAGTTTAGATACACAAATGGACATGATGAATCTAATGGAGGAAGGCGGAAAT
GsRf_2          GCACCCAGTAGTTTAGATACACAAATGGACATGATGAATCTAATGGAGGAAGGCGGAAAT
GsRf_1          GCACCCAGTAGTTTAGATACACAAATGGACATGATGAATCTAATGGAGGAAGGCGGAAAT
WsWf_1          GCACCCAATAGTTTAGATACACAAATGGACATGATGAATCTAATGGAGGAAGGCGGAAGT
WsWf_2          GCACCCAATAGTTTAGATACACAAATGGACATGATGAATCTAATGGAGGAAGGCGGAAGT
RsWf_1          GCACCCAATAGTTTAGATACACAAATGGACATGATGAATCTAATGGAGGAAGGCGGAAAT
RsWf_2          GCACCCAATAGTTTAGATACACAAATGGACATGATGAATCTAATGGAGGAAGGCGGAAAT
                ******* ************************************************** *

RsRf_1          TATTCTCAGACAGTATCAACACTTCTCATGTCACAACTCCCCAATCTTCTTTCAGATTCA
RsRf_2          TATTCTCAGACAGTATCAACACTTCTCATGTCACAACTCCCCAATCTTCTTTCAGATTCA
GsRf_2          TATTCTCAGACAGTATCAACACTTCTCATGTCACAACTCCCCAATCTTCTTTCAGATTCA
GsRf_1          TATTCTCAGACAGTATCAACACTTCTCATGTCACAACTCCCCAATCTTCTTTCAGATTCA
WsWf_1          TATTCTCAGACAGTATCAACACTTCTCATGTCACAACTCCCCAATCTTCTTTCAGATTCA
WsWf_2          TATTCTCAGACAGTATCAACACTTCTCATGTCACAACTCCCCAATCTTCTTTCAGATTCA
RsWf_1          TATTCTCAGACAGTATCAACACTTCTCATGTCACAACTCCCCAATCTTCTTTCAGATTCA
RsWf_2          TATTCTCAGACAGTATCAACACTTCTCATGTCACAACTCCCCAATCTTCTTTCAGATTCA
                ************************************************************

RsRf_1          GTTTCCACATCTTCTTACGTTCAATCATCGTTTGTCTCGTGGAGGGTTGAGAATGTCAAA
RsRf_2          GTTTCCACATCTTCTTACGTTCAATCATCGTTTGTCTCGTGGAGGGTTGAGAATGTCAAA
GsRf_2          GTTTCCACATCTTCTTACGTTCAATCATCGTTTGTCTCGTGGAGGGTTGAGAATGTCAAA
GsRf_1          GTTTCCACATCTTCTTACGTTCAATCATCGTTTGTCTCGTGGAGGGTTGAGAATGTCAAA
```

```
WsWf_1          GTTTCCACATCTTCTTACGTTCAATCATCGTTTGTCTCGTGGAGGGTTGAGAATGTCAAA
WsWf_2          GTTTCCACATCTTCTTACGTTCAATCATCGTTTGTCTCGTGGAGGGTTGAGAATGTCAAA
RsWf_1          GTTTCCACATCTTCTTACGTTCAATCATCGTTTGTCTCGTGGAGGGTTGAGAATGTCAAA
RsWf_2          GTTTCCACATCTTCTTACGTTCAATCATCGTTTGTCTCGTGGAGGGTTGAGAATGTCAAA
                ************************************************************

RsRf_1          GAGCATCAGCAATATCAACGAGAGGAGAAAGCGTCGTCGTCATCCTCGTCGCAATGGATG
RsRf_2          GAGCATCAGCAATATCAACGAGAGGAGAAAGCGTCGTCGTCATCCTCGTCGCAATGGATG
GsRf_2          GAGCATCAGCAATATCAACGAGAGGAGAAAGCGTCGTCGTCATCCTCGTCGCAATGGATG
GsRf_1          GAGCATCAGCAATATCAACGAGAGGAGAAAGCGTCGTCGTCATCCTCGTCGCAATGGATG
WsWf_1          GAGCATCAGCAATATCAACGAGAGGAGAAAGCGTCGTCGTCATCCTCGTCGCAATGGATG
WsWf_2          GAGCATCAGCAATATCAACGAGAGGAGAAAGCGTCGTCGTCATCCTCGTCGCAATGGATG
RsWf_1          GAGCATCAGCAATATCAACGAGAGGAGAAAGCGTCGTCGTCATCCTCGTCGCAATGGATG
RsWf_2          GAGCATCAGCAATATCAACGAGAGGAGAAAGCGTCGTCGTCATCCTCGTCGCAATGGATG
                ************************************************************

RsRf_1          CTCAAACACATGATCTTGAGAGTTCCTTTACTCCATGAAAACACTAAAAACAAGAGGGTG
RsRf_2          CTCAAACACATGATCTTGAGAGTTCCTTTACTCCATGAAAACACTAAAAACAAGAGGGTG
GsRf_2          CTCAAACACATGATCTTGAGAGTTCCTTTACTCCATGAAAACACTAAAAACAAGAGGGTG
GsRf_1          CTCAAACACATGATCTTGAGAGTTCCTTTACTCCATGAAAACACTAAAAACAAGAGGGTG
WsWf_1          CTCAAACACATGATCTTGAGAGTTCCTTTACTCCATGAAAACACTAAAAACAAGAGGGTG
WsWf_2          CTCAAACACATGATCTTGAGAGTTCCTTTACTCCATGAAAACACTAAAAACAAGAGGGTG
RsWf_1          CTCAAACACATGATCTTGAGAGTTCCTTTACTCCATGAAAACACTAAAAACAAGAGGGTG
RsWf_2          CTCAAACACATGATCTTGAGAGTTCCTTTACTCCATGAAAACACTAAAAACAAGAGGGTG
                ************************************************************

RsRf_1          CCGCGGGAAGAGCTCAACCATGTGGTGGCCGAGCGACGCAGAAGAGAGAAGCTTAACGAG
RsRf_2          CCGCGGGAAGAGCTCAACCATGTGGTGGCCGAGCGACGCAGAAGAGAGAAGCTTAACGAG
GsRf_2          CCGCGGGAAGAGCTCAACCATGTGGTGGCCGAGCGACGCAGAAGAGAGAAGCTTAACGAG
GsRf_1          CCGCGGGAAGAGCTCAACCATGTGGTGGCCGAGCGACGCAGAAGAGAGAAGCTTAACGAG
WsWf_1          CCGCGGGAAGAGCTCAACCATGTGGTGGCCGAGCGACGCAGAAGAGAGAAGCTTAACGAG
WsWf_2          CCGCGGGAAGAGCTCAACCATGTGGTGGCCGAGCGACGCAGAAGAGAGAAGCTTAACGAG
RsWf_1          CCGCGGGAAGAGCTCAACCATGTGGTGGCCGAGCGACGCAGAAGAGAGAAGCTTAACGAG
RsWf_2          CCGCGGGAAGAGCTCAACCATGTGGTGGCCGAGCGACGCAGAAGAGAGAAGCTTAACGAG
                ************************************************************

RsRf_1          AGATTCATAACGTTGAGATCATTGGTTCCATTTGTGACCAAGATGGATAAAGTCTCGATC
RsRf_2          AGATTCATAACGTTGAGATCATTGGTTCCATTTGTGACCAAGATGGATAAAGTCTCGATC
GsRf_2          AGATTCATAACGTTGAGATCATTGGTTCCATTTGTGACCAAGATGGATAAAGTCTCGATC
GsRf_1          AGATTCATAACGTTGAGATCATTGGTTCCATTTGTGACCAAGATGGATAAAGTCTCGATC
WsWf_1          AGATTCATAACGTTGAGATCATTGGTTCCATTTGTGACCAAGATGGATAAAGTCTCGATC
WsWf_2          AGATTCATAACGTTGAGATCATTGGTTCCATTTGTGACCAAGATGGATAAAGTCTCGATC
RsWf_1          AGATTCATAACGTTGAGATCATTGGTTCCATTTGTGACCAAGATGGATAAAGTCTCGATC
RsWf_2          AGATTCATAACGTTGAGATCATTGGTTCCATTTGTGACCAAGATGGATAAAGTCTCGATC
                ************************************************************

RsRf_1          CTTGGAGACACCATTGATTACGTAAACCATCTTTGTAAGAGGATCCATGAGCTGGAATCT
RsRf_2          CTTGGAGACACCATTGATTACGTAAACCATCTTTGTAAGAGGATCCATGAGCTGGAATCT
GsRf_2          CTTGGAGACACCATTGATTACGTAAACCATCTTTGTAAGAGGATCCATGAGCTGGAATCT
GsRf_1          CTTGGAGACACCATTGATTACGTAAACCATCTTTGTAAGAGGATCCATGAGCTGGAATCT
WsWf_1          CTTGGAGACACCATTGATTACGTAAACCATCTTTGTAAGAGGATCCATGAGCTGGAATCT
WsWf_2          CTTGGAGACACCATTGATTACGTAAACCATCTTTGTAAGAGGATCCATGAGCTGGAATCT
RsWf_1          CTTGGAGACACCATTGATTACGTAAACCATCTTTGTAAGAGGATCCATGAGCTGGAATCT
RsWf_2          CTTGGAGACACCATTGATTACGTAAACCATCTTTGTAAGAGGATCCATGAGCTGGAATCT
                ************************************************************

RsRf_1          ACTCATCACGAGCCAAACCAAAAGCGGATGCGTATCGGTAAGGGAAGAACGTGGGAAGAG
RsRf_2          ACTCATCACGAGCCAAACCAAAAGCGGATGCGTATCGGTAAGGGAAGAACGTGGGAAGAG
GsRf_2          ACTCATCACGAGCCAAACCAAAAGCGGATGCGTATCGGTAAGGGAAGAACGTGGGAAGAG
GsRf_1          ACTCATCACGAGCCAAACCAAAAGCGGATGCGTATCGGTAAGGGAAGAACGTGGGAAGAG
WsWf_1          ACTCATCACGAGCCAAACCAAAAGCGGATGCGTATCGGTAAGGGAAGAACGTGGGAAGAG
WsWf_2          ACTCATCACGAGCCAAACCAAAAGCGGATGCGTATCGGTAAGGGAAGAACGTGGGAAGAG
RsWf_1          ACTCATCACGAGCCAAACCAAAAGCGGATGCGTATCGGTAAGGGAAGAACGTGGGAAGAG
RsWf_2          ACTCATCACGAGCCAAACCAAAAGCGGATGCGTATCGGTAAGGGAAGAACGTGGGAAGAG
                ************************************************************

RsRf_1          GTGGAGGTTTCCATTATAGAGAGCGATGTTTTGTTAGAGATGAGATGCGAGTACCGAGAT
RsRf_2          GTGGAGGTTTCCATTATAGAGAGCGATGTTTTGTTAGAGATGAGATGCGAGTACCGAGAT
GsRf_2          GTGGAGGTTTCCATTATAGAGAGCGATGTTTTGTTAGAGATGAGATGCGAGTACCGAGAT
GsRf_1          GTGGAGGTTTCCATTATAGAGAGCGATGTTTTGTTAGAGATGAGATGCGAGTACCGAGAT
WsWf_1          GTGGAGGTTTCCATTATAGAGAGCGATGTTTTGTTAGAGATGAGATGCGAGTACCGAGAT
WsWf_2          GTGGAGGTTTCCATTATAGAGAGCGATGTTTTGTTAGAGATGAGATGCGAGTACCGAGAT
RsWf_1          GTGGAGGTTTCCATTATAGAGAGCGATGTTTTGTTAGAGATGAGATGCGAGTACCGAGAT
RsWf_2          GTGGAGGTTTCCATTATAGAGAGCGATGTTTTGTTAGAGATGAGATGCGAGTACCGAGAT
                ************************************************************

RsRf_1          GGTTTATTGCTCAACATTCTTCAGGTACTTAAGGAGCTGGGTATAGAGACCACTGCAGTT
RsRf_2          GGTTTATTGCTCAACATTCTTCAGGTACTTAAGGAGCTGGGTATAGAGACCACTGCAGTT
GsRf_2          GGTTTATTGCTCAACATTCTTCAGGTACTTAAGGAGCTGGGTATAGAGACCACTGCAGTT
GsRf_1          GGTTTATTGCTCAACATTCTTCAGGTACTTAAGGAGCTGGGTATAGAGACCACTGCAGTT
WsWf_1          GGTTTATTGCTCAACATTCTTCAGGTACTTAAGGAGCTGGGTATAGAGACCACTGCAGTT
WsWf_2          GGTTTATTGCTCAACATTCTTCAGGTACTTAAGGAGCTGGGTATAGAGACCACTGCAGTT
RsWf_1          GGTTTATTGCTCAACATTCTTCAGGTACTTAAGGAGCTGGGTATAGAGACCACTGCAGTT
RsWf_2          GGTTTATTGCTCAACATTCTTCAGGTACTTAAGGAGCTGGGTATAGAGACCACTGCAGTT
                ************************************************************

RsRf_1          CACACCGCCGTGAACGACCATGATTTTGAGGCAGAGATAAGGGCGAAAGTGAGAGGGAAG
RsRf_2          CACACCGCCGTGAACGACCATGATTTTGAGGCAGAGATAAGGGCGAAAGTGAGAGGGAAG
GsRf_2          CACACCGCCGTGAACGACCATGATTTTGAGGCAGAGATAAGGGCGAAAGTGAGAGGGAAG
GsRf_1          CACACCGCCGTGAACGACCATGATTTTGAGGCAGAGATAAGGGCGAAAGTGAGAGGGAAG
WsWf_1          CACACCGCCGTGAACGACCATGATTTTGAGGCAGAGATAAGGGCGAAAGTGAGAGGGAAG
WsWf_2          CACACCGCCGTGAACGACCATGATTTTGAGGCAGAGATAAGGGCGAAAGTGAGAGGGAAG
RsWf_1          CACACCGCCGTGAACGACCATGATTTTGAGGCAGAGATAAGGGCGAAAGTGAGAGGGAAG
RsWf_2          CACACCGCCGTGAACGACCATGATTTTGAGGCAGAGATAAGGGCGAAAGTGAGAGGGAAG
                ************************************************************

RsRf_1          AAACCAACCATTGCTGAGGTTAAAATAGCCATCCATCAAATCATATCTCAAAATAAACTC
RsRf_2          AAACCAACCATTGCTGAGGTTAAAATAGCCATCCATCAAATCATATCTCAAAATAAACTC
```

```
GsRf_2          AAACCAACCATTGCTGAGGTTAAAATAGCCATCCATCAAATCATATCTCAAAATAAACTC
GsRf_1          AAACCAACCATTGCTGAGGTTAAAATAGCCATCCATCAAATCATATCTCAAAATAAACTC
WsWf_1          AAACCAACCATTGCTGAGGTTAAAATAGCCATCCATCAAATCATATCTCAAAATAAACTC
WsWf_2          AAACCAACCATTGCTGAGGTTAAAATAGCCATCCATCAAATCATATCTCAAAATAAACTC
RsWf_1          AAACCAACCATTGCTGAGGTTAAAATAGCCATCCATCAAATCATATCTCAAAATAAACTC
RsWf_2          AAACCAACCATTGCTGAGGTTAAAATAGCCATCCATCAAATCATATCTCAAAATAAACTC
                ************************************************************

RsRf_1          TAG  1560
RsRf_2          TAG  1560
GsRf_2          TAG  1560
GsRf_1          TAG  1560
WsWf_1          TAG  1560
WsWf_2          TAG  1560
RsWf_1          TAG  1563
RsWf_2          TAG  1563
                ***
```

```
>RsWf_1
MDESSIIPVWKVIGAEGKEIQGLLKAVVQSVGWTYSLFWQLCPQRRKLVWSSGFYNGAIKTRKTTQPAEITAEEAALERSQQLMELYQTLFAGESSMEARASTALSPEDLTDTEWFYVLCLTYS
FEPPSGMPGKAYARRKQVWMSGVNEVDSKIFSRAISAKSAKIQTVVCIPVLDGVLEIGTTNKVKENEEFVEHIKSFFQNHPKSNTKPALFEHSINEDHEEDEEEVEEMTMSEEIRLGSPDDDDV
SNQNLLSDFHIEAPNSLDTQMDMMNLMEEGGNYSQTVSTLLMSQLPNLLSDSVSTSSYVQSSFVSWRVENVKEHQQYQREEKASSSSSSQWMLKHMILRVPLLHENTKNKRVPREELNHVVAER
RRREKLNERFITLRSLVPFVTKMDKVSILGDTIDYVNHLCKRIHELESTHHEPNQKRMRIGKGRTWEEVEVSIIESDVLLEMRCEYRDGLLLNILQVLKELGIETTAVHTAVNDHDFEAEIRAK
VRGKKPTIAEVKIAIHQIISQNKL

>RsWf_2
MDESSIIPVWKVIGAEGKEIQGLLKAVVQSVGWTYSLFWQLCPQRRKLVWSSGFYNGAIKTRKTTQPAEITAEEAALERSQQLMELYQTLFAGESSMEARASTALSPEDLTDTEWFYVLCLTYS
FEPPSGMPGKAYARRKQVWMSGVNEVDSKIFSRAISAKSAKIQTVVCIPVLDGVLEIGTTNKVKENEEFVEHIKSFFQNHPKSNTKPALFEHSINEDHEEDEEEVEEMTMSEEIRLGSPDDDDV
SNQNLLSDFHIEAPNSLDTQMDMMNLMEEGGNYSQTVSTLLMSQLPNLLSDSVSTSSYVQSSFVSWRVENVKEHQQYQREEKASSSSSSQWMLKHMILRVPLLHENTKNKRVPREELNHVVAER
RRREKLNERFITLRSLVPFVTKMDKVSILGDTIDYVNHLCKRIHELESTHHEPNQKRMRIGKGRTWEEVEVSIIESDVLLEMRCEYRDGLLLNILQVLKELGIETTAVHTAVNDHDFEAEIRAK
VRGKKPTIAEVKIAIHQIISQNKL

>GsRf_1
MDESSIIPVWKVIGAEEKEIQGLLKAVVQSVGWTYSLFWQLCPQRRKLVWSSGFYNGAIKTRKTTQPAEITAEEAALERSQQLMELYQTLFAGESSMEARACTALSPEDLTDTEWFYVLCLTYS
FEPPSGMPGKAYARRKQVWMSGVNEVDSKIFSRAISAKSAKIQTVVCIPVLDGVLEIGTTNKVKENEEFVEHMKSFFQNHPKSNTKPALFEHSINEEHEEDEEVEEMTMSEEIRLGSPDDDDVS
NQNLLSDFHIEAPSSLDTQMDMMNLMEEGGNYSQTVSTLLMSQLPNLLSDSVSTSSYVQSSFVSWRVENVKEHQQYQREEKASSSSSSQWMLKHMILRVPLLHENTKNKRVPREELNHVVAERR
RREKLNERFITLRSLVPFVTKMDKVSILGDTIDYVNHLCKRIHELESTHHEPNQKRMRIGKGRTWEEVEVSIIESDVLLEMRCEYRDGLLLNILQVLKELGIETTAVHTAVNDHDFEAEIRAKV
RGKKPTIAEVKIAIHQIISQNKL

>GsRf_2
MDESSIIPVWKVIGAEEKEIQGLLKAVVQSVGWTYSLFWQLCPQRRKLVWSSGFYNGAIKTRKTTQPAEITAEEAALERSQQLMELYQTLFAGESSMEARACTALSPEDLTDTEWFYVLCLTYS
FEPPSGMPGKAYARRKQVWMSGVNEVDSKIFSRAISAKSAKIQTVVCIPVLDGVLEIGTTNKVKENEEFVEHMKSFFQNHPKSNTKPALFEHSINEEHEEDEEVEEMTMSEEIRLGSPDDDDVS
NQNLLSDFHIEAPSSLDTQMDMMNLMEEGGNYSQTVSTLLMSQLPNLLSDSVSTSSYVQSSFVSWRVENVKEHQQYQREEKASSSSSSQWMLKHMILRVPLLHENTKNKRVPREELNHVVAERR
RREKLNERFITLRSLVPFVTKMDKVSILGDTIDYVNHLCKRIHELESTHHEPNQKRMRIGKGRTWEEVEVSIIESDVLLEMRCEYRDGLLLNILQVLKELGIETTAVHTAVNDHDFEAEIRAKV
RGKKPTIAEVKIAIHQIISQNKL

>RsRf_1
MDESSIIPVWKVIGAEEKEIQGLLKAVVQSVGWTYSLFWQLCPQRRKLVWSSGFYNGAIKTRKTTQPAEITAEEAALERSQQLMELYQTLFAGESSMEARACTALSPEDLTDTEWFYVLCLTYS
FEPPSGMPGKAYARRKQVWMSGVNEVDSKIFSRAISAKSAKIQTVVCIPVLDGVLEIGTTNKVKENEEFVEHMKSFFQNHPKSNTKPALFEHSINEEHEEDEEVEEMTMSEEIRLGSPDDDDVS
NQNLLSDFHIEAPSSLDTQMDMMNLMEEGGNYSQTVSTLLMSQLPNLLSDSVSTSSYVQSSFVSWRVENVKEHQQYQREEKASSSSSSQWMLKHMILRVPLLHENTKNKRVPREELNHVVAERR
RREKLNERFITLRSLVPFVTKMDKVSILGDTIDYVNHLCKRIHELESTHHEPNQKRMRIGKGRTWEEVEVSIIESDVLLEMRCEYRDGLLLNILQVLKELGIETTAVHTAVNDHDFEAEIRAKV
RGKKPTIAEVKIAIHQIISQNKL

>RsRf_2
MDESSIIPVWKVIGAEEKEIQGLLKAVVQSVGWTYSLFWQLCPQRRKLVWSSGFYNGAIKTRKTTQPAEITAEEAALERSQQLMELYQTLFAGESSMEARACTALSPEDLTDTEWFYVLCLTYS
FEPPSGMPGKAYARRKQVWMSGVNEVDSKIFSRAISAKSAKIQTVVCIPVLDGVLEIGTTNKVKENEEFVEHMKSFFQNHPKSNTKPALFEHSINEEHEEDEEVEEMTMSEEIRLGSPDDDDVS
NQNLLSDFHIEAPSSLDTQMDMMNLMEEGGNYSQTVSTLLMSQLPNLLSDSVSTSSYVQSSFVSWRVENVKEHQQYQREEKASSSSSSQWMLKHMILRVPLLHENTKNKRVPREELNHVVAERR
RREKLNERFITLRSLVPFVTKMDKVSILGDTIDYVNHLCKRIHELESTHHEPNQKRMRIGKGRTWEEVEVSIIESDVLLEMRCEYRDGLLLNILQVLKELGIETTAVHTAVNDHDFEAEIRAKV
RGKKPTIAEVKIAIHQIISQNKL

>WsWf_1
MDESSIIPVWKVIGAEEKEIQGLLKAVVQSVGWTYSLFWQLCPQRRKLVWSSGFYNGAIKTRKTTQPAEITAEEAALERSQQLMELYQTLFAGESSMEARACTALSPEDLTDTEWFYVLCLTYS
FEPPSGMPGKAYARRKQVWMSGVNEVDSKIFSRAISAKSAKIQTVVCIPVLDGVLEIGTTNKVKENEEFVEHIKSFFQNHPKSNTKPALFEHSINEEHEEDEEVEEMTMSEEIRLGSPDDDDVS
NQNLLSDFHIEAPNSLDTQMDMMNLMEEGGSYSQTVSTLLMSQLPNLLSDSVSTSSYVQSSFVSWRVENVKEHQQYQREEKASSSSSSQWMLKHMILRVPLLHENTKNKRVPREELNHVVAERR
RREKLNERFITLRSLVPFVTKMDKVSILGDTIDYVNHLCKRIHELESTHHEPNQKRMRIGKGRTWEEVEVSIIESDVLLEMRCEYRDGLLLNILQVLKELGIETTAVHTAVNDHDFEAEIRAKV
RGKKPTIAEVKIAIHQIISQNKL

>WsWf_2
MDESSIIPVWKVIGAEEKEIQGLLKAVVQSVGWTYSLFWQLCPQRRKLVWSSGFYNGAIKTRKTTQPAEITAEEAALERSQQLMELYQTLFAGESSMEARACTALSPEDLTDTEWFYVLCLTYS
FEPPSGMPGKAYARRKQVWMSGVNEVDSKIFSRAISAKSAKIQTVVCIPVLDGVLEIGTTNKVKENEEFVEHIKSFFQNHPKSNTKPALFEHSINEEHEEDEEVEEMTMSEEIRLGSPDDDDVS
NQNLLSDFHIEAPNSLDTQMDMMNLMEEGGSYSQTVSTLLMSQLPNLLSDSVSTSSYVQSSFVSWRVENVKEHQQYQREEKASSSSSSQWMLKHMILRVPLLHENTKNKRVPREELNHVVAERR
RREKLNERFITLRSLVPFVTKMDKVSILGDTIDYVNHLCKRIHELESTHHEPNQKRMRIGKGRTWEEVEVSIIESDVLLEMRCEYRDGLLLNILQVLKELGIETTAVHTAVNDHDFEAEIRAKV
RGKKPTIAEVKIAIHQIISQNKL
```

```
GsRf_2    MDESSIIPVWKVIGAEEKEIQGLLKAVVQSVGWTYSLFWQLCPQRRKLVWSSGFYNGAIKTRKTTQPAEITAEEAALERSQQLMELYQTLFAGESSMEAR   100
RsRf_1    MDESSIIPVWKVIGAEEKEIQGLLKAVVQSVGWTYSLFWQLCPQRRKLVWSSGFYNGAIKTRKTTQPAEITAEEAALERSQQLMELYQTLFAGESSMEAR
RsRf_2    MDESSIIPVWKVIGAEEKEIQGLLKAVVQSVGWTYSLFWQLCPQRRKLVWSSGFYNGAIKTRKTTQPAEITAEEAALERSQQLMELYQTLFAGESSMEAR
GsRf_1    MDESSIIPVWKVIGAEEKEIQGLLKAVVQSVGWTYSLFWQLCPQRRKLVWSSGFYNGAIKTRKTTQPAEITAEEAALERSQQLMELYQTLFAGESSMEAR
WsWf_1    MDESSIIPVWKVIGAEEKEIQGLLKAVVQSVGWTYSLFWQLCPQRRKLVWSSGFYNGAIKTRKTTQPAEITAEEAALERSQQLMELYQTLFAGESSMEAR
WsWf_2    MDESSIIPVWKVIGAEEKEIQGLLKAVVQSVGWTYSLFWQLCPQRRKLVWSSGFYNGAIKTRKTTQPAEITAEEAALERSQQLMELYQTLFAGESSMEAR
RsWf_1    MDESSIIPVWKVIGAEGKEIQGLLKAVVQSVGWTYSLFWQLCPQRRKLVWSSGFYNGAIKTRKTTQPAEITAEEAALERSQQLMELYQTLFAGESSMEAR
RsWf_2    MDESSIIPVWKVIGAEGKEIQGLLKAVVQSVGWTYSLFWQLCPQRRKLVWSSGFYNGAIKTRKTTQPAEITAEEAALERSQQLMELYQTLFAGESSMEAR
          **************** ***************************************************************************************

                        MIR

GsRf_2    ACTALSPEDLTDTEWFYVLCLTYSFEPPSGMPGKAYARRKQVWMSGVNEVDSKIFSRAISAKSAKIQTVVCIPVLDGVLEIGTTNKVKENEEFVEHMKSF   200
RsRf_1    ACTALSPEDLTDTEWFYVLCLTYSFEPPSGMPGKAYARRKQVWMSGVNEVDSKIFSRAISAKSAKIQTVVCIPVLDGVLEIGTTNKVKENEEFVEHMKSF
RsRf_2    ACTALSPEDLTDTEWFYVLCLTYSFEPPSGMPGKAYARRKQVWMSGVNEVDSKIFSRAISAKSAKIQTVVCIPVLDGVLEIGTTNKVKENEEFVEHMKSF
GsRf_1    ACTALSPEDLTDTEWFYVLCLTYSFEPPSGMPGKAYARRKQVWMSGVNEVDSKIFSRAISAKSAKIQTVVCIPVLDGVLEIGTTNKVKENEEFVEHMKSF
WsWf_1    ACTALSPEDLTDTEWFYVLCLTYSFEPPSGMPGKAYARRKQVWMSGVNEVDSKIFSRAISAKSAKIQTVVCIPVLDGVLEIGTTNKVKENEEFVEHIKSF
WsWf_2    ACTALSPEDLTDTEWFYVLCLTYSFEPPSGMPGKAYARRKQVWMSGVNEVDSKIFSRAISAKSAKIQTVVCIPVLDGVLEIGTTNKVKENEEFVEHIKSF
RsWf_1    ASTALSPEDLTDTEWFYVLCLTYSFEPPSGMPGKAYARRKQVWMSGVNEVDSKIFSRAISAKSAKIQTVVCIPVLDGVLEIGTTNKVKENEEFVEHIKSF
RsWf_2    ASTALSPEDLTDTEWFYVLCLTYSFEPPSGMPGKAYARRKQVWMSGVNEVDSKIFSRAISAKSAKIQTVVCIPVLDGVLEIGTTNKVKENEEFVEHIKSF
          *.*********************************************************************************************:***

                                            WD/AD
GsRf_2    FQNHPKSNTKPALFEHSINEEHEEDEE-VEEMTMSEEIRLGSPDDDDVSNQNLLSDFHIEAPSSLDTQMDMMNLMEEGGNYSQTVSTLLMSQLPNLLSDS   300
RsRf_1    FQNHPKSNTKPALFEHSINEEHEEDEE-VEEMTMSEEIRLGSPDDDDVSNQNLLSDFHIEAPSSLDTQMDMMNLMEEGGNYSQTVSTLLMSQLPNLLSDS
RsRf_2    FQNHPKSNTKPALFEHSINEEHEEDEE-VEEMTMSEEIRLGSPDDDDVSNQNLLSDFHIEAPSSLDTQMDMMNLMEEGGNYSQTVSTLLMSQLPNLLSDS
GsRf_1    FQNHPKSNTKPALFEHSINEEHEEDEE-VEEMTMSEEIRLGSPDDDDVSNQNLLSDFHIEAPSSLDTQMDMMNLMEEGGNYSQTVSTLLMSQLPNLLSDS
WsWf_1    FQNHPKSNTKPALFEHSINEEHEEDEE-VEEMTMSEEIRLGSPDDDDVSNQNLLSDFHIEAPNSLDTQMDMMNLMEEGGSYSQTVSTLLMSQLPNLLSDS
WsWf_2    FQNHPKSNTKPALFEHSINEEHEEDEE-VEEMTMSEEIRLGSPDDDDVSNQNLLSDFHIEAPNSLDTQMDMMNLMEEGGSYSQTVSTLLMSQLPNLLSDS
RsWf_1    FQNHPKSNTKPALFEHSINEDHEEDEEEVEEMTMSEEIRLGSPDDDDVSNQNLLSDFHIEAPNSLDTQMDMMNLMEEGGNYSQTVSTLLMSQLPNLLSDS
RsWf_2    FQNHPKSNTKPALFEHSINEDHEEDEEEVEEMTMSEEIRLGSPDDDDVSNQNLLSDFHIEAPNSLDTQMDMMNLMEEGGNYSQTVSTLLMSQLPNLLSDS
          ********************:****** **********************************.****************.********************

                                                                  basic       Helix 1       Loop
                                                                ---------------------------------------
GsRf_2    VSTSSYVQSSFVSWRVENVKEHQQYQREEKASSSSSSQWMLKHMILRVPLLHENTKNKRVPREELNHVVAERRRREKLNERFITLRSLVPFVTKMDKVSI   400
RsRf_1    VSTSSYVQSSFVSWRVENVKEHQQYQREEKASSSSSSQWMLKHMILRVPLLHENTKNKRVPREELNHVVAERRRREKLNERFITLRSLVPFVTKMDKVSI
RsRf_2    VSTSSYVQSSFVSWRVENVKEHQQYQREEKASSSSSSQWMLKHMILRVPLLHENTKNKRVPREELNHVVAERRRREKLNERFITLRSLVPFVTKMDKVSI
GsRf_1    VSTSSYVQSSFVSWRVENVKEHQQYQREEKASSSSSSQWMLKHMILRVPLLHENTKNKRVPREELNHVVAERRRREKLNERFITLRSLVPFVTKMDKVSI
WsWf_1    VSTSSYVQSSFVSWRVENVKEHQQYQREEKASSSSSSQWMLKHMILRVPLLHENTKNKRVPREELNHVVAERRRREKLNERFITLRSLVPFVTKMDKVSI
WsWf_2    VSTSSYVQSSFVSWRVENVKEHQQYQREEKASSSSSSQWMLKHMILRVPLLHENTKNKRVPREELNHVVAERRRREKLNERFITLRSLVPFVTKMDKVSI
RsWf_1    VSTSSYVQSSFVSWRVENVKEHQQYQREEKASSSSSSQWMLKHMILRVPLLHENTKNKRVPREELNHVVAERRRREKLNERFITLRSLVPFVTKMDKVSI
RsWf_2    VSTSSYVQSSFVSWRVENVKEHQQYQREEKASSSSSSQWMLKHMILRVPLLHENTKNKRVPREELNHVVAERRRREKLNERFITLRSLVPFVTKMDKVSI
          ****************************************************************************************************
                                                                     bHLH region

             Helix 2
          ------------------
GsRf_2    LGDTIDYVNHLCKRIHELESTHHEPNQKRMRIGKGRTWEEVEVSIIESDVLLEMRCEYRDGLLLNILQVLKELGIETTAVHTAVNDHDFEAEIRAKVRGK   500
RsRf_1    LGDTIDYVNHLCKRIHELESTHHEPNQKRMRIGKGRTWEEVEVSIIESDVLLEMRCEYRDGLLLNILQVLKELGIETTAVHTAVNDHDFEAEIRAKVRGK
RsRf_2    LGDTIDYVNHLCKRIHELESTHHEPNQKRMRIGKGRTWEEVEVSIIESDVLLEMRCEYRDGLLLNILQVLKELGIETTAVHTAVNDHDFEAEIRAKVRGK
GsRf_1    LGDTIDYVNHLCKRIHELESTHHEPNQKRMRIGKGRTWEEVEVSIIESDVLLEMRCEYRDGLLLNILQVLKELGIETTAVHTAVNDHDFEAEIRAKVRGK
WsWf_1    LGDTIDYVNHLCKRIHELESTHHEPNQKRMRIGKGRTWEEVEVSIIESDVLLEMRCEYRDGLLLNILQVLKELGIETTAVHTAVNDHDFEAEIRAKVRGK
WsWf_2    LGDTIDYVNHLCKRIHELESTHHEPNQKRMRIGKGRTWEEVEVSIIESDVLLEMRCEYRDGLLLNILQVLKELGIETTAVHTAVNDHDFEAEIRAKVRGK
RsWf_1    LGDTIDYVNHLCKRIHELESTHHEPNQKRMRIGKGRTWEEVEVSIIESDVLLEMRCEYRDGLLLNILQVLKELGIETTAVHTAVNDHDFEAEIRAKVRGK
RsWf_2    LGDTIDYVNHLCKRIHELESTHHEPNQKRMRIGKGRTWEEVEVSIIESDVLLEMRCEYRDGLLLNILQVLKELGIETTAVHTAVNDHDFEAEIRAKVRGK
          ****************************************************************************************************
                                          ACT-like domain: dimerization

GsRf_2    KPTIAEVKIAIHQIISQNKL  519
RsRf_1    KPTIAEVKIAIHQIISQNKL  519
RsRf_2    KPTIAEVKIAIHQIISQNKL  519
GsRf_1    KPTIAEVKIAIHQIISQNKL  519
WsWf_1    KPTIAEVKIAIHQIISQNKL  519
WsWf_2    KPTIAEVKIAIHQIISQNKL  519
RsWf_1    KPTIAEVKIAIHQIISQNKL  520
RsWf_2    KPTIAEVKIAIHQIISQNKL  520
          ********************
```

```
GsRf_2     MDESSIIPVWKVIGAEEKEIQGLLKAVVQSVGWTYSLFWQLCPQRRKLVWSSGFYNGAIKTRKTTQPAEITAEEAALERSQQLMELYQTLFAGESSMEAR   100
RsRf_1     MDESSIIPVWKVIGAEEKEIQGLLKAVVQSVGWTYSLFWQLCPQRRKLVWSSGFYNGAIKTRKTTQPAEITAEEAALERSQQLMELYQTLFAGESSMEAR
RsRf_2     MDESSIIPVWKVIGAEEKEIQGLLKAVVQSVGWTYSLFWQLCPQRRKLVWSSGFYNGAIKTRKTTQPAEITAEEAALERSQQLMELYQTLFAGESSMEAR
GsRf_1     MDESSIIPVWKVIGAEEKEIQGLLKAVVQSVGWTYSLFWQLCPQRRKLVWSSGFYNGAIKTRKTTQPAEITAEEAALERSQQLMELYQTLFAGESSMEAR
WsWf_1     MDESSIIPVWKVIGAEEKEIQGLLKAVVQSVGWTYSLFWQLCPQRRKLVWSSGFYNGAIKTRKTTQPAEITAEEAALERSQQLMELYQTLFAGESSMEAR
WsWf_2     MDESSIIPVWKVIGAEEKEIQGLLKAVVQSVGWTYSLFWQLCPQRRKLVWSSGFYNGAIKTRKTTQPAEITAEEAALERSQQLMELYQTLFAGESSMEAR
RsWf_1     MDESSIIPVWKVIGAEGKEIQGLLKAVVQSVGWTYSLFWQLCPQRRKLVWSSGFYNGAIKTRKTTQPAEITAEEAALERSQQLMELYQTLFAGESSMEAR
RsWf_2     MDESSIIPVWKVIGAEGKEIQGLLKAVVQSVGWTYSLFWQLCPQRRKLVWSSGFYNGAIKTRKTTQPAEITAEEAALERSQQLMELYQTLFAGESSMEAR
           **************** ***************************************************************************************

GsRf_2     ACTALSPEDLTDTEWFYVLCLTYSFEPPSGMPGKAYARRKQVWMSGVNEVDSKIFSRAISAKSAKIQTVVCIPVLDGVLEIGTTNKVKENEEFVEHMKSF   200
RsRf_1     ACTALSPEDLTDTEWFYVLCLTYSFEPPSGMPGKAYARRKQVWMSGVNEVDSKIFSRAISAKSAKIQTVVCIPVLDGVLEIGTTNKVKENEEFVEHMKSF
RsRf_2     ACTALSPEDLTDTEWFYVLCLTYSFEPPSGMPGKAYARRKQVWMSGVNEVDSKIFSRAISAKSAKIQTVVCIPVLDGVLEIGTTNKVKENEEFVEHMKSF
GsRf_1     ACTALSPEDLTDTEWFYVLCLTYSFEPPSGMPGKAYARRKQVWMSGVNEVDSKIFSRAISAKSAKIQTVVCIPVLDGVLEIGTTNKVKENEEFVEHMKSF
WsWf_1     ACTALSPEDLTDTEWFYVLCLTYSFEPPSGMPGKAYARRKQVWMSGVNEVDSKIFSRAISAKSAKIQTVVCIPVLDGVLEIGTTNKVKENEEFVEHIKSF
WsWf_2     ACTALSPEDLTDTEWFYVLCLTYSFEPPSGMPGKAYARRKQVWMSGVNEVDSKIFSRAISAKSAKIQTVVCIPVLDGVLEIGTTNKVKENEEFVEHIKSF
RsWf_1     ASTALSPEDLTDTEWFYVLCLTYSFEPPSGMPGKAYARRKQVWMSGVNEVDSKIFSRAISAKSAKIQTVVCIPVLDGVLEIGTTNKVKENEEFVEHIKSF
RsWf_2     ASTALSPEDLTDTEWFYVLCLTYSFEPPSGMPGKAYARRKQVWMSGVNEVDSKIFSRAISAKSAKIQTVVCIPVLDGVLEIGTTNKVKENEEFVEHIKSF
           *.**********************************************************************************************:***

GsRf_2     FQNHPKSNTKPALFEHSINEEHEEDEE-VEEMTMSEEIRLGSPDDDDVSNQNLLSDFHIEAPSSLDTQMDMMNLMEEGGNYSQTVSTLLMSQLPNLLSDS   300
RsRf_1     FQNHPKSNTKPALFEHSINEEHEEDEE-VEEMTMSEEIRLGSPDDDDVSNQNLLSDFHIEAPSSLDTQMDMMNLMEEGGNYSQTVSTLLMSQLPNLLSDS
RsRf_2     FQNHPKSNTKPALFEHSINEEHEEDEE-VEEMTMSEEIRLGSPDDDDVSNQNLLSDFHIEAPSSLDTQMDMMNLMEEGGNYSQTVSTLLMSQLPNLLSDS
GsRf_1     FQNHPKSNTKPALFEHSINEEHEEDEE-VEEMTMSEEIRLGSPDDDDVSNQNLLSDFHIEAPSSLDTQMDMMNLMEEGGNYSQTVSTLLMSQLPNLLSDS
WsWf_1     FQNHPKSNTKPALFEHSINEEHEEDEE-VEEMTMSEEIRLGSPDDDDVSNQNLLSDFHIEAPNSLDTQMDMMNLMEEGGSYSQTVSTLLMSQLPNLLSDS
WsWf_2     FQNHPKSNTKPALFEHSINEEHEEDEE-VEEMTMSEEIRLGSPDDDDVSNQNLLSDFHIEAPNSLDTQMDMMNLMEEGGSYSQTVSTLLMSQLPNLLSDS
RsWf_1     FQNHPKSNTKPALFEHSINEDHEEDEEEVEEMTMSEEIRLGSPDDDDVSNQNLLSDFHIEAPNSLDTQMDMMNLMEEGGNYSQTVSTLLMSQLPNLLSDS
RsWf_2     FQNHPKSNTKPALFEHSINEDHEEDEEEVEEMTMSEEIRLGSPDDDDVSNQNLLSDFHIEAPNSLDTQMDMMNLMEEGGNYSQTVSTLLMSQLPNLLSDS
           ********************:****** **********************************.****************.********************

                                                                         --------------------------------------
GsRf_2     VSTSSYVQSSFVSWRVENVKEHQQYQREEKASSSSSSQWMLKHMILRVPLLHENTKNKRVPREELNHVVAERRRREKLNERFITLRSLVPFVTKMDKVSI   400
RsRf_1     VSTSSYVQSSFVSWRVENVKEHQQYQREEKASSSSSSQWMLKHMILRVPLLHENTKNKRVPREELNHVVAERRRREKLNERFITLRSLVPFVTKMDKVSI
RsRf_2     VSTSSYVQSSFVSWRVENVKEHQQYQREEKASSSSSSQWMLKHMILRVPLLHENTKNKRVPREELNHVVAERRRREKLNERFITLRSLVPFVTKMDKVSI
GsRf_1     VSTSSYVQSSFVSWRVENVKEHQQYQREEKASSSSSSQWMLKHMILRVPLLHENTKNKRVPREELNHVVAERRRREKLNERFITLRSLVPFVTKMDKVSI
WsWf_1     VSTSSYVQSSFVSWRVENVKEHQQYQREEKASSSSSSQWMLKHMILRVPLLHENTKNKRVPREELNHVVAERRRREKLNERFITLRSLVPFVTKMDKVSI
WsWf_2     VSTSSYVQSSFVSWRVENVKEHQQYQREEKASSSSSSQWMLKHMILRVPLLHENTKNKRVPREELNHVVAERRRREKLNERFITLRSLVPFVTKMDKVSI
RsWf_1     VSTSSYVQSSFVSWRVENVKEHQQYQREEKASSSSSSQWMLKHMILRVPLLHENTKNKRVPREELNHVVAERRRREKLNERFITLRSLVPFVTKMDKVSI
RsWf_2     VSTSSYVQSSFVSWRVENVKEHQQYQREEKASSSSSSQWMLKHMILRVPLLHENTKNKRVPREELNHVVAERRRREKLNERFITLRSLVPFVTKMDKVSI
           ****************************************************************************************************

           ------------------
GsRf_2     LGDTIDYVNHLCKRIHELESTHHEPNQKRMRIGKGRTWEEVEVSIIESDVLLEMRCEYRDGLLLNILQVLKELGIETTAVHTAVNDHDFEAEIRAKVRGK   500
RsRf_1     LGDTIDYVNHLCKRIHELESTHHEPNQKRMRIGKGRTWEEVEVSIIESDVLLEMRCEYRDGLLLNILQVLKELGIETTAVHTAVNDHDFEAEIRAKVRGK
RsRf_2     LGDTIDYVNHLCKRIHELESTHHEPNQKRMRIGKGRTWEEVEVSIIESDVLLEMRCEYRDGLLLNILQVLKELGIETTAVHTAVNDHDFEAEIRAKVRGK
GsRf_1     LGDTIDYVNHLCKRIHELESTHHEPNQKRMRIGKGRTWEEVEVSIIESDVLLEMRCEYRDGLLLNILQVLKELGIETTAVHTAVNDHDFEAEIRAKVRGK
WsWf_1     LGDTIDYVNHLCKRIHELESTHHEPNQKRMRIGKGRTWEEVEVSIIESDVLLEMRCEYRDGLLLNILQVLKELGIETTAVHTAVNDHDFEAEIRAKVRGK
WsWf_2     LGDTIDYVNHLCKRIHELESTHHEPNQKRMRIGKGRTWEEVEVSIIESDVLLEMRCEYRDGLLLNILQVLKELGIETTAVHTAVNDHDFEAEIRAKVRGK
RsWf_1     LGDTIDYVNHLCKRIHELESTHHEPNQKRMRIGKGRTWEEVEVSIIESDVLLEMRCEYRDGLLLNILQVLKELGIETTAVHTAVNDHDFEAEIRAKVRGK
RsWf_2     LGDTIDYVNHLCKRIHELESTHHEPNQKRMRIGKGRTWEEVEVSIIESDVLLEMRCEYRDGLLLNILQVLKELGIETTAVHTAVNDHDFEAEIRAKVRGK
           ****************************************************************************************************

GsRf_2     KPTIAEVKIAIHQIISQNKL  519
RsRf_1     KPTIAEVKIAIHQIISQNKL  519
RsRf_2     KPTIAEVKIAIHQIISQNKL  519
GsRf_1     KPTIAEVKIAIHQIISQNKL  519
WsWf_1     KPTIAEVKIAIHQIISQNKL  519
WsWf_2     KPTIAEVKIAIHQIISQNKL  519
RsWf_1     KPTIAEVKIAIHQIISQNKL  520
RsWf_2     KPTIAEVKIAIHQIISQNKL  520
           ********************
```

```
RsRf_1          MDESSIIPVWKVIGAEEKEIQGLLKAVVQSVGWTYSLFWQLCPQRRKLVWSSGFYNGAIK
RsRf_2          MDESSIIPVWKVIGAEEKEIQGLLKAVVQSVGWTYSLFWQLCPQRRKLVWSSGFYNGAIK
GsRf_2          MDESSIIPVWKVIGAEEKEIQGLLKAVVQSVGWTYSLFWQLCPQRRKLVWSSGFYNGAIK
GsRf_1          MDESSIIPVWKVIGAEEKEIQGLLKAVVQSVGWTYSLFWQLCPQRRKLVWSSGFYNGAIK
WsWf_1          MDESSIIPVWKVIGAEEKEIQGLLKAVVQSVGWTYSLFWQLCPQRRKLVWSSGFYNGAIK
WsWf_2          MDESSIIPVWKVIGAEEKEIQGLLKAVVQSVGWTYSLFWQLCPQRRKLVWSSGFYNGAIK
RsWf_1          MDESSIIPVWKVIGAEGKEIQGLLKAVVQSVGWTYSLFWQLCPQRRKLVWSSGFYNGAIK
RsWf_2          MDESSIIPVWKVIGAEGKEIQGLLKAVVQSVGWTYSLFWQLCPQRRKLVWSSGFYNGAIK
AtTT8           MDESSIIPAEKVAGAEKKELQGLLKTAVQSVDWTYSVFWQFCPQQRVLVWGNGYYNGAIK
                ********. ** *** **:*****:.****.****:***:***:* ***..*:******

RsRf_1          TRKTTQPAEITAEEAALERSQQLMELYQTLFAGESSMEARACTALSPEDLTDTEWFYVLC
RsRf_2          TRKTTQPAEITAEEAALERSQQLMELYQTLFAGESSMEARACTALSPEDLTDTEWFYVLC
GsRf_2          TRKTTQPAEITAEEAALERSQQLMELYQTLFAGESSMEARACTALSPEDLTDTEWFYVLC
GsRf_1          TRKTTQPAEITAEEAALERSQQLMELYQTLFAGESSMEARACTALSPEDLTDTEWFYVLC
WsWf_1          TRKTTQPAEITAEEAALERSQQLMELYQTLFAGESSMEARACTALSPEDLTDTEWFYVLC
WsWf_2          TRKTTQPAEITAEEAALERSQQLMELYQTLFAGESSMEARACTALSPEDLTDTEWFYVLC
RsWf_1          TRKTTQPAEITAEEAALERSQQLMELYQTLFAGESSMEARASTALSPEDLTDTEWFYVLC
RsWf_2          TRKTTQPAEITAEEAALERSQQLMELYQTLFAGESSMEARASTALSPEDLTDTEWFYVLC
AtTT8           TRKTTQPAEVTAEEAALERSQQLRELYETLLAGESTSEARACTALSPEDLTETEWFYLMC
                *********:************* ***:**:****: ****.*********:*****::*
```

```
RsRf_1            LTYSFEPPSGMPGKAYARRKQVWMSGVNEVDSKIFSRAISAKSAKIQTVVCIPVLDGVLE
RsRf_2            LTYSFEPPSGMPGKAYARRKQVWMSGVNEVDSKIFSRAISAKSAKIQTVVCIPVLDGVLE
GsRf_2            LTYSFEPPSGMPGKAYARRKQVWMSGVNEVDSKIFSRAISAKSAKIQTVVCIPVLDGVLE
GsRf_1            LTYSFEPPSGMPGKAYARRKQVWMSGVNEVDSKIFSRAISAKSAKIQTVVCIPVLDGVLE
WsWf_1            LTYSFEPPSGMPGKAYARRKQVWMSGVNEVDSKIFSRAISAKSAKIQTVVCIPVLDGVLE
WsWf_2            LTYSFEPPSGMPGKAYARRKQVWMSGVNEVDSKIFSRAISAKSAKIQTVVCIPVLDGVLE
RsWf_1            LTYSFEPPSGMPGKAYARRKQVWMSGVNEVDSKIFSRAISAKSAKIQTVVCIPVLDGVLE
RsWf_2            LTYSFEPPSGMPGKAYARRKQVWMSGVNEVDSKIFSRAISAKSAKIQTVVCIPVLDGVLE
AtTT8             VSFSFPPPSGMPGKAYARRKHVWLSGANEVDSKTFSRAILAKSAKIQTVVCIPMLDGVVE
                  :::** **************:**:**.****** ***** *************:****:*

RsRf_1            IGTTNKVKENEEFVEHMKSFFQNHPKSNTKPALFEHSINEEHEEDEE----VEEMTMSEE
RsRf_2            IGTTNKVKENEEFVEHMKSFFQNHPKSNTKPALFEHSINEEHEEDEE----VEEMTMSEE
GsRf_2            IGTTNKVKENEEFVEHMKSFFQNHPKSNTKPALFEHSINEEHEEDEE----VEEMTMSEE
GsRf_1            IGTTNKVKENEEFVEHMKSFFQNHPKSNTKPALFEHSINEEHEEDEE----VEEMTMSEE
WsWf_1            IGTTNKVKENEEFVEHIKSFFQNHPKSNTKPALFEHSINEEHEEDEE----VEEMTMSEE
WsWf_2            IGTTNKVKENEEFVEHIKSFFQNHPKSNTKPALFEHSINEEHEEDEE----VEEMTMSEE
RsWf_1            IGTTNKVKENEEFVEHIKSFFQNHPKSNTKPALFEHSINEDHEEDEEE---VEEMTMSEE
RsWf_2            IGTTNKVKENEEFVEHIKSFFQNHPKSNTKPALFEHSINEDHEEDEEE---VEEMTMSEE
AtTT8             LGTTKKVREDVEFVELTKSFFYDHCKTNPKPALSEHSTYEVHEEAEDEEEVEEEMTMSEE
                  :***:**:*: ****  **** :* *:*.**** ***  * *** *:     ********

RsRf_1            IRLGSPDDDDVSNQNLLSDFHIEAPSSLDTQMDMMNLMEEGGNYSQTVSTLLMSQLPNLL
RsRf_2            IRLGSPDDDDVSNQNLLSDFHIEAPSSLDTQMDMMNLMEEGGNYSQTVSTLLMSQLPNLL
GsRf_2            IRLGSPDDDDVSNQNLLSDFHIEAPSSLDTQMDMMNLMEEGGNYSQTVSTLLMSQLPNLL
GsRf_1            IRLGSPDDDDVSNQNLLSDFHIEAPSSLDTQMDMMNLMEEGGNYSQTVSTLLMSQLPNLL
WsWf_1            IRLGSPDDDDVSNQNLLSDFHIEAPNSLDTQMDMMNLMEEGGSYSQTVSTLLMSQLPNLL
WsWf_2            IRLGSPDDDDVSNQNLLSDFHIEAPNSLDTQMDMMNLMEEGGSYSQTVSTLLMSQLPNLL
RsWf_1            IRLGSPDDDDVSNQNLLSDFHIEAPNSLDTQMDMMNLMEEGGNYSQTVSTLLMSQLPNLL
RsWf_2            IRLGSPDDDDVSNQNLLSDFHIEAPNSLDTQMDMMNLMEEGGNYSQTVSTLLMSQLPNLL
AtTT8             MRLGSPDDEDVSNQNLHSDLHIESTHTLDTHMDMMNLMEEGGNYSQTVTTLLMSHPTSLL
                  :*******:******* **:***:. :***:***********.*****:*****: ..**

RsRf_1            SDSVSTSSYVQSSFVSWRVENVKEHQQYQREEKASSSSSSQWMLKHMILRVPLLHENTKN
RsRf_2            SDSVSTSSYVQSSFVSWRVENVKEHQQYQREEKASSSSSSQWMLKHMILRVPLLHENTKN
GsRf_2            SDSVSTSSYVQSSFVSWRVENVKEHQQYQREEKASSSSSSQWMLKHMILRVPLLHENTKN
GsRf_1            SDSVSTSSYVQSSFVSWRVENVKEHQQYQREEKASSSSSSQWMLKHMILRVPLLHENTKN
WsWf_1            SDSVSTSSYVQSSFVSWRVENVKEHQQYQREEKASSSSSSQWMLKHMILRVPLLHENTKN
WsWf_2            SDSVSTSSYVQSSFVSWRVENVKEHQQYQREEKASSSSSSQWMLKHMILRVPLLHENTKN
RsWf_1            SDSVSTSSYVQSSFVSWRVENVKEHQQYQREEKASSSSSSQWMLKHMILRVPLLHENTKN
RsWf_2            SDSVSTSSYVQSSFVSWRVENVKEHQQYQREEKASSSSSSQWMLKHMILRVPLLHENTKN
AtTT8             SDSVSTSSYIQSSFATWRVENGKEHQQVKTAP------SSQWVLKQMIFRVPFLHDNTKD
                  *********:****.:***** ***** :         ****:**:**:***:**:***:

RsRf_1            KRVPREELNHVVAERRRREKLNERFITLRSLVPFVTKMDKVSILGDTIDYVNHLCKRIHE
RsRf_2            KRVPREELNHVVAERRRREKLNERFITLRSLVPFVTKMDKVSILGDTIDYVNHLCKRIHE
GsRf_2            KRVPREELNHVVAERRRREKLNERFITLRSLVPFVTKMDKVSILGDTIDYVNHLCKRIHE
GsRf_1            KRVPREELNHVVAERRRREKLNERFITLRSLVPFVTKMDKVSILGDTIDYVNHLCKRIHE
WsWf_1            KRVPREELNHVVAERRRREKLNERFITLRSLVPFVTKMDKVSILGDTIDYVNHLCKRIHE
WsWf_2            KRVPREELNHVVAERRRREKLNERFITLRSLVPFVTKMDKVSILGDTIDYVNHLCKRIHE
RsWf_1            KRVPREELNHVVAERRRREKLNERFITLRSLVPFVTKMDKVSILGDTIDYVNHLCKRIHE
RsWf_2            KRVPREELNHVVAERRRREKLNERFITLRSLVPFVTKMDKVSILGDTIDYVNHLCKRIHE
AtTT8             KRLPREDLSHVVAERRRREKLNEKFITLRSMVPFVTKMDKVSILGDTIAYVNHLRKRVHE
                  **:***:*.**************:******:***************** ***** **:**

RsRf_1            LESTHHEPNQKRMRIGKGRTWEEVEVSIIESDVLLEMRCEYRDGLLLNILQVLKELGIET
RsRf_2            LESTHHEPNQKRMRIGKGRTWEEVEVSIIESDVLLEMRCEYRDGLLLNILQVLKELGIET
GsRf_2            LESTHHEPNQKRMRIGKGRTWEEVEVSIIESDVLLEMRCEYRDGLLLNILQVLKELGIET
GsRf_1            LESTHHEPNQKRMRIGKGRTWEEVEVSIIESDVLLEMRCEYRDGLLLNILQVLKELGIET
WsWf_1            LESTHHEPNQKRMRIGKGRTWEEVEVSIIESDVLLEMRCEYRDGLLLNILQVLKELGIET
WsWf_2            LESTHHEPNQKRMRIGKGRTWEEVEVSIIESDVLLEMRCEYRDGLLLNILQVLKELGIET
RsWf_1            LESTHHEPNQKRMRIGKGRTWEEVEVSIIESDVLLEMRCEYRDGLLLNILQVLKELGIET
RsWf_2            LESTHHEPNQKRMRIGKGRTWEEVEVSIIESDVLLEMRCEYRDGLLLNILQVLKELGIET
AtTT8             LENTHHEQQHKRTRTCKRKTSEEVEVSIIENDVLLEMRCEYRDGLLLDILQVLHELGIET
                  **.**** ::** *  * :* *********.****************:*****:******

RsRf_1            TAVHTAVNDHDFEAEIRAKVRGKKPTIAEVKIAIHQIISQNKL-
RsRf_2            TAVHTAVNDHDFEAEIRAKVRGKKPTIAEVKIAIHQIISQNKL-
GsRf_2            TAVHTAVNDHDFEAEIRAKVRGKKPTIAEVKIAIHQIISQNKL-
GsRf_1            TAVHTAVNDHDFEAEIRAKVRGKKPTIAEVKIAIHQIISQNKL-
WsWf_1            TAVHTAVNDHDFEAEIRAKVRGKKPTIAEVKIAIHQIISQNKL-
WsWf_2            TAVHTAVNDHDFEAEIRAKVRGKKPTIAEVKIAIHQIISQNKL-
RsWf_1            TAVHTAVNDHDFEAEIRAKVRGKKPTIAEVKIAIHQIISQNKL-
RsWf_2            TAVHTAVNDHDFEAEIRAKVRGKKPTIAEVKIAIHQIISQNKL-
AtTT8             TAVHTSVNDHDFEAEIRAKVRGKKASIAEVKRAIHQVIIHDTNL
                  *****:******************.:***** ****:* ::.
```

```
>AtTT8
MDESSIIPAEKVAGAEKKELQGLLKTAVQSVDWTYSVFWQFCPQQRVLVWGNGYYNGAIKTRKTTQPAEVTAEEAALERSQQLRELYETLLAGESTSEARACTALSPEDLTETEWFYLMCVSFS
FPPPSGMPGKAYARRKHVWLSGANEVDSKTFSRAILAKSAKIQTVVCIPMLDGVVELGTTKKVREDVEFVELTKSFFYDHCKTNPKPALSEHSTYEVHEEAEDEEEVEEEMTMSEEMRLGSPDD
EDVSNQNLHSDLHIESTHTLDTHMDMMNLMEEGGNYSQTVTTLLMSHPTSLLSDSVSTSSYIQSSFATWRVENGKEHQQVKTAPSSQWVLKQMIFRVPFLHDNTKDKRLPREDLSHVVAERRRR
EKLNEKFITLRSMVPFVTKMDKVSILGDTIAYVNHLRKRVHELENTHHEQQHKRTRTCKRKTSEEVEVSIIENDVLLEMRCEYRDGLLLDILQVLHELGIETTAVHTSVNDHDFEAEIRAKVRG
KKASIAEVKRAIHQVIIHDTNL

>RsWf_1
MDESSIIPVWKVIGAEGKEIQGLLKAVVQSVGWTYSLFWQLCPQRRKLVWSSGFYNGAIKTRKTTQPAEITAEEAALERSQQLMELYQTLFAGESSMEARASTALSPEDLTDTEWFYVLCLTYS
FEPPSGMPGKAYARRKQVWMSGVNEVDSKIFSRAISAKSAKIQTVVCIPVLDGVLEIGTTNKVKENEEFVEHIKSFFQNHPKSNTKPALFEHSINEDHEEDEEEVEEMTMSEEIRLGSPDDDDV
SNQNLLSDFHIEAPNSLDTQMDMMNLMEEGGNYSQTVSTLLMSQLPNLLSDSVSTSSYVQSSFVSWRVENVKEHQQYQREEKASSSSSSQWMLKHMILRVPLLHENTKNKRVPREELNHVVAER
RRREKLNERFITLRSLVPFVTKMDKVSILGDTIDYVNHLCKRIHELESTHHEPNQKRMRIGKGRTWEEVEVSIIESDVLLEMRCEYRDGLLLNILQVLKELGIETTAVHTAVNDHDFEAEIRAK
VRGKKPTIAEVKIAIHQIISQNKL

>RsWf_2
MDESSIIPVWKVIGAEGKEIQGLLKAVVQSVGWTYSLFWQLCPQRRKLVWSSGFYNGAIKTRKTTQPAEITAEEAALERSQQLMELYQTLFAGESSMEARASTALSPEDLTDTEWFYVLCLTYS
FEPPSGMPGKAYARRKQVWMSGVNEVDSKIFSRAISAKSAKIQTVVCIPVLDGVLEIGTTNKVKENEEFVEHIKSFFQNHPKSNTKPALFEHSINEDHEEDEEEVEEMTMSEEIRLGSPDDDDV
SNQNLLSDFHIEAPNSLDTQMDMMNLMEEGGNYSQTVSTLLMSQLPNLLSDSVSTSSYVQSSFVSWRVENVKEHQQYQREEKASSSSSSQWMLKHMILRVPLLHENTKNKRVPREELNHVVAER
RRREKLNERFITLRSLVPFVTKMDKVSILGDTIDYVNHLCKRIHELESTHHEPNQKRMRIGKGRTWEEVEVSIIESDVLLEMRCEYRDGLLLNILQVLKELGIETTAVHTAVNDHDFEAEIRAK
VRGKKPTIAEVKIAIHQIISQNKL

>GsRf_1
MDESSIIPVWKVIGAEEKEIQGLLKAVVQSVGWTYSLFWQLCPQRRKLVWSSGFYNGAIKTRKTTQPAEITAEEAALERSQQLMELYQTLFAGESSMEARACTALSPEDLTDTEWFYVLCLTYS
FEPPSGMPGKAYARRKQVWMSGVNEVDSKIFSRAISAKSAKIQTVVCIPVLDGVLEIGTTNKVKENEEFVEHMKSFFQNHPKSNTKPALFEHSINEEHEEDEEVEEMTMSEEIRLGSPDDDDVS
NQNLLSDFHIEAPSSLDTQMDMMNLMEEGGNYSQTVSTLLMSQLPNLLSDSVSTSSYVQSSFVSWRVENVKEHQQYQREEKASSSSSSQWMLKHMILRVPLLHENTKNKRVPREELNHVVAERR
RREKLNERFITLRSLVPFVTKMDKVSILGDTIDYVNHLCKRIHELESTHHEPNQKRMRIGKGRTWEEVEVSIIESDVLLEMRCEYRDGLLLNILQVLKELGIETTAVHTAVNDHDFEAEIRAKV
RGKKPTIAEVKIAIHQIISQNKL

>GsRf_2
MDESSIIPVWKVIGAEEKEIQGLLKAVVQSVGWTYSLFWQLCPQRRKLVWSSGFYNGAIKTRKTTQPAEITAEEAALERSQQLMELYQTLFAGESSMEARACTALSPEDLTDTEWFYVLCLTYS
FEPPSGMPGKAYARRKQVWMSGVNEVDSKIFSRAISAKSAKIQTVVCIPVLDGVLEIGTTNKVKENEEFVEHMKSFFQNHPKSNTKPALFEHSINEEHEEDEEVEEMTMSEEIRLGSPDDDDVS
NQNLLSDFHIEAPSSLDTQMDMMNLMEEGGNYSQTVSTLLMSQLPNLLSDSVSTSSYVQSSFVSWRVENVKEHQQYQREEKASSSSSSQWMLKHMILRVPLLHENTKNKRVPREELNHVVAERR
RREKLNERFITLRSLVPFVTKMDKVSILGDTIDYVNHLCKRIHELESTHHEPNQKRMRIGKGRTWEEVEVSIIESDVLLEMRCEYRDGLLLNILQVLKELGIETTAVHTAVNDHDFEAEIRAKV
RGKKPTIAEVKIAIHQIISQNKL

>RsRf_1
MDESSIIPVWKVIGAEEKEIQGLLKAVVQSVGWTYSLFWQLCPQRRKLVWSSGFYNGAIKTRKTTQPAEITAEEAALERSQQLMELYQTLFAGESSMEARACTALSPEDLTDTEWFYVLCLTYS
FEPPSGMPGKAYARRKQVWMSGVNEVDSKIFSRAISAKSAKIQTVVCIPVLDGVLEIGTTNKVKENEEFVEHMKSFFQNHPKSNTKPALFEHSINEEHEEDEEVEEMTMSEEIRLGSPDDDDVS
NQNLLSDFHIEAPSSLDTQMDMMNLMEEGGNYSQTVSTLLMSQLPNLLSDSVSTSSYVQSSFVSWRVENVKEHQQYQREEKASSSSSSQWMLKHMILRVPLLHENTKNKRVPREELNHVVAERR
RREKLNERFITLRSLVPFVTKMDKVSILGDTIDYVNHLCKRIHELESTHHEPNQKRMRIGKGRTWEEVEVSIIESDVLLEMRCEYRDGLLLNILQVLKELGIETTAVHTAVNDHDFEAEIRAKV
RGKKPTIAEVKIAIHQIISQNKL

>RsRf_2
MDESSIIPVWKVIGAEEKEIQGLLKAVVQSVGWTYSLFWQLCPQRRKLVWSSGFYNGAIKTRKTTQPAEITAEEAALERSQQLMELYQTLFAGESSMEARACTALSPEDLTDTEWFYVLCLTYS
FEPPSGMPGKAYARRKQVWMSGVNEVDSKIFSRAISAKSAKIQTVVCIPVLDGVLEIGTTNKVKENEEFVEHMKSFFQNHPKSNTKPALFEHSINEEHEEDEEVEEMTMSEEIRLGSPDDDDVS
NQNLLSDFHIEAPSSLDTQMDMMNLMEEGGNYSQTVSTLLMSQLPNLLSDSVSTSSYVQSSFVSWRVENVKEHQQYQREEKASSSSSSQWMLKHMILRVPLLHENTKNKRVPREELNHVVAERR
RREKLNERFITLRSLVPFVTKMDKVSILGDTIDYVNHLCKRIHELESTHHEPNQKRMRIGKGRTWEEVEVSIIESDVLLEMRCEYRDGLLLNILQVLKELGIETTAVHTAVNDHDFEAEIRAKV
RGKKPTIAEVKIAIHQIISQNKL

>WsWf_1
MDESSIIPVWKVIGAEEKEIQGLLKAVVQSVGWTYSLFWQLCPQRRKLVWSSGFYNGAIKTRKTTQPAEITAEEAALERSQQLMELYQTLFAGESSMEARACTALSPEDLTDTEWFYVLCLTYS
FEPPSGMPGKAYARRKQVWMSGVNEVDSKIFSRAISAKSAKIQTVVCIPVLDGVLEIGTTNKVKENEEFVEHIKSFFQNHPKSNTKPALFEHSINEEHEEDEEVEEMTMSEEIRLGSPDDDDVS
NQNLLSDFHIEAPNSLDTQMDMMNLMEEGGSYSQTVSTLLMSQLPNLLSDSVSTSSYVQSSFVSWRVENVKEHQQYQREEKASSSSSSQWMLKHMILRVPLLHENTKNKRVPREELNHVVAERR
RREKLNERFITLRSLVPFVTKMDKVSILGDTIDYVNHLCKRIHELESTHHEPNQKRMRIGKGRTWEEVEVSIIESDVLLEMRCEYRDGLLLNILQVLKELGIETTAVHTAVNDHDFEAEIRAKV
RGKKPTIAEVKIAIHQIISQNKL

>WsWf_2
MDESSIIPVWKVIGAEEKEIQGLLKAVVQSVGWTYSLFWQLCPQRRKLVWSSGFYNGAIKTRKTTQPAEITAEEAALERSQQLMELYQTLFAGESSMEARACTALSPEDLTDTEWFYVLCLTYS
FEPPSGMPGKAYARRKQVWMSGVNEVDSKIFSRAISAKSAKIQTVVCIPVLDGVLEIGTTNKVKENEEFVEHIKSFFQNHPKSNTKPALFEHSINEEHEEDEEVEEMTMSEEIRLGSPDDDDVS
NQNLLSDFHIEAPNSLDTQMDMMNLMEEGGSYSQTVSTLLMSQLPNLLSDSVSTSSYVQSSFVSWRVENVKEHQQYQREEKASSSSSSQWMLKHMILRVPLLHENTKNKRVPREELNHVVAERR
RREKLNERFITLRSLVPFVTKMDKVSILGDTIDYVNHLCKRIHELESTHHEPNQKRMRIGKGRTWEEVEVSIIESDVLLEMRCEYRDGLLLNILQVLKELGIETTAVHTAVNDHDFEAEIRAKV
RGKKPTIAEVKIAIHQIISQNKL
```

Figure S1. Nucleotide sequence of the *RsTT8* gene isolated from four radish inbred lines. Shaded regions indicate exons. Yellow: cis-acting sequence; red: SNP or InDel between red-fleshed and white-fleshed genotypes; blue: white skin/white flesh (WsWf)-specific SNP or InDel; green: red skin/white flesh (RsWf)-specific SNP or InDel; brown: primer sequences; red letter: key nucleotide for domain; numbers: relative to the transcription start site (TSS).
